# Functional Limitations and Exercise Intolerance in Patients With Post-COVID Condition: A Randomized Crossover Clinical Trial

**DOI:** 10.1001/jamanetworkopen.2024.4386

**Published:** 2024-04-04

**Authors:** Andrea Tryfonos, Kaveh Pourhamidi, Gustav Jörnåker, Martin Engvall, Lisa Eriksson, Sara Elhallos, Nicole Asplund, Mirko Mandić, Patrik Sundblad, Atif Sepic, Eric Rullman, Lars Hyllienmark, Helene Rundqvist, Tommy R. Lundberg, Thomas Gustafsson

**Affiliations:** 1 Division of Clinical Physiology, Department of Laboratory Medicine, Karolinska Institutet, Stockholm, Sweden; 2 Department of Life Sciences, School of Sciences, European University Cyprus, Nicosia, Cyprus; 3 Department of Clinical Neurophysiology, Karolinska University Hospital, Stockholm, Sweden; 4 Unit of Clinical Physiology, Karolinska University Hospital, Stockholm, Sweden

## Abstract

**Importance:**

Many patients with post-COVID condition (PCC) experience persistent fatigue, muscle pain, and cognitive problems that worsen after exertion (referred to as postexertional malaise). Recommendations currently advise against exercise in this population to prevent symptom worsening; however, prolonged inactivity is associated with risk of long-term health deterioration.

**Objective:**

To assess postexertional symptoms in patients with PCC after exercise compared with control participants and to comprehensively investigate the physiologic mechanisms underlying PCC.

**Design, Setting, and Participants:**

In this randomized crossover clinical trial, nonhospitalized patients without concomitant diseases and with persistent (≥3 months) symptoms, including postexertional malaise, after SARS-CoV-2 infection were recruited in Sweden from September 2022 to July 2023. Age- and sex-matched control participants were also recruited.

**Interventions:**

After comprehensive physiologic characterization, participants completed 3 exercise trials (high-intensity interval training [HIIT], moderate-intensity continuous training [MICT], and strength training [ST]) in a randomized order. Symptoms were reported at baseline, immediately after exercise, and 48 hours after exercise.

**Main Outcomes and Measures:**

The primary outcome was between-group differences in changes in fatigue symptoms from baseline to 48 hours after exercise, assessed via the visual analog scale (VAS). Questionnaires, cardiopulmonary exercise testing, inflammatory markers, and physiologic characterization provided information on the physiologic function of patients with PCC.

**Results:**

Thirty-one patients with PCC (mean [SD] age, 46.6 [10.0] years; 24 [77%] women) and 31 healthy control participants (mean [SD] age, 47.3 [8.9] years; 23 [74%] women) were included. Patients with PCC reported more symptoms than controls at all time points. However, there was no difference between the groups in the worsening of fatigue in response to the different exercises (mean [SD] VAS ranks for HIIT: PCC, 29.3 [19.5]; controls, 28.7 [11.4]; *P* = .08; MICT: PCC, 31.2 [17.0]; controls, 24.6 [11.7]; *P* = .09; ST: PCC, 31.0 [19.7]; controls, 28.1 [12.2]; *P* = .49). Patients with PCC had greater exacerbation of muscle pain after HIIT (mean [SD] VAS ranks, 33.4 [17.7] vs 25.0 [11.3]; *P* = .04) and reported more concentration difficulties after MICT (mean [SD] VAS ranks, 33.0 [17.1] vs 23.3 [10.6]; *P* = .03) compared with controls. At baseline, patients with PCC showed preserved lung and heart function but had a 21% lower peak volume of oxygen consumption (mean difference: −6.8 mL/kg/min; 95% CI, −10.7 to −2.9 mL/kg/min; *P* < .001) and less isometric knee extension muscle strength (mean difference: −37 Nm; 95% CI, −67 to −7 Nm; *P* = .02) compared with controls. Patients with PCC spent 43% less time on moderate to vigorous physical activity (mean difference, −26.5 minutes/d; 95% CI, −42.0 to −11.1 minutes/d; *P* = .001). Of note, 4 patients with PCC (13%) had postural orthostatic tachycardia, and 18 of 29 (62%) showed signs of myopathy as determined by neurophysiologic testing.

**Conclusions and Relevance:**

In this study, nonhospitalized patients with PCC generally tolerated exercise with preserved cardiovascular function but showed lower aerobic capacity and less muscle strength than the control group. They also showed signs of postural orthostatic tachycardia and myopathy. The findings suggest cautious exercise adoption could be recommended to prevent further skeletal muscle deconditioning and health impairment in patients with PCC.

**Trial Registration:**

ClinicalTrials.gov Identifier: NCT05445830

## Key Points

**Question:**

Do nonhospitalized patients experiencing post-COVID condition (PCC) have exaggerated postexercise symptoms after high-intensity interval training (HIIT), moderate-intensity continuous training (MICT), and strength training (ST)?

**Findings:**

In this randomized crossover clinical trial of 31 patients with PCC and 31 matched control participants, the exercise response was largely comparable between groups, with no profound symptom exacerbation. Patients with PCC reported more muscle pain after HIIT and concentration problems after MICT and had lower aerobic capacity and less muscle strength; 62% showed myopathic signs.

**Meaning:**

The findings suggest that cautious exercise rehabilitation should be recommended to prevent further deconditioning among patients with PCC.

## Teaser

This randomized crossover clinical trial investigates postexercise fatigue and physiologic characteristics among nonhospitalized patients with post-COVID condition compared with age- and sex-matched healthy controls.

## Introduction

The COVID-19 pandemic has left a significant number of people experiencing longer-term health problems despite initially recovering from acute SARS-CoV-2 infection. The constellation of symptoms that these patients continue to experience after 3 or more months has been termed post-COVID condition (PCC) by the World Health Organization (WHO) and affects an estimated 10% to 20% of those infected with SARS-CoV-2, including nonhospitalized individuals.^1,2^ The most common symptoms include persistent fatigue, myalgia, dyspnea, and neurologic or cognitive dysfunction.^1^ These symptoms worsen after physical exertion, a phenomenon described as postexertional malaise (PEM)^1^ or postexertional symptom exacerbation.^1,3^

As a result of the reported exercise intolerance, key public health organizations, including the WHO, have advised against rehabilitation based on graded exercise in patients experiencing PEM to avoid symptom exacerbation.^3,4,5^ This has led many health care professionals to be reluctant to incorporate exercise into rehabilitation programs for patients with PCC. However, there is ample evidence that physical inactivity negatively impacts health, including functional impairment within weeks and increased risk of cardiometabolic disease in the long term.^6^ The latter represents a significant burden on health care systems worldwide.^6^

While several plausible factors have been proposed to explain exercise intolerance in individuals with PCC, including muscle atrophy, physical deconditioning, dysautonomia, and increased inflammation,^7,8^ current data are limited because most studies have been retrospective analyses of referred patients with only 1 to 2 examinations per study.^9,10^ Most also lacked healthy controls for comparison and focused predominantly on patients hospitalized for COVID-19 or mixed cohorts, which often included individuals with concomitant diseases.^1^ Hospitalization and/or intensive care treatment alone can significantly impair physical performance,^11^ primarily through muscle wasting and even critical illness myopathy.^12^ To elucidate the severity and specific mechanisms leading to exercise intolerance following SARS-CoV-2 infection, it is important to prospectively recruit in a controlled design and comprehensively investigate multiple factors in nonhospitalized patients with PEM.

Accordingly, the primary aim of this study was to investigate exercise intolerance in patients with well-defined PCC without prior comorbidities compared with age- and sex-matched healthy controls. Acute responses to 3 different commonly prescribed types of exercise (high-intensity interval training [HIIT], moderate-intensity continuous training [MICT], and strength training [ST]) were investigated in a randomized crossover design to assess whether exercise exacerbates symptoms and whether a particular type of exercise is preferable for patients with PCC. The secondary aim was to investigate the proposed physiologic mechanisms underlying PCC through a comprehensive characterization of physiologic functions.

## Methods

### Study Design, Setting, and Participants

This prospective randomized crossover clinical trial (NCT05445830) was approved by the Swedish Ethical Review Authority and conformed to the Declaration of Helsinki.^13^ The study followed the Consolidated Standards of Reporting Trials (CONSORT) guideline for randomized clinical trials (the trial protocol is given in Supplement 1). All participants gave written informed consent.

Patients with PCC were recruited from September 2022 to July 2023 via advertisements (Karolinska Institutet, Swedish Post–COVID-19 Patients’ Association) and the post–COVID-19 outpatient clinic at Karolinska University Hospital. Inclusion criteria were (1) age between 18 and 64 years; (2) laboratory-confirmed SARS-CoV-2 infection; (3) persistent PEM symptoms for 3 or more months, verified by the DePaul Symptom Questionnaire^14^; (4) no hospitalization for COVID-19; (5) no history of cardiovascular or respiratory disease, generalized anxiety disorder, or somatic symptom disorder; and (6) no symptoms before March 2020. An equal number of age- and sex-matched healthy controls were also recruited from the general population.

**Figure 1.**
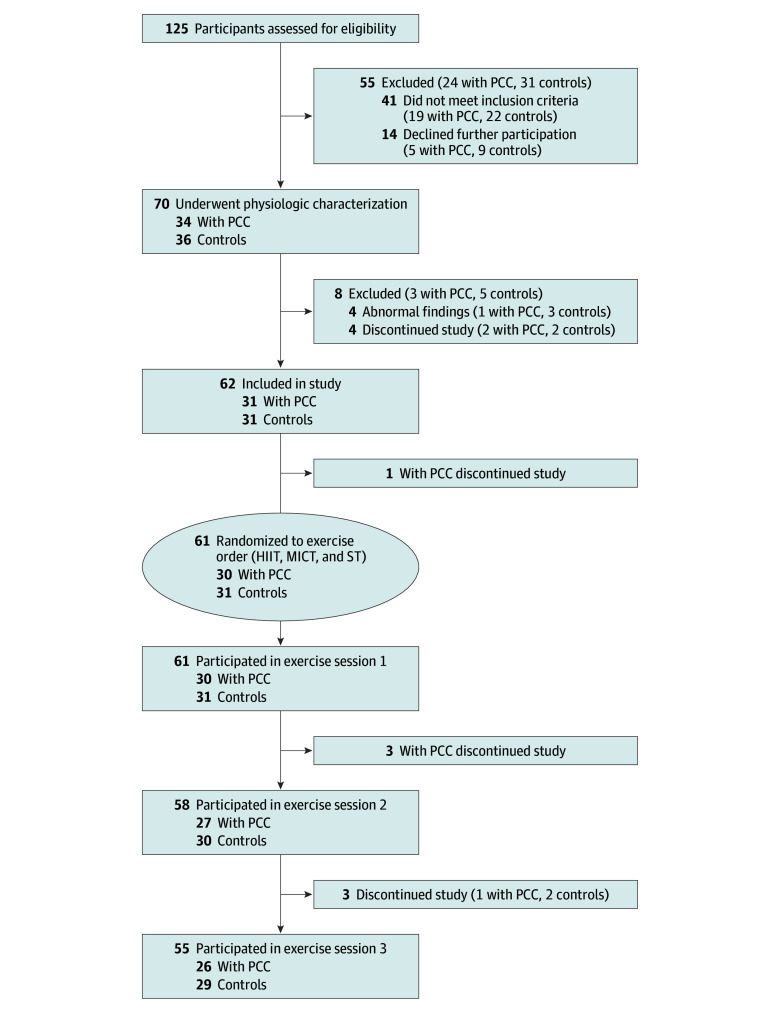
Study Flowchart HIIT indicates high-intensity interval training; MICT, moderate-intensity continous training; PCC, post-COVID condition; and ST, strength training.

All participants underwent comprehensive physiologic characterization at clinical physiology and neurophysiology units at Karolinska University Hospital. They then completed 3 acute exercise sessions in a randomized, counterbalanced order: HIIT, MICT, and ST (Figure 1). The primary outcome was the difference in change in fatigue (from baseline to 48 hours after exercise) between groups, which was assessed within a panel of 10 symptoms associated with PEM and PCC using the visual analog scale (VAS; score range, 0 [no feeling] to 10 [worst possible feeling]).

REDCap software was used for random allocation and data collection. The data analysis was blinded, while the study personnel were blinded as far as possible during data collection. Adverse events associated with the study measures were recorded and clarified in consultation with the responsible physician.

### Exercise Responses

All participants completed 3 exercise sessions (HIIT, MICT, and ST) in a randomized, balanced order with an approximately 2- to 4-week washout between sessions. Maximal workload from the baseline cardiopulmonary exercise testing (CPET) was used to determine exercise intensity for HIIT: 5 × 1-minute cycling at 90% maximal workload and a Borg Rating of Perceived Exertion (RPE) score higher than 16,^15^ with 1-minute passive rest between intervals. Moderate-intensity continuous training consisted of 30-minute continuous cycling at 50% maximal workload (RPE score, 12-14). Strength training included 3 exercises: dead lifts (Kbox; Exxentric AB), push-ups, and knee extensions using flywheel technology (nHance), each with 3 sets of 10 repetitions and a 3-minute rest between sets.

Exercise sessions were closely monitored, including continuous measurements of oxygen saturation, heart rate (MAX-FAST-I; Nellcor), blood pressure (Minidop ES-100VX; Hadeco), RPE score, and lactate concentration. Participants completed the following questionnaires to assess symptoms of PEM before, immediately after, and 48 hours after exercise: VAS for 10 PCC symptoms (fatigue, muscle pain, joint pain, fever, chills, lymph node discomfort, sore throat, headache, memory, and concentration), Multidimensional Fatigue Inventory,^16^ Profile of Mood States,^17^ and Somatic and Psychological Health Report.^18^ The 48-hour follow-up was selected based on peak fatigue at 24 to 72 hours in patients with myalgic encephalomyelitis–chronic fatigue syndrome.^19^ Blood samples were collected at the same time points to quantify creatine kinase (CK) (cobas c 701; Roche Diagnostics) and interleukin 6 (IL-6) (D6050; R&D Systems) levels. At the 48-hour follow-up, participants also performed CPET.

### Physiologic Characterization

All participants underwent standard spirometry (MasterScreen PFT; Jaeger) and echocardiography (Vivid E95 System; GE HealthCare) according to clinical guidelines. Cardiopulmonary exercise testing was performed on a cycle ergometer (Rodby Innovation AB; increments of 10-25 W/min) with a continuous gas analyzer (Vyntus CPX; Jaeger) to determine aerobic capacity as peak volume of oxygen consumption (V̇O_2_) and ventilatory threshold (VT) using the V-slope method (verified by 2 independent observers, G.J. and N.A.).^20^ Lactate concentration was measured at the earlobe before and every 2 minutes during CPET (Lactate Scout+; SensLab GmbH); the onset of blood lactate accumulation at 4 mmol/L was assessed.^21^

Orthostatic tolerance was assessed by the head-up tilt test (HUTT) with continuous hemodynamic monitoring (Finapres NOVA; Finapres Medical Systems) according to guidelines.^22^ Clinical outcomes, including postural orthostatic tachycardia syndrome (POTS), were determined based on consensus criteria.^23^

Arterial stiffness was assessed by aortic pulse wave velocity using arteriography (TensioMed). The 6-minute walk test assessed physical function,^24^ whereas upper- and lower-body muscle strength were measured using handgrip dynamometry (5030-J1; Sammons Preston Rolyan) and isokinetic dynamometry (System 4 Pro; BioDex Medical Systems), respectively. Blood tests, including for biochemical markers, were performed (Table 1), and blood volume was estimated by carbon monoxide rebreathing.^25^ The following questionnaires were used: Godin-Shephard Leisure-Time Physical Activity,^26^ 36-Item Short Form Health Survey (SF-36),^27^ Modified Medical Research Council Dyspnea Scale, and Post–COVID-19 Functional Status.^28^ Physical activity was monitored for 7 days via accelerometer (wGT3X-BT; ActiGraph) and evaluated with ActiLife software (ActiGraph) using guidelines from Choi et al (2011).^29^

**Table 1.** Characteristics and Results From the Physiologic Assessment Among Patients With PCC and Age- and Sex-Matched Healthy Controls

Characteristic	Participants^a^		*P* value
	With PCC (n = 31)	Controls (n = 31)	
Age, y	46.6 (10.0)	47.3 (8.9)	.78
Sex, No. (%)			
Men	7 (23)	8 (26)	.77
Women	24 (77)	23 (74)	.77
BMI	25.3 (3.0)	25.1 (3.5)	.82
Body surface area, m^2^	1.8 (0.2)	1.9 (0.2)	.66
Diabetes, No. (%)	0	2 (6)	.15
Hypertension, No. (%)	5 (16)	4 (13)	.72
Smoker, No. (%)	1 (3)	2 (6)	.55
β-Blocker use at baseline, No. (%)	9 (29)	2 (6)	.02
Verified SARS-CoV-2 infection, No. (%)	31 (100)	16 (52)	>.99
COVID-19 vaccinated, No. (%)	28 (90)	28 (90)	
GSLTPAQ, score^b^			
Before COVID-19	50.5 (19.2)	NA	NA
Baseline	23.3 (15.8)	51.4 (27.3)	<.001
SF-36 questionnaire score^c^			
Physical function	49.9 (19.3)	92.0 (4.5)	<.001
Physical role	12.9 (24.0)	96.0 (18.4)	<.001
Emotional role	75.3 (40.4)	98.9 (6.0)	<.001
Mental health	68.1 (18.7)	85.1 (9.8)	<.001
Social function	41.1 (25.3)	95.6 (12.7)	<.001
Bodily pain	45.8 (25.3)	88.9 (15.9)	<.001
Vitality	23.1 (20.0)	76.3 (13.5)	<.001
General health	39.5 (18.6)	84.0 (10.2)	<.001
Physical function: 6-min walk, m^d^	458 (82)	493 (65)	.07
Muscle strength			
Isokinetic 60 deg/s, Nm	135.6 (53.6)	154.6 (50.3)	.17
Isokinetic 210 deg/s, Nm	93.9 (37.3)	102.0 (27.9)	.40
Isometric 120 deg, Nm	150.1 (53.6)	186.7 (64.4)	.02
Handgrip MVC, kg	36.2 (10.4)	40.5 (16.5)	.23
Accelerometers^e^			
Moderate to vigorous physical activity, min/d	34.8 (28.1)	61.3 (30.0)	.001
Total physical activity, min/d	316.6 (257.9)	306.3 (75.9)	.84
Sedentary time, min/d	571.0 (81.3)	557.8 (75.3)	.53
Plasma volume, mL/kg^f^	41.9 (7.8)	45.3 (5.4)	.09
Blood volume, mL/kg^f^	67.6 (12.7)	72.9 (9.6)	.12
Blood biomarker levels^g^			
Leukocytes, μ/L	5600 (1300)	5500 (1600)	.87
Erythrocytes, ×10^6^/μL	4.4 (0.4)	4.4 (0.4)	.98
Thrombocytes, ×10^3^/μL	249.7 (53.4)	259.5 (74.4)	.57
Hemoglobin, g/dL	13.31 (1.05)	13.23 (1.01)	.79
Erythrocyte volume fraction	0.40 (0.03)	0.41 (0.03)	.51
Erc(B)-MCH, fL	30.3 (1.4)	30.2 (1.3)	.66
Erc(B)-MCV, pg	90.9 (4.1)	92.2 (3.7)	.21
C-reactive protein, mg/dL	0.098 (0.09)	0.14 (0.16)	.22
Glucose, mg/dL	95.5 (10.8)	93.7 (10.8)	.25
Triglycerides, mg/dL	124.2 (106.3)	79.7 (44.3)	.07
Total cholesterol, mg/dL	208.8 (42.5)	197.2 (30.9)	.36
Iron, μg/dL	101.1 (34.1)	100.6 (37.4)	.95
Transferrin, mg/dL	246 (32)	248 (41)	.85
Transferrin saturation, %	0.30 (0.11)	0.31 (0.13)	.82
Spirometry findings			
Forced vital capacity, L	4.1 (1.0)	4.4 (1.2)	.30
FEV_1_, L	3.2 (0.8)	3.3 (1.0)	.68
Vital capacity, L	4.0 (0.9)	4.4 (1.2)	.15
FEV_1_/vital capacity	0.76 (0.15)	0.76 (0.07)	.82
Residual volume, L	2.1 (0.4)	2.3 (0.6)	.11
Total lung capacity, L	6.1 (1.1)	6.7 (1.6)	.10
Residual volume/total lung capacity	34.6 (5.3)	34.5 (5.1)	.92
Single breath DLCOc, mmol/(min × kPa)	7.8 (2.1)	8.7 (2.4)	.12
Echocardiography findings			
Stroke volume, mL	59.5 (14.2)	69.2 (18.1)	.02
Stroke index, mL/kg	20.9 (3.9)	22.1 (4.8)	.28
LVEF, %	60.3 (2.7)	60.7 (3.6)	.66
Cardiac output, L	3.8 (0.9)	4.1 (1.6)	.28
Cardiac index, L/kg	2.1 (0.4)	2.2 (0.5)	.15
LV diameter, mm	47.9 (3.8)	50.5 (5.2)	.03
TAPSE, mm	24.4 (3.8)	26.3 (4.0)	.06
E/A	1.5 (0.5)	1.7 (0.5)	.13
Arteriography findings^h^			
Aortic PWV, m/s	9.1 (1.3)	8.4 (1.4)	.04
CPET findings			
Peak V̇O_2_			
L/min	2.1 (0.7)	2.6 (0.7)	.004
mL/kg/min	29.0 (7.6)	35.8 (7.7)	<.001
Workload peak, W	175 (64)	221 (65)	.005
Peak heart rate, bpm	173 (13)	172 (8)	.81
Respiratory exchange ratio	1.19 (0.08)	1.18 (0.07)	.99
SaO_2_, %			
Rest	98 (1)	98 (1)	.77
Peak	97 (2)	96 (2)	.30
RPE score^i^			
Rest	6.8 (1.8)	6.1 (0.4)	.14
Peak	19.1 (0.8)	18.7 (1.0)	.08
Lactate, mmol/L			
Rest	0.8 (0.4)	0.8 (0.3)	.95
Peak	9.6 (3.2)	10.2 (2.9)	.43
V̇O_2_			
At ventilatory threshold			
mL/kg/min	17.0 (4.4)	19.6 (5.9)	.07
%	57.4 (8.3)	54.9 (10.8)	.33
At OBLA			
mL/kg/min	25.3 (6.1)	29.3 (6.7)	.02
%	86.4 (8.5)	85.5 (8.9)	.70
HUTT^h^			
Normal function, No. (%)	25 (81)	28 (93)	.24
POTS, No. (%)	2 (7)	0	
Borderline POTS, No. (%)	2 (7)	0	
Syncope, No. (%)	2 (7)	1 (3)	
Orthostatic hypotension, No. (%)	0	1 (3)	
Heart rate, /min			
Supine	66 (10)	59 (9)	.006
HUTT	86 (13)	76 (14)	.005
SBP, mm Hg			
Supine	131 (12)	128 (12)	.37
HUTT	131 (16)	126 (16)	.19
DBP, mm Hg			
Supine	80 (10)	78 (10)	.39
HUTT	93 (16)	86 (11)	.07
Heart rate variability, %^d^			
Normal breathing	16.1 (6.8)	17.7 (9.7)	.46
Deep breathing	26.9 (10.5)	32.6 (10.8)	.05
Sympathetic skin response^d^			
Hand			
Elicitability, No. (%)	29 (100)	31 (100)	.12
Latency, s	1.43 (0.13)	1.37 (0.18)	
Foot			
Elicitability, No. (%)	28 (100)	31 (100)	.008
Latency, s	2.07 (0.28)	1.87 (0.26)	
Nerve conduction study findings^d^			
Normal large nerve function, No. (%)	29 (100)	31 (100)	>.99
Sural nerve			
SNAP, μV	23.8 (11.3)	22.0 (10.0)	.53
Conduction velocity, m/s	57.7 (7.9)	56.5 (7.1)	.55
Fibular nerve			
CMAP, mV	5.3 (1.9)	6.1 (2.6)	.20
Conduction velocity, m/s	50.0 (2.7)	49.7 (3.6)	.73
Tibial nerve			
CMAP, mV	14.2 (4.4)	11.8 (3.7)	.02
Conduction velocity, m/s	47.7 (3.5)	48.2 (4.0)	.63
Electromyography findings, No. (%)^j^			
Normal	10 (34)	26 (92)	<.001
Myopathic indices	13 (45)	0	
Myopathic indices borderline	5 (17)	1 (4)	
Neuropathic indices	1 (4)	1 (4)	

Abbreviations: BMI, body mass index (calculated as weight in kilograms divided by height in meters squared); CMAP, compound muscle action potential; CPET, cardiopulmonary exercise testing; DBP, diastolic blood pressure; deg, degrees; DLCOc, diffusion capacity of the lung for carbon monoxide; E/A, early to late ventricular filling velocities ratio; Erc(B)-MCH, erythrocyte mean corpuscular hemoglobin concentration; Erc(B)-MCV, erythrocyte mean corpuscular volume; FEV_1_, forced expiratory volume in the first second; GSLTPAQ, Godin-Shephard Leisure-Time Physical Activity Questionnaire; HUTT, head-up tilt test; LV, left ventricle; LVEF, left ventricular ejection fraction; MVC, maximal voluntary contraction; NA, not applicable; OBLA, onset of blood lactate accumulation; PCC, post-COVID condition; POTS, postural orthostatic tachycardia syndrome; PWV, pulse wave velocity; RPE, Rating of Perceived Exertion; SaO_2_, arterial blood oxygen saturation; SBP, systolic blood pressure; SF-36, 36-Item Short Form Health Survey; SNAP, sensory nerve action potential; TAPSE, tricuspid annular plane systolic excursion.

SI conversion factors: To convert C-reactive protein to mg/L, multiply by 10; erythrocytes to ×10^12^/L, multiply by 1.0; glucose to mmol/L, multiply by 0.0555; hemoglobin to g/L, multiply by 10.0; iron to μmol/L, multiply by 0.179; leukocytes to ×10^9^/L, multiply by 0.001; total cholesterol to mmol/L, multiply by 0.0259; thrombocytes to ×10^9^/L, multiply by 1.0; transferrin to μmol/L, multiply by 0.123; triglycerides to mmol/L, multiply by 0.0113.

^a^ Data are presented as mean (SD) unless otherwise stated.

^b^ Range, 0 to 115, with scores indicating metabolic equivalents of task per minutes of physical activity per week.

^c^ Range, 0 to 100, with higher scores indicating better health-related quality of life in 8 domains.

^d^ Patients with PCC, n = 29; controls, n = 31.

^e^ Patients with PCC, n = 28; controls, n = 29.

^f^ Patients with PCC, n = 25; controls, n = 23.

^g^ Patients with PCC, n = 29; controls, n = 27.

^h^ Patients with PCC, n = 31; controls, n = 30.

^i^ Using the Borg 6 to 20 scale; higher scores indicate more physical exertion.

^j^ PCC, n = 29; controls, n = 28.

Neurophysiologic testing was conducted with the Sierra Summit EMG system (Cadwell Industries, Inc) and included (1) motor and sensory nerve conduction studies in the dominant limbs, (2) heart rate variability during normal and deep breathing, (3) sympathetic skin response in the hands and feet, and (4) needle electromyography (EMG) of 6 muscles using a 35-mm concentric needle (20 Hz to 10 kHz filter). The EMG data were analyzed qualitatively and quantitively for the duration, amplitude, and number of phases of more than 20 motor unit potentials (MUPs). Spontaneous activity was assessed at rest and interference pattern during slight and maximal contraction. The EMG outcomes were classified as (1) normal (MUPs without significant myopathic or neurogenic characteristics), (2) myopathic (>50% of displayed MUPs with short duration, small amplitudes, and polyphasia [≥5 phases]; the outcome was considered definitive myopathic if these characteristics were present in ≥2 muscles), (3) borderline myopathic (myopathic findings observed in only 1 muscle), (4) neurogenic (overrepresentation of MUPs with increased duration and amplitudes with or without increased polyphasia), or (5) mixed (both myopathic and neurogenic indices coexisted).^30^

### Statistical Analysis

Analysis was performed using IBM SPSS, version 29.0 (IBM Corp) according to the intention-to-treat approach. Our a priori estimate (using G*Power, version 3.1; University of Dusseldorf) was that we would need at least 28 patients and 28 controls to detect a moderate effect size (critical *F* = 0.25) for the difference in fatigue between groups using a within-between interaction design with a power of 80% and an α of 5% (Supplement 1). In the final analysis, however, the postexercise symptoms were compared between groups using nonparametric tests due to nonnormal distribution. A mixed linear model (fixed effects: group, time, and exercise; random effects: study identification number, exercise order) was used to analyze differences in continuous data (CPET variables and IL-6 and CK levels) following 3 exercise sessions (HIIT, MICT, and ST) between the 2 groups (patients with PCC, controls). Pairwise comparisons with Bonferroni correction were performed when significant main or interaction effects were detected. For physiologic characterization, χ^2^ tests were used to compare categorical variables between patients with PCC and controls. Continuous variables were compared using 2-tailed Student *t* tests or nonparametric tests (Mann-Whitney, Kruskal-Wallis) when appropriate. Results are presented as means (SDs) or medians (IQRs). Statistical significance was defined as 2-sided *P* < .05.

## Results

### Demographic Data

This study included 62 participants (47 women [76%]; 15 men [24%]), with a mean (SD) age of 47.0 (9.4) years. Specifically, the study cohort consisted of 31 patients with PCC (mean [SD] age, 46.6 [10.0] years; 7 men [23%], 24 women [77%]) and 31 age- and sex-matched healthy controls (mean [SD] age, 47.3 [8.9] years; 8 men [26%], 23 women [74%]) (Table 1 and eTable 1 in Supplement 2). For the PCC group, the mean (SD) symptom duration was 21.6 (9.2) months. Mean (SD) scores on the Post–COVID-19 Functional Status scale and the Modified Medical Research Council Dyspnea Scale were 2.6 (0.8) and 1.9 (1.0), respectively. Of 23 patients with PCC who responded to questions about work status, 18 (78%) were employed full time before SARS-CoV-2 infection, whereas 17 (74%) were on extended sick leave because of PCC (eTable 1 in Supplement 2). Results of the SF-36 health survey showed lower general, physical, emotional, and social function in patients with PCC compared with controls (Table 1). During the study, there was 1 minor adverse event in 1 control participant (3%) (knee pain during isokinetic dynamometry) that resolved with sequelae, and the participant was able to complete the study.

### Postexertional Symptoms

All participants reached the targeted exercise intensity during exercise sessions (eTable 2 in Supplement 2). Four questionnaires (VAS, Multidimensional Fatigue Inventory, Profile of Mood States, and Somatic and Psychological Health Report) showed higher overall symptom scores in patients with PCC compared with controls at all time points and sessions (Table 2 and eTables 3-6 in Supplement 2). To assess persistent symptom exacerbation after each exercise mode, differences in changes from baseline to 48 hours after exercise were calculated and compared between patients with PCC and controls.

**Table 2.** Postexertional Symptoms as Assessed by VAS for 10 Symptoms Before, Immediately After, and 48 Hours After 3 Exercise Sessions Among Patients With PCC and Age- and Sex-Matched Healthy Controls^**a**^

Symptom	VAS score, median (IQR)^b^							*P* value for difference					
	HIIT		MICT			ST		Patients with PCC vs controls^c^			All exercises^d^		
	PCC	Control	PCC	Control	PCC		Control	HIIT	MICT	ST		PCC	Control
Fatigue													
Preexercise	5.0 (3.0-7.5)	0.0 (0.0-2.0)	4.0 (2.0-6.3)	0.0 (0.0-1.0)	5.0 (3.0-6.0)		0.0 (0.0-1.0)	NA	NA	NA		NA	NA
Postexercise	7.5 (6.0-9.0)	3.0 (2.0-4.5)	7.0 (5.8-8.0)	2.0 (0.0-3.5)	7.0 (5.5-8.0)		1.0 (0.0-2.0)	.09	.37	.04		.36	.03
48 h	6.0 (4.0-8.0)	0.0 (0.0-1.8)	4.5 (2.8-7.0)	0.0 (0.0-1.0)	5.0 (4.0-7.0)		0.0 (0.0-2.0)	.88	.09	.49		.87	.33
Muscle pain													
Preexercise	2.0 (1.0-5.5)	0.0 (0.0-0.0)	3.0 (1.0-5.3)	0.0 (0.0-1.0)	2.0 (0.0-4.5)		0.0 (0.0-0.5)	NA	NA	NA		NA	NA
Postexercise	4.0 (2.0-6.8)	0.0 (0.0-2.0)	3.5 (1.8-5.0)	0.0 (0.0-2.0)	4.0 (1.0-7.0)		0.0 (0.0-2.5)	.04	.84	.22		.17	.56
48 h	3.0 (1.0-6.0)	0.0 (0.0-0.0)	2.0 (0.8-4.3)	0.0 (0.0-0.0)	5.0 (1.0-6.0)		1.0 (0.0-3.5)	.04	.96	.20		<.001	<.001
Joint pain													
Preexercise	2.0 (0.0-5.5)	0.0 (0.0-1.0)	2.0 (0.0-6.3)	0.0 (0.0-0.0)	2.0 (0.0-5.0)		0.0 (0.0-0.0)	NA	NA	NA		NA	NA
Postexercise	2.0 (0.0-6.0)	0.0 (0.0-0.0)	2.0 (0.0-4.0)	0.0 (0.0-0.0)	2.0 (0.0-5.0)		0.0 (0.0-0.0)	.10	.27	.14		.17	.35
48 h	2.0 (0.0-6.0)	0.0 (0.0-0.0)	2.0 (0.0-6.0)	0.0 (0.0-0.0)	2.0 (0.0-6.0)		0.0 (0.0-0.5)	.009	.98	.73		.49	.32
Fever													
Preexercise	0.0 (0.0-1.5)	0.0 (0.0-0.0)	0.0 (0.0-2.3)	0.0 (0.0-0.0)	0.0 (0.0-1.0)		0.0 (0.0-0.0)	NA	NA	NA		NA	NA
Postexercise	0.0 (0.0-1.8)	0.0 (0.0-0.0)	0.0 (0.0-3.0)	0.0 (0.0-0.0)	0.0 (0.0-1.0)		0.0 (0.0-0.0)	.78	.20	.46		.48	.22
48 h	0.0 (0.0-1.0)	0.0 (0.0-0.0)	0.0 (0.0-1.3)	0.0 (0.0-0.0)	0.0 (0.0-1.0)		0.0 (0.0-0.0)	>.99	.48	.63		.63	.22
Chills													
Preexercise	0.0 (0.0-1.5)	0.0 (0.0-0.0)	0.0 (0.0-0.3)	0.0 (0.0-0.0)	0.0 (0.0-0.0)		0.0 (0.0-0.0)	NA	NA	NA		NA	NA
Postexercise	0.0 (0.0-0.0)	0.0 (0.0-0.0)	0.0 (0.0-0.0)	0.0 (0.0-0.0)	0.0 (0.0-0.0)		0.0 (0.0-0.0)	.17	.39	.94		.66	.30
48 h	0.0 (0.0-1.5)	0.0 (0.0-0.0)	0.0 (0.0-1.3)	0.0 (0.0-0.0)	0.0 (0.0-0.0)		0.0 (0.0-0.0)	.64	.17	.66		.54	.52
Lymph node discomfort													
Preexercise	0.0 (0.0-1.0)	0.0 (0.0-0.0)	0.0 (0.0-1.3)	0.0 (0.0-0.0)	0.0 (0.0-0.0)		0.0 (0.0-0.0)	NA	NA	NA		NA	NA
Postexercise	0.0 (0.0-0.0)	0.0 (0.0-0.0)	0.0 (0.0-1.0)	0.0 (0.0-0.0)	0.0 (0.0-0.0)		0.0 (0.0-0.0)	.13	.64	.62		.79	.22
48 h	0.0 (0.0-1.0)	0.0 (0.0-0.0)	0.0 (0.0-0.0)	0.0 (0.0-0.0)	0.0 (0.0-0.5)		0.0 (0.0-0.0)	.70	.02	.11		.01	.22
Sore throat													
Preexercise	0.0 (0.0-1.5)	0.0 (0.0-0.0)	0.0 (0.0-1.3)	0.0 (0.0-0.0)	0.0 (0.0-1.0)		0.0 (0.0-0.0)	NA	NA	NA		NA	NA
Postexercise	0.0 (0.0-2.0)	0.0 (0.0-0.0)	0.0 (0.0-1.3)	0.0 (0.0-0.0)	0.0 (0.0-1.0)		0.0 (0.0-0.0)	.60	.33	>.99		.59	.60
48 h	0.0 (0.0-1.0)	0.0 (0.0-0.0)	0.0 (0.0-1.3)	0.0 (0.0-0.0)	0.0 (0.0-1.0)		0.0 (0.0-0.0)	.41	.31	.38		.17	.44
Headache													
Preexercise	2.0 (0.5-5.0)	0.0 (0.0-0.0)	1.0 (0.0-5.0)	0.0 (0.0-0.3)	2.0 (0.0-3.5)		0.0 (0.0-0.0)	NA	NA	NA		NA	NA
Postexercise	2.0 (0.0-5.0)	0.0 (0.0-0.0)	1.0 (0.0-5.3)	0.0 (0.0-0.0)	2.0 (0.0-3.0)		0.0 (0.0-0.0)	.94	.13	.39		.71	.33
48 h	2.0 (0.0-5.0)	0.0 (0.0-0.0)	2.5 (0.0-6.0)	0.0 (0.0-0.0)	1.0 (0.0-4.0)		0.0 (0.0-0.0)	.83	.09	.75		.33	.10
Memory													
Preexercise	3.0 (1.5-6.0)	0.0 (0.0-0.0)	3.0 (0.8-6.0)	0.0 (0.0-0.0)	2.0 (2.0-5.0)		0.0 (0.0-0.0)	NA	NA	NA		NA	NA
Postexercise	3.0 (1.3-5.8)	0.0 (0.0-0.0)	3.0 (2.0-5.3)	0.0 (0.0-0.0)	3.0 (1.0-6.0)		0.0 (0.0-0.0)	.77	.58	.03		.74	.07
48 h	3.0 (1.5-5.5)	0.0 (0.0-0.0)	3.5 (1.0-6.2)	0.0 (0.0-0.0)	3.0 (1.0-5.0)		0.0 (0.0-0.0)	.74	.18	.81		.41	.81
Concentration													
Preexercise	4.0 (2.0-5.5)	0.0 (0.0-1.0)	3.5 (2.0-6.0)	0.0 (0.0-1.0)	4.0 (2.0-5.0)		0.0 (0.0-0.0)	NA	NA	NA		NA	NA
Postexercise	4.0 (2.3-6.0)	0.0 (0.0-1.0)	3.5 (2.0-7.0)	0.0 (0.0-0.5)	5.0 (3.0-7.0)		0.0 (0.0-0.0)	.49	.94	.002		.56	.63
48 h	4.0 (2.0-6.5)	0.0 (0.0-1.0)	5.0 (3.0-7.0)	0.0 (0.0-0.5)	4.0 (2.0-5.0)		0.0 (0.0-0.0)	.31	.03	.19		.04	.66

Abbreviations: HIIT, high-intensity interval training; MICT, moderate-intensity continuous training; PCC, post-COVID condition; ST, strength training; VAS, visual analog scale.

^a^ For 30 patients with PCC and 31 controls.

^b^ Score range, 0 (no feeling) to 10 (worst possible feeling).

^c^ Differences in changes from baseline to immediately after exercise and to 48 hours after each exercise trial were compared between patients with PCC and control individuals following each exercise session.

^d^ Differences in changes immediately after exercise and 48 hours after exercise between the 3 exercise trials were compared in patients with PCC and controls separately.

**Figure 2.**
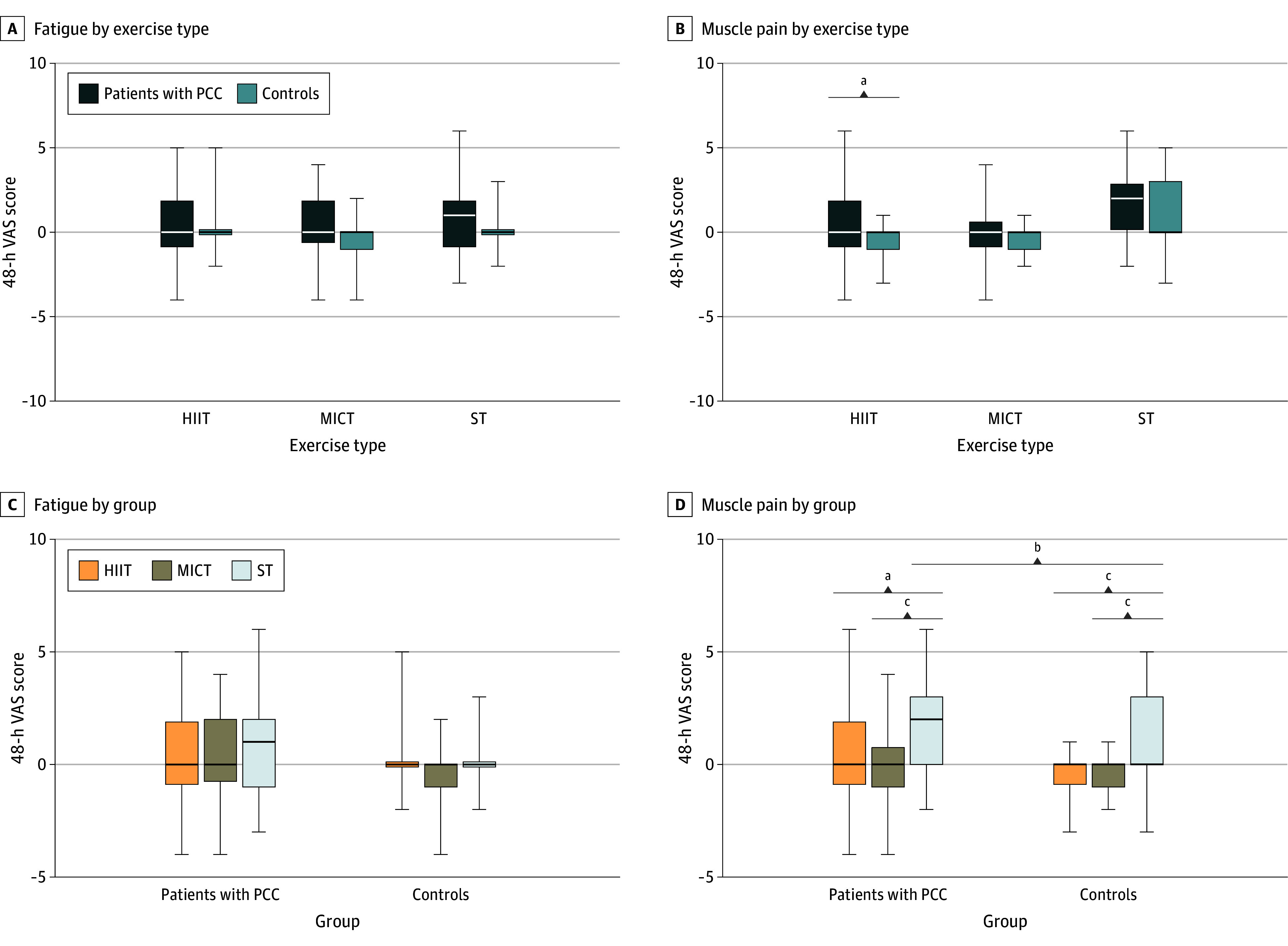
Fatigue and Muscle Pain Responses to 3 Different Exercise Sessions High-intensity interval training (HIIT), moderate-intensity continuous training (MICT), and strength training (ST) were conducted in a randomized crossover design in patients with post-COVID condition (PCC) (n = 30) and age- and sex-matched healthy controls (n = 31). Differences in changes from baseline to 48 hours following each exercise trial in the fatigue visual analog scale (VAS; score range, 0 [no feeling] to 10 [worst possible feeling]) (A) and the muscle pain VAS (B) were compared between patients with PCC and controls. Differences in changes 48 hours after exercise in the fatigue VAS score (C) and the muscle pain VAS score (D) among 3 exercise trials were compared in the group with PCC and the control group separately, and individual post hoc tests were used to compare exercise sessions when a main effect of exercise was found. Change in the muscle pain VAS score 48 hours after ST exercise was also compared between patients with PCC and controls. Horizontal bars indicate medians; lower and upper ends of the boxes, the first and third quartiles; and whiskers, minimum and maximum values. ^a^*P* < .05. ^b^*P* > .05 (not significant). ^c^*P* < .001.

There was no difference in fatigue worsening between patients with PCC and controls after any of the exercise types (mean [SD] VAS ranks for HIIT: PCC, 29.3 [19.5]; controls, 28.7 [11.4]; *P* = .08; MICT: PCC, 31.2 [17.0]; controls, 24.6 [11.7]; *P* = .09; ST: PCC, 31.0 [19.7]; controls, 28.1 [12.2]; *P* = .49) (Figure 2A). Patients with PCC reported worse muscle pain (mean [SD] VAS ranks, 33.4 [17.7] vs 25.0 [11.3]; *P* = .04) (Figure 2B) and joint pain (mean [SD] VAS ranks, 33.6 [12.3] vs 24.9 [11.0]; *P* = .009) (Table 2) after HIIT than the control group. After ST, there were no significant differences between the groups. Individual changes for fatigue and muscle pain are also presented in eFigures 1 and 2 in Supplement 2. At 48 hours after MICT, patients with PCC reported higher concentration impairments compared with controls (mean [SD] VAS ranks, 33.0 [17.1] vs 23.3 [10.6]; *P* = .03). In contrast, lymph discomfort was lower at 48 hours after MICT in patients with PCC compared with controls (mean [SD] VAS ranks, 25.5 [12.0] vs 31.6 [4.9]; *P* = .02).

To examine which exercise mode was better tolerated in terms of symptom exacerbation, differences in changes from baseline to 48 hours after exercise for each symptom were compared among the 3 sessions (HIIT, MICT, and ST) in each group separately. Patients with PCC reported no worsening of fatigue after any of the exercise types (Figure 2C). However, after ST compared with the other modes, patients with PCC reported a greater increase in muscle soreness (mean [SD] VAS ranks for HIIT vs ST, 20.9 [13.7] vs 32.1 [13.6]; *P* = .007; for MICT vs ST, 17.9 [11.7] vs 33.1 [12.4]; *P* < .001) (Figure 2D) and lymph node discomfort (eg, mean [SD] VAS rank after MICT vs ST, 20.8 [9.3] vs 30.2 [11.9]; *P* = .005) (Table 2). In contrast, MICT resulted in greater difficulty concentrating after 48 hours compared with ST (mean [SD] VAS ranks, 30.6 [13.0] vs 20.4 [13.7]; *P* = .01). Detailed results for VAS symptoms are provided in Table 2, while median group and exercise differences and IQRs are given in eTables 3 and 4 in Supplement 2, respectively. Results from the Multidimensional Fatigue Inventory, Profile of Mood States, and Somatic and Psychological Health Report questionnaires are provided in eTables 5 and 6 in Supplement 2.

### 48-Hour Postexercise CPET Results and CK and IL-6 Levels

**Figure 3.**
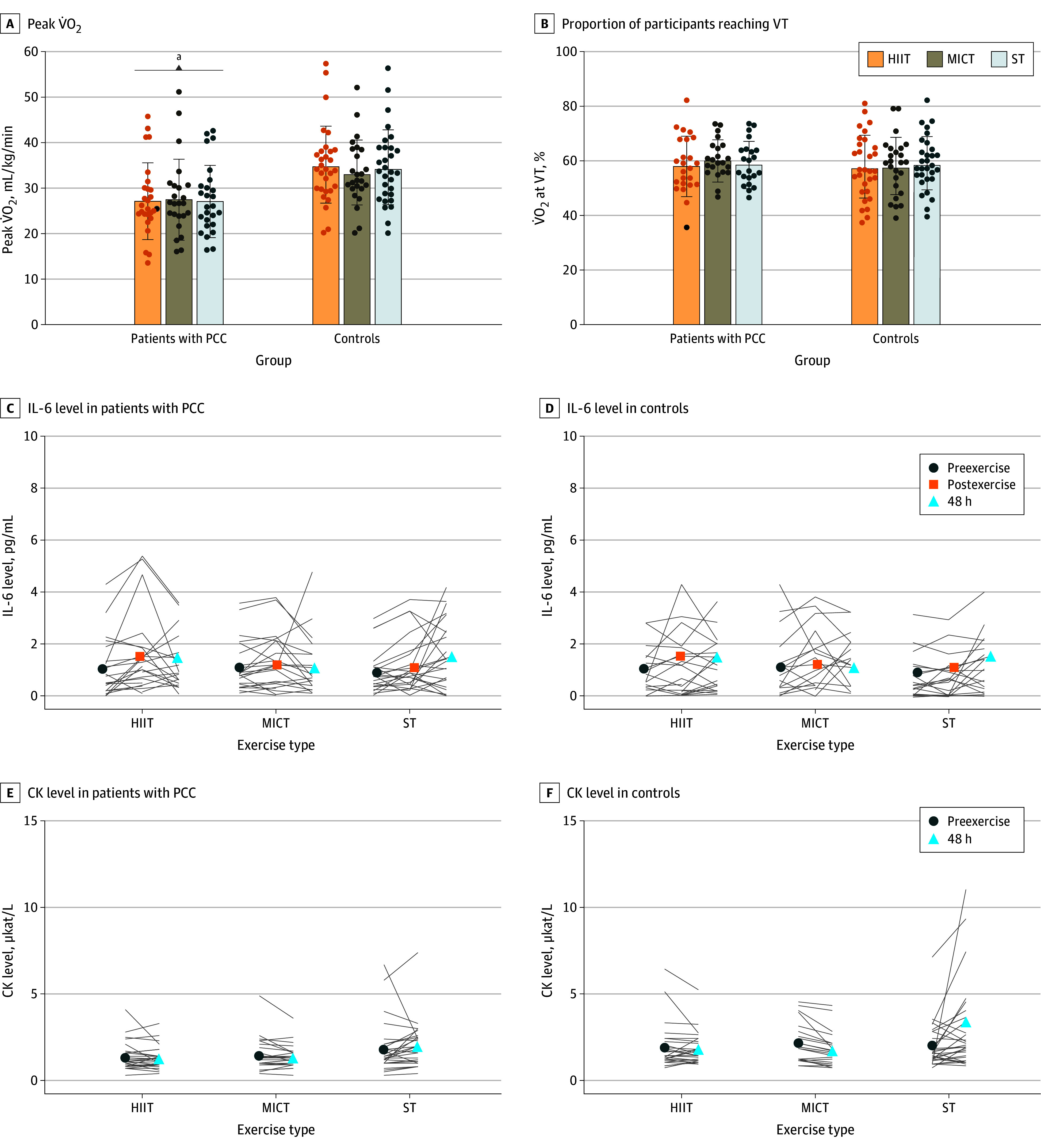
Aerobic Capacity and Inflammatory Responses to 3 Different Exercise Sessions A and B, Aerobic capacity, presented as the peak volume of oxygen consumption (V̇O_2_), and the percentage of individuals reaching ventilatory threshold (VT), both of which were assessed via cardiopulmonary exercise testing 48 hours after 3 exercise sessions (high-intensity interval training [HIIT], moderate-intensity continuous training [MICT], and strength training [ST]) in patients with post-COVID condition (PCC) (n = 30) and age- and sex-matched healthy controls (n = 31). C and D, For group, *P* = .37; exercise, *P* = .18; time, *P* = .02; group × exercise, *P* = .04; group × time, *P* = .49; exercise × time, *P* = .31; group × exercise × time, *P* = .92. E and F, For group, *P* = .06; exercise, *P* < .001; time, *P* = .27; group × exercise, *P* = .52; group × time, *P* = .26; exercise × time *P* = .004; group × exercise × time, *P* = .054. Bars represent means; whiskers, SDs; and dots, individual data. CK indicates creatine kinase; IL-6, interleukin 6. To convert CK to U/L, divide by 0.0167. ^a^*P* < .05.

There were no significant differences between groups or exercises in peak heart rate, RPE score, lactate concentration, or respiratory exchange ratio (eTables 7-9 in Supplement 2). At 48 hours after each exercise session, the group with PCC had 20% lower peak V̇O_2_ compared with controls independently of the preceding exercise type (mean difference, −6.2 mL/kg/min; 95% CI, −2.1 to −10.3 mL/kg/min; *P* = .004). The V̇O_2_ at the VT was also 16% lower and onset of blood lactate accumulation at 4 mmol/L was 20% lower in patients with PCC than in controls 48 hours after all exercise sessions, while relative percentages were comparable between groups and exercises (Figure 3A and B).

Levels of IL-6 and CK were not significantly different between the groups (Figure 3C and D and eTable 10 in Supplement 2). For IL-6 release, there was a main time effect, with higher values at 48 hours after exercise compared with before exercise in both groups (mean difference, 3.2 pg/mL; 95% CI, 0.1-0.5 pg/mL; *P* = .005) (Figure 3C and D). A main effect of exercise and an interaction effect (exercise × time, *P* = .004) indicated that ST resulted in a CK increase 48 hours after exercise compared with baseline and was similar in both groups (Figure 3E and F).

### Physiologic Function

Spirometry- and echocardiography-derived variables were within the normal range for all participants. However, patients with PCC had a 14% smaller stroke volume (mean difference, −2.6 mL; 95% CI, −4.9 to −0.2 mL; *P* = .02) and a 5% smaller left ventricular diameter (mean difference, −9.7 mm; 95% CI, −18.0 to −1.4 mm; *P* = .03) on echocardiography compared with control participants, with no differences in cardiac index or ventricular function (left ventricular ejection fraction, tricuspid annular plane systolic excursion). Arterial stiffness was also greater in the patients with PCC, with an 8.3% higher aortic pulse wave velocity (mean difference, 0.7 m/s; 95% CI, 0.0-1.4 m/s; *P* = .04). Full results are shown in Table 1 and mean group differences (95% CIs) in eTable 11 in Supplement 2.

Baseline peak V̇O_2_ was 21% lower in patients with PCC compared with controls (mean difference, −6.8 mL/kg/min; 95% CI, −10.7 to −2.9 mL/kg/min; *P* < .001). Maximal heart rate, lactate concentration, respiratory exchange ratio, and relative workload at the VT and at 4 mmol/L onset of blood lactate accumulation were similar between groups. The 6-minute walking distance did not differ between groups.

Isometric knee extension strength was 19% lower in the patients with PCC (mean difference, −37 Nm; 95% CI, −67 to −7 Nm; *P* = .02). However, knee extension isokinetic torque or handgrip strength did not differ between groups.

Blood and plasma volumes were slightly lower (blood by 7% and plasma by 8%) in patients with PCC but were not significantly different between the groups (blood volume, −5.2 mL/kg [95% CI, −11.8 to 1.3 mL/kg]; *P* = .12; plasma volume, −3.4 mL/kg [95% CI, −7.4 to 0.6 mL/kg]; *P* = .09). Blood status and biochemical blood markers, including C-reactive protein levels, were similar between the groups (Table 1). Head-up tilt testing revealed POTS in 2 patients with PCC (6%) and borderline POTS in 2 others (6%); no POTS was present in control participants.

Accelerometry revealed similar total physical activity and sedentary behavior. However, patients with PCC spent 43% less time in moderate to vigorous physical activity (mean difference, −26.5 minutes/d; 95% CI, −42.0 to −11.1 minutes/d; *P* = .001).

### Neurophysiologic Examination

Sensory and motor nerve conduction were within the normal range in all participants, with no differences between groups. Heart rate variability during normal breathing was comparable between groups. However, patients with PCC showed 19% less variability during deep breathing (mean difference, −5.7%; 95% CI, −11.3% to −0.1%; *P* = .05). The sympathetic skin response was normally elicited in all participants (Table 1). Electromyography was performed in 29 patients with PCC (94%) and 28 control participants (90%). Myopathic findings were observed in 18 patients with PCC (62%) (13 [45%] had myopathic findings in ≥2 muscles and 5 [17%] in 1 muscle) compared with only 1 control participant (4%), who had myopathic indices in 1 muscle. Neuropathic findings were observed in 1 patient with PCC (4%) and 1 control participant (4%) in 1 muscle each. Quantitative analysis of MUPs and group comparisons are shown in eTable 12 in Supplement 2.

## Discussion

This study investigated exercise intolerance in nonhospitalized, previously healthy patients with PCC compared with age- and sex-matched healthy controls. To elucidate possible mechanisms, we investigated the physiologic responses to acute exercise and performed a comprehensive physiologic characterization. The main finding was that participants with PCC generally tolerated all exercise sessions without significant worsening of symptoms or decline in aerobic performance after 48 hours. However, patients with PCC showed lower aerobic capacity and muscle strength compared with controls. We also observed a higher incidence of myopathic signs and indices of exaggerated orthostatic response in patients with PCC. While exacerbation of symptoms by exercise was not observed at the group level, our results indicate an underlying dysfunction in multiple organ systems that may have contributed to activity limitations in a subset of patients with PCC.

### Exercise Responses

Preexercise to postexercise changes were largely comparable between patients with PCC and controls regardless of the type of exercise (overall increase of approximately 0.5-1 unit on the 0-10 VAS). While this indicates no general symptom exacerbation after exercise, it should be noted that changes in fatigue, pain, and concentration from baseline to 48 hours after exercise varied among individuals (eFigures 1 and 2 in Supplement 2). Nonetheless, the observation that nonhospitalized patients with PCC could tolerate various physical activities without escalation of symptoms is important. It implies that physical activity tailored for patients on an individual basis could be an essential component in rehabilitation to enhance physical function and counteract muscle deconditioning. This is in line with recent reports supporting symptom-guided exercise rehabilitation in individuals with PCC.^31,32^

Notably, we observed no discrepancies between patients with PCC and control participants in exercise intensity corresponding to VT and onset of blood lactate accumulation at 4 mmol, indicating undisturbed metabolic exercise response at baseline and at the 48-hour follow-up. Previous suggestions that earlier lactate accumulation, possibly due to peripheral mitochondrial dysfunction leading to premature conversion to anaerobic glycolysis, occurs in the context of PCC and other postviral diseases have not been conclusively proven.^33,34^ Two reports found decreased VT^35^ and increased lactate levels^36^ after COVID-19, whereas other studies found no difference in VT between patients with PCC and control participants.^37,38^ Importantly, the former studies included patients with relevant comorbidities, hospitalizations, and occasionally intensive care or mechanical oxygen support,^35,36^ factors that are known to have adverse effects on peripheral muscle tissue.^39,40^

### Physiologic Function

Patients with PCC participated less in moderate to vigorous physical activity and showed lower aerobic capacity, as previously shown^1,9^ even in nonhospitalized patients.^38,41^ The maximum heart rate was consistent across groups, and all individuals in the cohort met the peak V̇O_2_ criteria (ie, reached >85% of their predicted maximum heart rate, respiratory exchange ratio >1.10, RPE >18, and lactate accumulation >8 mmol).^42^ However, even though within normal ranges, patients with PCC had reduced stroke volume and left ventricular diameter and, although not significant, approximately 7% lower blood volume compared with controls. It is plausible that this was due to the reduced levels of high- and moderate-intensity physical activity, which are associated with decreased blood volume and venous return affecting left ventricular diameter and stroke volume,^43^ and to increased resting heart rate, as observed in this study.

In addition to the higher resting heart rate, we also observed (with HUTT) that 13% of patients with PCC had an exaggerated heart rate response when standing, suggesting orthostatic intolerance. While deconditioning can partially explain the observed orthostatic intolerance (blood volume reduction can lead to a heart rate elevation), we cannot exclude the possibility that impaired autonomic function after viral infection^44,45^ (ie, higher levels of autoantibodies against α and β adrenoreceptors and muscarinic receptors)^46^ led to the exaggerated heart rate response. Patients with PCC also showed lower heart rate variability during deep breathing and increased foot latency as assessed in the sympathetic skin response test, suggesting parasympathetic nervous system involvement in a subgroup of patients with PCC.^47^ Finally, patients with PCC showed higher pulse wave velocity, indicating increased arterial stiffness compared with controls. This vascular involvement points to possible mechanisms, such as inactivity,^48^ direct viral damage, or cytokine-mediated effects on the vascular endothelium,^49^ which should be further investigated.

### Skeletal Muscle Indices

Of patients with PCC, 62% showed myopathic signs compared with a single case in the control group. Our EMG results suggested myogenic-derived rather than neurogenic causes of the myopathies in patients with PCC, evident in characteristics such as early recruitment of small, short-duration, frequent, polyphasic MUPs.^30^ Neurogenic disorders, typically indicated by signs of denervation, were not observed. The absence of peripheral nerve dysfunction in the nerve conduction assessments in conjunction with the presence of myopathic indices corroborates the predominantly myogenic nature of the EMG findings. This is consistent with other studies reporting changes of myogenic origin in patients with PCC,^10,50^ although a recent study pointed to structural muscle problems and exercise-induced myopathy.^38^ Collectively, these observations highlight muscular impairment in patients with PCC, which is further supported by the observed decrease in isometric muscle strength. Other factors, such as inflammation, capillary changes, and mitochondrial dysfunction, may also contribute to muscle impairments in patients with PCC and should be further investigated.^10,37,38^

Although baseline C-reactive protein and IL-6 levels in the cohort with PCC were normal, it is plausible that dysregulation of the immune system in the acute phase or persistent systemic inflammation could cause long-lasting impairments in muscles or neurons, which could explain the observed peripheral tissue problems as well as severe fatigue and myalgia.^8^ This phenomenon is not unique to COVID-19; persistent fatigue, cognitive problems, and mental difficulties have been observed for up to 4 years in patients after infections such as Middle East respiratory syndrome,^51^ severe acute respiratory syndrome,^52,53^ and Epstein-Barr virus.^54^ Viral infections have long been considered a major cause of myalgic encephalomyelitis–chronic fatigue syndrome.^55^ Although the exact mechanisms of postviral syndrome are unclear, viruses are thought to trigger aberrant immune responses that cause persistent mild inflammation and immune cell dysregulation, leading to long-lasting symptoms.^8^ We speculate that COVID-19 may affect muscle cells and autonomic neurons directly or via inflammatory pathways, causing myopathy and/or dysautonomia in some patients. This muscle damage may also contribute to decreased muscle strength, possibly leading to difficulty exercising and exacerbation of symptoms.

### Strengths and Limitations

A strength of the study was the comprehensive design including several clinical assessments and 3 different exercises, but the sample size was relatively small. We chose the 48-hour follow-up time point based on previous evidence in myalgic encephalomyelitis–chronic fatigue syndrome,^56^ but we do not know whether symptoms may have peaked after this period.

The cohort with PCC included previously healthy individuals with persistent symptoms 3 or more months after SARS-CoV-2 infection, without relevant comorbidities or hospitalization. We felt it was important to include healthy controls matched for age and sex, but it would also be relevant to compare patients with PCC with other patient groups experiencing long-term illnesses and/or exercise intolerance. Although patients with PCC in this study did not have severe effects, they reported marked symptoms at baseline, including fulfilling the criteria for PEM and persistent physical and mental fatigue that affected their health and well-being. Our results should not be generalized to all patients with PCC, and we are aware of the serious problems faced by many individuals.

Myopathic indices were found in 62% of patients with PCC, in contrast to 1 case in the control group. Although this result seems clear and robust, we acknowledge that the study was not able to determine whether these myogenic changes were already present prior to the infection. Further analyses are essential to clarify the origin of these myopathic indices.

## Conclusions

In this randomized crossover clinical trial, nonhospitalized patients with PCC generally tolerated all exercise types without reporting significant symptom exacerbation, performance reductions, or exacerbated inflammation after 48 hours. They had largely preserved respiratory and cardiovascular functions but showed lower aerobic capacity and muscle strength as well as signs of myopathy and orthostatic intolerance. It is plausible that our results represent a phenotype indicative of inactivity coupled with primary peripheral tissue damage and neurophysiologic changes leading to further difficulty in performing strenuous activity. However, given that exercise was generally well tolerated, guidelines cautioning against exercise in similar populations may need to be revised. It seems advisable to cautiously incorporate exercise into rehabilitation protocols and adjust the intensity progressively, considering patients’ symptoms and abilities.

## Notes

Supplement 1.
Trial Protocol

Supplement 2. **eTable 1.** Participant Demographics **eTable 2.** Responses During Exercise Sessions **eTable 3.** Median Group Differences in 10 Symptoms Assessed With Visual Analog Scale Symptoms **eTable 4.** Median Exercise Differences in 10 Symptoms Assessed With Visual Analog Scale Symptoms **eTable 5.** Results From Postexertional Symptoms as Assessed With MFI, POMS, and SPHERE Questionnaires **eTable 6.** Mean Group Differences in Postexertional Symptoms Assessed With MFI, POMS, and SPHERE Questionnaires **eTable 7.** Variables From Cardiopulmonary Exercise Testing at 48 Hours After Exercise Sessions **eTable 8.** Mean Group Differences in Variables From the Cardiopulmonary Exercise Testing at 48 Hours After Exercise Sessions **eTable 9.** Mean Exercise Differences in Variables From the Cardiopulmonary Exercise Testing at 48 Hours After Exercise Sessions **eTable 10.** CK and IL-6 in Response to 3 Exercise Sessions **eTable 11.** Mean Group Differences Baseline Characteristics **eTable 12.** Supplementary Results From the Examinations Included in the Physiological Characterization **eFigure 1.** Individual Changes for Fatigue as Assessed via Visual Analog Scale in Response to 3 Exercise Sessions **eFigure 2.** Individual Changes for Muscle Pain as Assessed via Visual Analog Scale in Response to 3 Exercise Sessions

Supplement 3.
Data Sharing Statement
