# Supplementary material for: Functional Limitations and Exercise Intolerance in Patients With Post-COVID Condition: A Randomized Crossover Clinical Trial
Source: JAMA Netw Open. 2024 Apr 4;7(4):e244386. doi: 10.1001/jamanetworkopen.2024.4386 (PMC11192186; doi:10.1001/jamanetworkopen.2024.4386)
Supplement: Supplement 1. — Trial Protocol [file jamanetwopen-e244386-s001.pdf]

Supplement 1

Clinical trial protocol (including detailed methods and statistical plan)

CONSORT Checklist (submitted for JAMA Network Open publication)

Clinical trial protocol

SYNOPSIS

**TITLE**	**EX**ercise **I**nto**LE**rance in post-COVID-19 patients (EXILE) - a randomized clinical trial
**RESPONSIBILITY**	**Tommy Lundberg, PhD**<br>Principal Investigator<br>Karolinska Institutet | Department of Laboratory Medicine | Division of Clinical Physiology | ANA Futura, plan 8 | 141 52 Stockholm<br><br>**Andrea Tryfonos, PhD**<br>Co-Principal Investigator | Study director<br>Karolinska Institutet | Department of Laboratory Medicine | Division of Clinical Physiology | ANA Futura, plan 8 | 141 52 Stockholm<br><br>**Thomas Gustafsson, MD, PhD**<br>Co-Principal Investigator | Study Chair<br>Karolinska Institutet | Department of Laboratory Medicine | Division of Clinical Physiology | ANA Futura, plan 8 | 141 52 Stockholm<br>Karolinska University Hospital | Clinical Physiology Unit | 141 57 Huddinge<br><br>**Helene Rundqvist, PhD**<br>Co-Principal Investigator<br>Karolinska Institutet | Department of Laboratory Medicine | Division of Clinical Physiology | ANA Futura, plan 8 | 141 52 Stockholm<br><br>**Kaveh Pourhamidi, MD, PhD**<br>Co-Principal Investigator | Study Chair

	Karolinska University Hospital | Neurophysiology Unit | 141 57 Huddinge
**STUDY CENTER**	Karolinska Institutet | Department of Laboratory Medicine | Division of Clinical Physiology<br>ANA Futura, plan 8 | 141 52 Stockholm<br><br>Karolinska University Hospital | Clinical Physiology Unit | 141 57 Huddinge<br><br>Karolinska University Hospital | Neurophysiology Unit | 141 57 Huddinge
**PRIMARY OBJECTIVE**	Post-exertional malaise (PEM) symptoms of post-COVID-19 subjects and age/sex matched controls in response to 3 different exercise trials.
**STUDY DESIGN**	Interventional, randomized, single-center, two-arm crossover study
**STUDY ARMS**	1) Post-COVID-19 syndrome patients<br>2) Age- and sex-matched healthy control subjects
**SAMPLE SIZE**	40 post-COVID-19 syndrome patients and 40 age- and sex-matched healthy controls
**INCLUSION CRITERIA**	1. Age range 18–64 years<br>2. None of the patients' complaints have been present before March 2020<br>3. Verified positive COVID-19 PCR test (for the post-COVID-19 group only)<br>4. Symptoms have persisted for at least 3 months after COVID-19 infection<br>5. Reported worsening of symptoms after exertion<br>6. No previous history of cardiovascular disease<br>7. No history of generalized anxiety disorder or somatic symptom disorder<br>8. Written informed consent

**EXCLUSION CRITERIA**	1. Age outside the range of 18–64 years<br>2. Patient's symptoms occurred before March 2020<br>3. No verified positive COVID-19 PCR test<br>4. Symptoms disappeared within 3 months of COVID-19 infection<br>5. Has not reported worsening of symptoms after exertion<br>6. Current or previous cardiovascular disease<br>7. Current or history of generalized anxiety disorder or somatic symptom disorder
**SCHEDULE**	Recruiting first participant: September 2022<br>Recruiting last participant: July 2023<br>Study planned completion: July 2024

BRIEF SUMMARY

The post-COVID-19 syndrome poses an unprecedented challenge to modern society, affecting millions of people worldwide. Persistent fatigue and exercise intolerance are among the most common complaints of these subjects. The mechanisms of exercise intolerance in post-COVID-19 subjects remain yet unknown, which make the rehabilitation efforts complex and challenging. The overall goals of this project are to: 1) improve physiological understanding of symptoms in this clinical condition, 2) elucidate plausible mechanisms to explain exercise intolerance/symptom exacerbation, and finally 3) provide knowledge that can be directly applied in the clinical setting to improve diagnosis, care, and individualized rehabilitation of subjects with post-COVID-19 syndrome. Post-COVID-19 subjects and age/sex matched healthy controls will undertake a comprehensive set of physiological and functional assessments, followed by 3 experimental visits (in a randomized order), where acute exercise responses will be assessed in either continuous moderate intensity aerobic exercise, high intensity interval exercise, or strength training. The same set of physiological assessments will also be performed after 1 year in both post-COVID-19 subjects and healthy-matched controls to better understand the time course of the syndrome.

ADMINISTRATIVE INFORMATION

1. TITLE

EXercise **I**nto**LE**rance in post-COVID-19 patients (EXILE) - a randomized clinical trial

2. TRIAL REGISTRATION

This study is registered in ClinicalTrials.gov NCT05445830 (2023-07-02).

3. PROTOCOL VERSION

Version 1.0 21-11-2022 REF: 2021-05758-01
Version 1.1 22-06-2022 REF: 2022-02999-02

The study started following the amendments in version 1.1 and subsequent approval by the Swedish Ethical Review Authority.

4. FUNDING

The project has received funding via Karolinska Institute from Hannover Re (100,000 SEK), Tornspiran Foundation (50,000 SEK), Lars Hierta Memorial Foundation (42,000 SEK), Magnus Bergvalls Foundation (100,000 SEK) and a grant from CIMED (region Stockholm) of 2,100,000 SEK.

5. ROLES AND RESPONSIBILITIES

5a. Key trial contacts

Tommy Lundberg, PhD

Principal Investigator

Karolinska Institutet | Department of Laboratory Medicine | Division of Clinical Physiology | ANA Futura, plan 8 | 141 52 Stockholm

Tommy.lundberg@ki.se

Andrea Tryfonos, PhD

Co-Principal Investigator | Study director

Karolinska Institutet | Department of Laboratory Medicine | Division of Clinical Physiology | ANA Futura, plan 8 | 141 52 Stockholm

Andrea.tryfonos@ki.se

Thomas Gustafsson, MD, PhD

Co-Principal Investigator | Study Chair

Karolinska Institutet | Department of Laboratory Medicine | Division of Clinical Physiology | ANA Futura, plan 8 | 141 52 Stockholm

Karolinska University Hospital | Clinical Physiology Unit | 141 57 Huddinge

Thomas.gustafsson@ki.se

Helene Rundqvist, PhD

Co-Principal Investigator

Karolinska Institutet | Department of Laboratory Medicine | Division of Clinical Physiology | ANA Futura, plan 8 | 141 52 Stockholm

Helene.rundqvist@ki.se

Kaveh Pourhamidi, MD, PhD

Co-Principal Investigator | Study Chair

Karolinska University Hospital | Neurophysiology Unit | 141 57 Huddinge

Kaveh.pourhamidi@ki.se

5b. Composition of the research team

The research team will consist of physicians, scientists, biomedical analysts, and students/research assistants. The research team will meet on a weekly basis to

provide ongoing monitoring of the study, including subject recruitment, data collection and analysis.

Tommy Lundberg, PhD (Principal investigator – expertise in exercise physiology)

Andrea Tryfonos, PhD (Co-investigator – project manager, coordinate data collection/analysis, writing manuscripts)

Helene Rundqvist, PhD (Co-investigator – expertise in exercise immunology)

Kaveh Pourchamidi, MD, PhD (Co-investigator – expertise in neurophysiological assessment)

Thomas Gustafsson, MD, PhD (Head of the division – oversee the study, responsible for muscle biopsy and clinical assessments)

Martin Engvall, MD (Physician – conducting/supervising clinical assessments, oversee subjects' recruitment)

Gustav Jörnåker (MSc student/research assistant – data collection/analysis)

Lisa Eriksson (Biomedical analyst – conducting clinical assessments)

5c. Trial sponsor

Karolinska Institutet | Department of Laboratory Medicine | Division of Clinical Physiology | ANA Futura, plan 8 | 141 52 Stockholm

5d. Collaborators/ other participating institutions

1. Karolinska University Hospital | Unit of Clinical Physiology | 141 57 Huddinge

2. Karolinska University Hospital | Department of Neurophysiology | 141 57 Huddinge

6. INTRODUCTION

6a. Background and rationale

Although the clinical consequences of COVID-19 are still largely unknown, physicians are observing persistent symptoms such as muscle weakness, headache, fatigue, shortness of breath, palpitations, and tachycardia, as well as neurological and cognitive disturbances, in an increasing number of patients.[1,2] While some reports suggest that 74-88% of hospitalized COVID-19 patients experience symptoms lasting longer than 50-80 days, including fatigue, shortness of breath, and limitations in physical functioning,[3] other reports across all cases of COVID-19 suggest that at least 2.3% of infected individuals suffer from COVID-19 symptoms for longer than 12 weeks.[2] This corresponds to tens of thousands of cases in Sweden alone.

In the past 18 months, we have conducted clinical physiology and neurophysiological examinations in patients with ongoing or previous COVID-19 disease. These examinations have included diagnostic methods including cardiac, pulmonary, and vascular function, but also neurophysiological examinations, including Postural-orthostatic tachycardia syndrome (POTS). The result of these examinations has not given a clear picture of the etiology of the symptoms, due to many reasons such as randomly selected patients, unclear basis for referral, and lack of reference values for this group of patients. Given the apparent variability of symptoms and signs of long COVID-19 that we experience in our clinical setting, there is value in comprehensively stratifying and characterizing patients based on their physiological function. This is important because of the clinical difficulties in distinguishing and recognizing the multifaceted symptoms and signs of long COVID-19, particularly in relation to muscle weakness and fatigue, but also to narrow the differential diagnosis. In addition, a better understanding of neuromuscular function in this patient group may lead to a more individualized rehabilitation program, considering neurophysiological limitations and disabilities.

Along with a better physiological understanding of this patient population, structured methods are needed to meet the demands of rehabilitation. In this context, a major challenge is that exercise intolerance is a common and significant problem for the majority of individuals in this patient group.[4] Indeed, the recent World Physiotherapy briefing paper,[5] stated that physical activity, including exercise, should be approached with caution and vigilance to ensure that rehabilitation programs are restorative and not worsening a person's symptoms both during and in the days following exertion. Inevitably, therefore, many primary care providers are reluctant to incorporate exercise into the rehabilitation program, which risks exacerbating physical deconditioning.

While the mechanisms of exercise intolerance in post-COVID-19 patients are largely unknown, plausible candidates include muscle deconditioning, orthostatic intolerance, dysautonomia, exercise hyperventilation, and exacerbated inflammation.[6] A recent study reported that several patients had significant exertional dyspnea 3 months after the onset of COVID-19 symptoms,[6] despite a relatively mild disease course. Exercise capacity was reduced in all patients, as most of them could not reach their predicted maximal workload and none of them reached their predicted VO_2max. An increased VE/VCO_2 ratio was observed in

most patients, suggesting that hyperventilation-induced hypocapnia after infection and prolonged inactivity may be responsible for a variety of extremely disabling symptoms,[6] POTS has also been mentioned as a possible physiological feature of long-COVID-19 patients.[7] However, research in this area is conflicting, and the original clinical definition of the syndrome has since been diluted by the inclusion of other symptoms, risking some overlap with patients in other disease categories.[8]

Another plausible mechanism relates to the immune system and cytokine profile. An adequate initial adaptive immune response to SARS-CoV2 is associated with asymptomatic or mild disease, whereas systemic inflammation is common in patients with severe disease. Persistent (up to 60 days) systemic inflammation is characterised by an Il-6 and TNF signature, accompanied by high numbers of effector CD4+ and CD8+ T-cells. Recently, it was observed that 60% of patients who reported post-exertional exacerbation of symptoms met the scoring thresholds used in people with myalgic encephalomyelitis/chronic fatigue syndrome (ME /CFS).[9] It is therefore interesting to note that patients with ME /CFS have significant correlations between measures of T-cell metabolism and plasma cytokine abundance that differ from the correlations observed in healthy controls.[10] These data suggest impaired systemic and T-cell metabolism, associated with persistent immune changes, which may be one of the underlying mechanisms behind the experience of fatigue and maladaptive to exercise response.

6b. Purpose and aims

The post-COVID-19 syndrome poses an unprecedented challenge to modern society, affecting millions of people worldwide. Because persistent fatigue and exercise intolerance are among the most common complaints of these patients, the rehabilitation efforts required will likely be both complex and challenging. The overall goals of this project are to 1) assess whether post-COVID-19 patients experience symptom exacerbation and/or physical limitations following exercise 2) improve physiological understanding of symptom and mechanisms behind exercise intolerance in this patient population, and finally 3) provide knowledge that can be directly applied in the clinical setting to improve diagnosis, care, and individualized rehabilitation of patients with post-COVID-19 syndrome.

To explore these goals, following comprehensive physiological assessment, we will describe, in a randomized cross-over trial, the acute physiologic response to various exercise regimens and relate these to post-exertional symptoms in patients with long-COVID-19. The acute exercise response will be closely monitored after three different exercise modalities: continuous moderate aerobic exercise, high-intensity interval training, and strength training. The comprehensive set of physiological assessments at baseline, after acute exercise, and at the one-year follow-up, will include COVID-19-related symptoms, post-exertional symptoms, neurophysiological function, circulatory function, respiratory/ventilatory function, maximal oxygen uptake, strength, physical function, blood status, and cytokine profiling.

We believe that more detailed physiological characterization, along with a better understanding of the plausible mechanisms underlined exercise-induced symptom

exacerbation and/or exercise incapacity, are necessary to introduce viable rehabilitation programs in this patient population. This knowledge could also help in decision making on which tests should be performed in the clinical setting to improve care, rule out differential diagnoses, and drive the design of future targeted and individualized rehabilitation interventions for this vulnerable patient population.

7. OBJECTIVES

7a. Primary objective and hypothesis

Post-exertional malaise (PEM) symptoms of post-COVID-19 subjects and age/sex matched controls in response to three different exercise trials. The primary outcome is the comparison of the change in fatigue rating using the visual analog scale (VAS) 0-10 before and 48h after each training trial between the post-COVID-19 group and the control group. We hypothesize that the post-COVID-19 group will suffer from persistent fatigue at 48h after exercise compared to baseline, whereas control group will return to the baseline level at this time point.

7b. Secondary objectives and hypothesis

Other PEM symptoms (muscle pain, joint pain, lymph nodes, concentration, memory, headache, fever, sore throat) will be assessed using VAS 0–10 before, after and 48h after 3 different types of exercise the post-COVID-19 patients and controls and compared between the groups. An additional 3 validated questionnaires are used: a) Multifactional Fatigue Inventory (MFI),[11] b) Profile of Mood States (POMS),[12] and c) Somatic and Psychological Health REport (SPHERE)[13] will be administrated to participants before, immediate-, and 48h post-exercise to assess PEM. We hypothesize that the post-COVID-19 group will suffer from persistent fatigue at 48h after exercise compared to baseline, whereas control group will return to the baseline level at this time point.

Cardiopulmonary exercise testing (CPET) will be also performed in post-COVID-19 subjects and age/sex matched controls at 48 hours post different exercise trials to assess and compared the exercise (in)capacity. Peak volume of oxygen consumption (VO_2peak), and other parameters, including ventilation and VE/VCO_2 ratio, O_2 saturation, heart rate, blood lactate, and rating of perceived exertion (RPE) using Borg scale will be also used to assess exercise capacity in all participants at 48 hours following each exercise trial. We hypothesize that post-COVID-19 group may have lower exercise capacity at 48h compared to controls.

Physiological (comprehensive) characterization post-COVID-19 subjects and comparison with age/sex matched controls will provide knowledge on the physiological phenotype of the post-COVID-19 group. Numerous clinical examinations will be used to assess the physiological function of post-COVID-19 patients and compared to age/sex matched controls. Specifically, echocardiography and arteriography will be used to assess cardiovascular function, spirometry to assess respiratory function, cardiopulmonary exercise testing and 6-min walk test to assess exercise capacity, handgrip and isokinetic dynameter to assess the muscular strength of upper and lower body respectively, CO-rebreathing method to assess blood and plasma volume, and head up tilt-test to assess dysautonomia. In addition, neurophysiological assessment includes nerve conduction studies, sympathetic skin response, heart rate variability studies, and needle EMG. Questionnaires, blood samples and muscle biopsy will be also collected and compared between the groups.

Finally, physiological function at 1y follow-up in post-COVID-19 patients and age/sex matched controls will be assessed. Same clinical investigations as the

baseline assessment will be performed at 1y follow-up in post-COVID-19 patients and age/sex matched controls. Comparisons within and between groups from baseline and 1y follow-up will be performed.

8. TRIAL DESIGN

This is a prospective interventional randomized two-arm crossover designed study. Two experimental groups: a) post-COVID-19 syndrome patients and b) age- and sex-matched healthy controls performed 3 different exercise sessions in a randomized counterbalanced order. Researchers will be blinded during the data analysis and during the data collection as far as possible.

9. METHODS: PARTICIPANTS, INTERVETIONS AND OUTCOMES

Thirty post-COVID-19 patients will be recruited through the post-COVID-19 clinic at Karolinska University Hospital Huddinge or other channels (advertisements posted on the Karolinska Institute website, social channels, information boards, and advertisements on the Swedish Post-COVID-19 patients' organization webpage and social channels. Interested participants contacted us for further information. Interested participants contacted us for further information Patients should be aged between 18–64 years, experienced persisting symptoms at least 3 months, including post-exertional exacerbation symptoms, and had no previous history of cardiovascular disease, general anxiety syndrome or somatic symptom disorder. In addition, 30 healthy control subjects, matched for sex, age and physical fitness will be also recruited from the community (advertisements posted on the Karolinska Institute website, social channels and information boards). All subjects will perform a comprehensive set of physiological assessments, including COVID-19-related symptoms, post-exertional symptoms, neurophysiological function, cardiovascular and circulatory function, respiratory/ventilatory function, maximal oxygen uptake, muscle strength, physical function, blood status, and cytokine profiling. Subjects will then complete 3 exercise trials in a randomized counterbalanced order: continuous moderate-intensity training (MICT), high-intensity interval training (HIIT), and strength training (ST). The acute exercise response will be thoroughly and continuously monitored, including measurements of O_2 saturation, blood pressure, ventilatory response, oxygen uptake, and blood lactate. In addition, blood samples will be collected before, immediately after, and 1 hour after each exercise session to describe leukocyte and cytokine release. After a 48-hour rest period following each exercise session, patients will return to the laboratory for symptom assessment, graded exercise testing, and blood sampling. Finally, after 1 year, we will perform a physiological follow-up using the same measurements as the baseline examination. Figure 1 shows the study design divided into two main parts, physiological assessment (A; comprehensive physiological characterization and 1 year follow-up, B; acute exercise sessions).

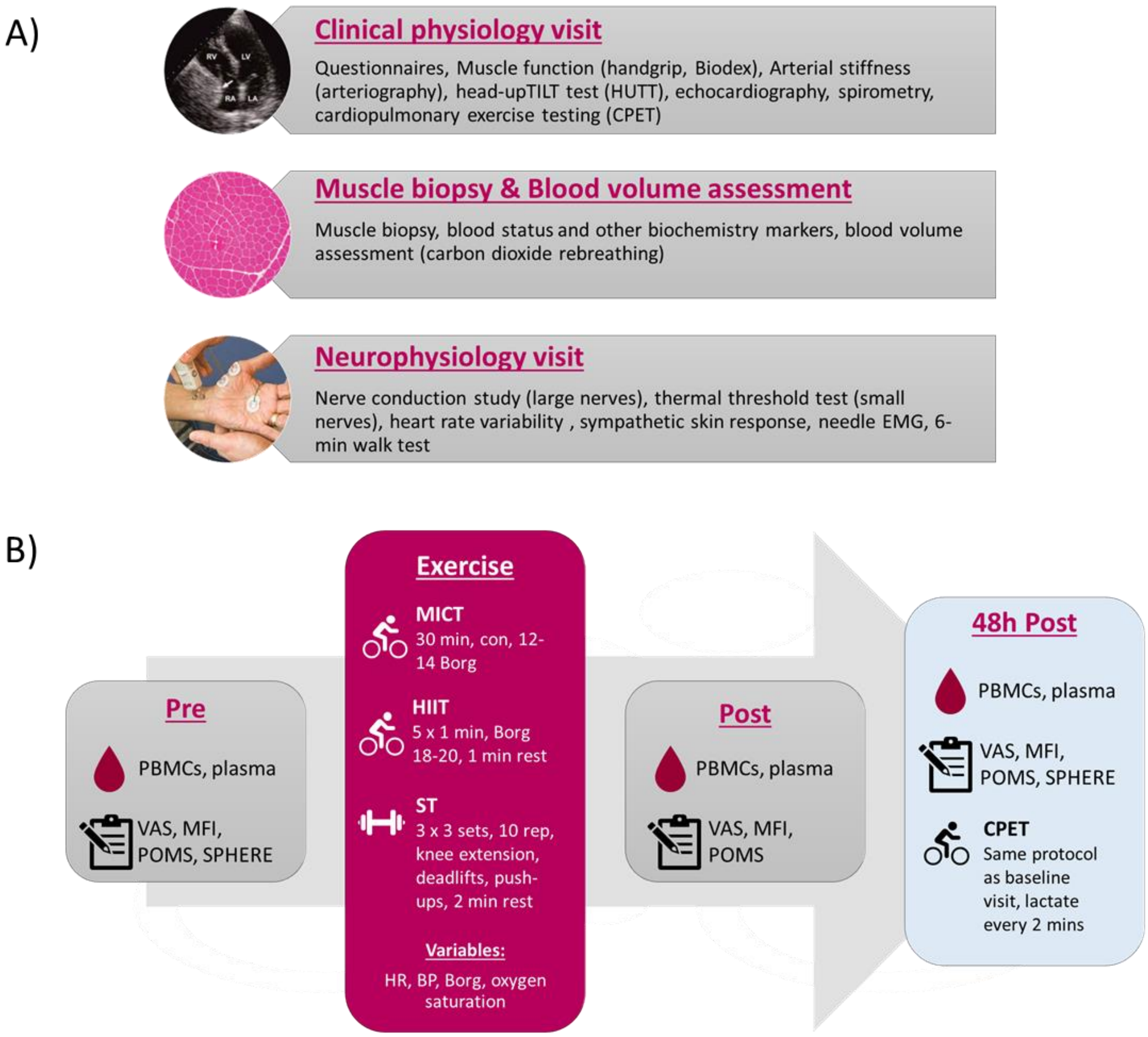

<u>Figure 1:</u> Study design, including physiological (characterization) assessments spread into 3 visits (A) and acute exercise sessions (B). Same assessments as in (A) will be conducted at 1-year follow up.

PBMCs: peripheral blood mononuclear cells; VAS: Visual analog scale; MFI: Multifactional Fatigue Inventory; POMS: Profile of Mood States; SPHERE: Somatic and Psychological Health Report; MICT: moderate-intensity continuous training, HIIT: high-intensity interval training, ST: strength training, CPET: cardiopulmonary exercise testing

10. ELIGIBILITY CRITERIA

Inclusion criteria

1. Age range 18–64 years
2. None of the patients' complaints have been present before March 2020
3. Verified positive COVID-19 PCR test (for the post-COVID-19 group only)
4. Symptoms have persisted for at least 3 months after COVID-19 infection

5. Reported worsening of symptoms after exertion
6. No previous history of cardiovascular disease
7. No history of generalized anxiety disorder or somatic symptom disorder
8. Written informed consent

Exclusion criteria

1. Age outside the range of 18–64 years
2. Patient's symptoms occurred before March 2020
3. No verified positive COVID-19 PCR test
4. Symptoms disappeared within 3 months of COVID-19 infection
5. Has not reported worsening of symptoms after exertion
6. Current or previous cardiovascular disease
7. Current or history of generalized anxiety disorder or somatic symptom disorder

11. INTERVATIONS

11a. Description of interventions for each group

<u>Exercise response</u>

Subjects will perform high-intensity-interval training (HIIT), moderate intensity continuous exercise (MICT) and strength training (ST). The acute exercise responses will be closely monitored, including measurements of O_2 saturation, heart rate, blood pressure, rating of perceived exertion (Borg scale 6–20) and blood lactate. In addition, symptoms via multiple validated questionnaires and blood samples will be collected before, immediately after each exercise session to describe leukocyte and cytokine release. After a 48-hour rest period subjects will return to the laboratory for symptom assessment, submaximal exercise response (CPET), and blood sampling.

<u>Physiological assessment (characterization)</u>

Subjects will perform a series of baseline assessment including neurophysiological function, circulatory and vascular function, blood volume assessment, respiratory/ventilatory function, maximal oxygen uptake, strength, physical function and fitness, blood status and muscle biopsy.

<u>1 year follow-up</u>

Subjects will perform the same series of baseline assessment in 1-year time, including neurophysiological function, circulatory and vascular function, blood volume assessment, respiratory/ventilatory function, maximal oxygen uptake, strength, physical function and fitness, blood status and muscle biopsy.

11b. Criteria for discontinuation or modification of interventions

All subjects will be informed verbally and in writing (consent form) that they have the right to discontinue the study at any time, without this affecting their standard medical care.

Researchers will be in contact with participants during their participation to ensure that their condition has not worsened due to or during the study participation. The research team will meet on a weekly basis and discuss such matters with the responsible physician.

12. EXPLANATIOS OF THE ASSESSEMETNS – OUTCOMES

Exercise trials (HIIT, MICT, ST)

Subjects will perform 5 minutes warm-up, then either of the following exercise training protocols in a randomized order, followed by 5 minutes of active recovery. **MICT:** Continuous 30 min cycling at moderate intensity **HIIT:** 5 x 1 min cycling at high intensity, 1 min rest **ST:** 3 sets x 10 repetitions of 3 exercises covering large muscle groups of the lower and upper body (2 min recovery between sets). Exercise sessions will be thoroughly and continuously monitored, including measurements of O_2 saturation, blood pressure, and blood lactate. In addition, blood samples will be collected before, immediately after, 1h and 48h post each exercise session to describe leukocyte and cytokine release. At 48 hours follow up, post-exercise symptoms and exercise responses to a monitored graded exhaustion test will also be reported.

HIIT session: 5 x 1-min intervals on a cycle ergometer (Monark LC6, Vansbro, Sweden) with a workload equal to 90% of the workload the patients achieve during the initial cardiopulmonary exercise testing (CPET) at baseline. Each interval will be interrupted by a 1-minute rest period in a seated position. Intensity is increased to be ≥16 on the Borg scale at the end of the session. The workload is increased if the perceived exertion is lower and decreased if subjects are unable to achieve an RPM above 55.

MICT session: 30 minutes of continuous cycling (Monark LC6, Vansbro, Sweden). Participants will be asked to cycle at 50% of the workload achieved during the baseline CPET. The intensity aims to correspond to 12–14 according to the Borg scale. If the rating is lower, the workload is increased and vice versa if the rating is higher. The monitoring of RPE is done every five minutes during the training session.

ST session: This session consists of 3 different exercises designed to train the entire body in the following order a) deadlifts, b) push-ups and c) knee-extensions. All three exercises will be conducted in three sets, with three minutes of rest between each set and/or exercises. The session will begin with three sets of 10 repetitions of deadlifts on a flywheel device (Kinetic Box (Kbox), Exxentric AB TM, Bromma, Sweden), equipped with one 4.6-kg flywheel with a moment of inertia of 0.07 $kg \cdot m^{-2}$. Participants will be instructed verbally and visually by the researcher and will familiarize themselves with six repetitions as a warm-up before starting the exercise sets. Participants will be asked to pull as hard as they can during the sets and will be verbally encouraged by the researcher. The second exercise will be

push-ups, performed in three sets. The goal for the push-ups will be to complete 10–15 repetitions in each set. Participants can choose to perform the push-up as a normal push-up (feet and arms on the floor), a push-up with knees on the floor, or with knees on the floor and arms raised 25 cm. They will also be instructed to leave one repetition in reserve to get reasonably close in repetitions in the following sets. Finally, the participants will perform three sets of 10 repetitions of knee extensions on a flywheel knee extension machine (YoYo Technology Inc., Stockholm, Sweden) equipped with an inertia that, according to the manufacturer's instructions, corresponds to 0.054 $kg \cdot m^{-2}$. Participants will sit and perform the exercise from ~80° knee joint angle to ~175° with full engagement throughout the sets, with encouragement from research staff. All three exercises will have three minutes of rest between sets to ensure adequate rest, and blood pressure (BP), heart rate (HR), oxygen saturation (SaO_2), and Borg scale ratings will be monitored after each exercise.

COVID-19-related symptoms and post-exertional symptoms

Post-exertional symptoms and general health of subjects will be captured through validated questionaries at baseline and 1-year follow up (SF-36, mMRC dyspea scale, DSQ-PEM).

In addition, subjects will report PEM symptoms before, immediately and 48h following acute exercise sessions using the following tools: VAS 0–10 for 10 symptoms (fatigue, muscle pain, joint pain, lymph nodes, concentration, memory, headache, fever, sore throat), Multifactional Fatigue Inventory (MFI),[11] Profile of Mood States (POMS)[12]. Finally, Somatic and Psychological Health REport (SPHERE) will be administered before and 48h after exercise trials.

Physical function and muscle strength and accelerometers

Physical function will be assessed via the validated 6-minute walk test. Handgrip strength will be measured via a hand dynamometer (maximal voluntary contraction), whereas isometric and dynamic knee extensor muscle function through isokinetic dynamometry (Biodex). Accelerometers will be given to participants to assess physical activity.

The 6-minute walk test: Subjects will be asked to walk for 6 minutes as fast as they can in a pre-determined area of 30 meters length according to standard American Thoracic Society guidelines [14]. Distance in meters will be used to assess their physical fitness.

Handgrip strength: The gold standard Jama hydraulic hand dynamometer (Model 5030 J1, Sammons Preston Rolyan, Bolingbrook, IL, USA) will be used to measure handgrip strength. Subjects will be instructed to hold the dynamometer in the dominant hand, extend the legs and arm, and hold the wrist in a neutral position, then squeeze the dynamometer as hard as possible for 4–5 seconds. The participants will have three attempts with a 1.5-minute rest in between, and the peak value from the test will be scored as the maximum grip strength.

Biodex: Maximal isometric and isokinetic strength (60°/s and 210°/s) will be measured using isokinetic dynamometry with a sampling frequency of 2000 Hz

(Biodex System 4 Pro, Biodex Medical Systems, NY). The chest, hip and thigh will be stabilized to the dynamometer using straps, and the ankle will be strapped to the lever arm, which will be aligned with the axis of rotation of the knee joint. The subjects will be allowed 2 attempts at each velocity (30 s rest) and the best result will be accepted as peak torque. Maximal isometric torque will be measured at knee angle 120° (60° from full extension).

Accelerometers: Physical activity will be assessed by accelerometers (GT3X Actigraph, USA). Participants will be asked to wear accelerometers for 7 days during the waking hours. Raw data are integrated into 60s epochs using ActiLife software and expressed as counts per minutes (cpm). Wear time will be estimated using the algorithm described Choi et al. 2011.[15] Patients with an estimated wear time of <4 days, <10h/day will be eliminated from further analysis.

<u>Cardiovascular/circulatory/respiratory function and work capacity</u>

Echocardiography, head-up TILT test and spirometry will be assessed according to established clinical procedures. Cardiopulmonary exercise test (CPET) to assess VO_{2peak} will be determined with an incremental cycling test to volitional fatigue on an electronically braked cycle ergometer using an online gas collection system. Arterial stiffness will also be captured through arteriography. Blood volume will be assessed using the CO-rebreathing method.

Echocardiography: A licensed biomedical scientist will conduct the echocardiographic examination using the Vivid E9 ultrasound system by General Electric in Horten, Norway with a 4V2c phased-array transducer. The subjects were positioned in the left lateral recumbent posture following a 5-minute supine rest, adhering to the clinical protocol derived from guidelines by the American Society of Echocardiography and the European Association of Cardiovascular Imaging. Subsequently, another blinded observer will quantify cardiac chamber dimensions and volumes, as well as the assessment of left ventricular systolic and diastolic function.

Head-up TILT test (HUTT): The examination will be performed according to clinical guidelines.[16,17] Prior to the test session, participants will be instructed to abstain from medications that can alter heart rate and blood pressure for 48h, as well as alcohol for 24h. They will be also instructed not to consume large meals 2–3 hours prior to the examination, including coffee consumption. The test entailed continuous HR registration using a 5-lead electrocardiogram (cable by Advantage Medical Cables, Inc) and continuous blood pressure registration using a finger-cuff that is been calibrated according to blood pressure measured using a brachial-cuff. The hemodynamic monitoring system will be used was Finapres NOVA® (Finapres Medical Systems, Amsterdam, Netherlands). Examination will be commenced with 10-min in supine position in order to obtain baseline measurements. Following this, the HUTT is applied at 70◦ for 20-min, to simulate standing conditions.[16,17] The analysis of hemodynamic data will be performed using NOVA Scope clinical software according to the most recent practical guidelines (2019) set forth by previous studies. [18] The analysis will be involved a three-step process: a) data preprocessing and filtering, b) feature extraction and c) determining clinical outcomes. The primary hemodynamic parameters to be used for analysis will be the beat-to-beat systolic blood pressure (SBP), diastolic blood pressure (DPB) and

heart rate (HR). Data preprocessing and filtering includes standing time determination and extraction, data quality assessment and rejection of artifacts such as motion artifacts, calibration artifacts or misidentification of beats. Feature extraction begins with baseline values, extracted as the mean of values acquired 60–30s before standing. Peak values will be extracted within the first 10-mins of tilt, as an average of 30s. The final step of the analysis process will include determining clinical classification, according to the recent consensus statement of the European Federation of Autonomic Societies.[16,17] The classifications will include normal orthostatic reaction, POTS, Syncope and orthostatic hypotension. POTS is characterized by development of symptoms with a sustained heart rate increase of ≥ 30 bpm or ≥ 120 bpm within 10 minutes of standing, with no significant blood pressure difference, i.e., SBP decreased by ≥ 20mmHg and DBP ≥ 10mmHg. For this study, borderline POTS is characterized by development of symptoms with a sustained heart rate increase of ≥ 27-29 bpm within 10 minutes of standing, with no significant blood pressure difference, i.e., SBP decreased by ≥ 20mmHg and DBP ≥ 10mmHg. Syncope is characterized by a significant fall of HR or BP with loss of consciousness or inability to maintain posture. And finally, orthostatic hypotension is characterized by any HR increase with SBP decrease of ≥ 20mmHG or DBP decrease of ≥ 10mmHG.

Spirometry: The procedure will be performed by a biomedical analyst using Jaeger MasterScreen PFT powered by SentrySuite, Hoechberg, Germany, according to hospital clinical guidelines following an established protocol. [19] Participants will be seated comfortably, with a nose clip in place to ensure that breathing occurs exclusively through the mouth. The procedure will begin with a maximal inhalation, followed by a forceful and sustained exhalation into the spirometer. Participants will be encouraged to exhale with maximal effort until their lungs are completely emptied. Multiple trials may be conducted to ensure the accuracy and consistency of the results. The spirometry variables will be the forced vital capacity (FVC), forced expiratory volume in the first second (FEV_1), FEV% (FEV_1/ VC MAX), maximum vital capacity (VC MAX), total lung capacity (TLC), and diffusing capacity of the lungs for carbon monoxide single breath corrected for Hb (DLCOc SB).

Cardiopulmonary exercise testing (CPET): A CPET will be performed to assess cardiorespiratory fitness, monitoring with an ECG to rule out potential risks of the exercise sessions in the study and subsequent CPETs. The CPET will be conducted on an RE990 electronically braked cycle ergometer (Rodby Innovation AB, Hagby, Sweden) with an online gas collection system (Jaeger®, Vyntus CPX, CareFusion, Hoechberg, Germany) to analyze exhaled gases using the breath-by-breath technique, wearing a Hans Rudolph mask. The test will begin with a three-minute rest period during which heart rate (HR), blood saturation (SaO_2), blood pressure (BP), and blood lactate accumulation (bLa) will be determined. After resting, participants will begin cycling for 3 minutes without resistance at a cadence of 60 revolutions per minute (rpm). After 3 minutes, resistance will be increased to 20 watts and then gradually increased by 10, 15, 20, or 25 watts per minute, with the goal of reaching maximal effort and VO_2peak in 8–12 minutes after voluntary fatigue.[20] Throughout the test, HR will be monitored by continuous 12-lead ECG recording, BP, and perceived exertion will be recorded every other minute of the test, and bLa concentration will be measured pre and 3 minutes after the test in a capillary sample from the earlobe using a Lactate Scout+ (SensLab GmbH, Leipzig,

Germany). The test will be considered maximal (VO_2peak) when two or more of the following criteria are reached: 90% of age-predicted HR (220-age), a respiratory exchange ratio (RER) ≥1.1, a peak bLa of ≥ 6 mmol·l-l, or a perceived exertion ≥18.[21] VO_{2peak} will be determined as the highest 20-second average. Subjects will be verbally encouraged during the test to increase their motivation for maximal exertion.

Arteriography: Measurements of aortic pulse wave velocity (PWV_{ao}) will be performed using non-invasive technique (Arteriograph, TensioMed Ltd., Budapest, Hungary). Participants will be rested in supine position for at least 10 minutes, and then a single upper arm cuff will be placed to measure PWV_{ao}. The device first measures the brachial blood pressure and then inflates the cuff to suprasystolic pressure to occlude the brachial artery. During this period, the cuff collects pure pressure signals and detects direct and reflected systolic wave peaks. The time difference between the early and late systolic peaks is equal to the time of the aortic pulse wave traveling down to the aortic bifurcation and back towards the heart. The aortic root-bifurcation transit time can be calculated, and by estimate the straight distance between the suprasternal notch and pubic bone (an acceptable estimate of the aortic length), the PWV_{ao} will be calculated by the software.[22]

Blood volume: The optimized carbon monoxide rebreathing method will be used to determine tHb, from which BV and PV later were calculated using hemoglobin concentration [Hb] and hematocrit (Hct).[23] Subjects will rest for 15 minutes before a venous blood sample will be taken from the median cubital vein. The sample will be immediately analyzed for baseline carboxyhemoglobin (%HbCO), [Hb] and Hct. End-tidal carbon monoxide (CO) will be measured at baseline and after rebreathing using a CO gas analyzer (Dräger, PAC 700, Lübeck, Germany and Honeywell, BW Solo, Charlotte, NC, USA). Throughout rebreathing, the same gas analyzer will be used to check for CO leaks. Subjects will breathe a gas mixture of chemically pure (99.97%) CO (0.8 mL x kg^{-1}) and medical oxygen (AGA, Stockholm, Sweden) for 2 minutes before disconnecting from the spirometer (Blood tec GmbH, Bayreuth, Germany). Two venous blood samples (1 mL) will be taken, one before rebreathing and one 7 minutes after administration of CO. The samples will be then analyzed in duplicate for %HbCO (ABL 90 Flex, Radiometer A/S, Copenhagen, Denmark), tHb will be calculated as previously described.[24,25]

Neurophysiological function

Large nerve function is assessed by electroneurography of motor and sensory nerves in the upper and lower extremities. Small nerve fiber function is assessed by a standardized quantitative sensory test including a thermal threshold test in the feet and hands. Autonomic nerve function will be tested using sympathetic skin response (SSR) and heart rate variability (HRV) testing. Needle EMG is performed in proximal and distal muscles to assess the integrity of motor units; the presence of denervation potentials, neurogenic and myogenic motor units are quantified and recorded.

Nerve conductions studies (NCS): All participants will have a routine NCS at the same clinical neurophysiology laboratory on the dominant lower and upper limb using the Cadwell Sierra Summit EMG system (Cadwell Industries, Inc., Kennewick, WA). Skin temperature was maintained over 32 °C. Filters will be set

at 20 Hz–10 kHz for motor studies and 20 Hz–3 kHz for sensory studies. Motor studies included the median, ulnar, fibular, and tibial nerves, and the compound muscle action potential (CMAP), distal latency (DL), conduction velocity (CV), and minimum F-wave latency will be recorded for all nerves. Sensory studies will include median, ulnar, radial, sural, and fibular nerves, and the sensory nerve action potential (SNAP) and CV will be recorded for all nerves.

Electromyography (EMG): Needle-EMG of deltoid, biceps brachii, trapezius, anterior tibial, vastus medialis, and adductor magnus muscles will be performed in all participants using a concentric 35 mm needle electrode (26 G) with filter settings of 20 Hz–10 kHz. The EMG will be performed by 1–2 skin insertions per muscle at 5–10 different muscle sites separated by at least 2 mm. Quantitative EMG (qEMG) analysis will be done by sampling at least 20 motor unit potentials (MUPs) during weak voluntary contraction; gain (200 uV/division) and sweep speed (5-10 ms/division). Multi-MUP analysis and routine EMG will be performed to record and assess MUPs qualitative and semi-quantitatively. Mean duration (simple MUPs), amplitude, and percentage of polyphasic MUPs will be recorded. The presence of spontaneous activity and the interference pattern (IP) will be assessed on all tested muscles. Physician will then assess the EMG results qualitatively by visually observing clear overrepresentation of MUPs with myopathic morphology (if more than 50% of the saved MUPs data exhibited myopathic changes (short durations, small, polyphasic) together with supportive measures from the qEMG, particularly polyphasia, to classify a muscle as myopathic or not. Then the final impression/conclusion from the EMG test was classified as definitively myopathic if seen in at least 2 muscles, borderline if seen in one, neurogenic, mixed, or normal EMG findings. The Sierra Summit EMG system (Cadwell Laboratories Inc., Kennewick, WA, USA) was used for all measurements.

Sympathetic skin response (SSR): A standard surface electrode will be attached to the dominant palm and sole and the reference electrode to the dorsum of the hand and foot. Single electrical stimulus at the contralateral wrist (median nerve) of 20-30 mA intensity and 0.2 ms duration will be delivered. The stimulus will be delivered unexpectedly to avoid habituation. The response will be recorded, and the latency determined from the onset of the stimulus artifact to first deflection from baseline. A response will be defined as absent if no reproducible deflection is recorded after consecutive and sporadic stimulations. The Sierra Summit EMG system (Cadwell Laboratories Inc., Kennewick, WA, USA) will be used for all measurements.

Heart rate variability (HRV): Continuous electrocardiogram (ECG) will be recorded by a two-lead electrocardiograph using the Sierra Summit EMG system (Cadwell Laboratories Inc., Kennewick, WA, USA). The variation in R-R interval during normal breathing and during deep breathing will be recorded and the variability calculated as maximum heart rate minus minimum heart rate divided by average heart rate, expressed as a percentage.

<u>Blood sampling and muscle biopsies</u>

Routine haemoglobin concentration, glucose, lactate, triglyceride and cholesterol levels, leukocyte count, transferrin saturation, and CRP will be determined. Absolute immune cell counts will be determined using BD TruCount, peripheral blood mononuclear cells (PBMCs) will be analysed for distribution of B cells, NK

cells, monocytes, CD4+ T cells, CD8+ T cells, with additional panels to capture plasma, effector and memory cells. Exploratory metabolomic and cytokine profiling will be performed using the Olink PEA platform and GC-MS at the Swedish Metabolomics Center in Umeå, targeted analysis will include IL-6, TNFa and IFNg profiling. Immune results will be correlated with physiological outcomes and exercise response. Muscle biopsy will be also taken from the vastus lateralis muscle under local anesthesia using the Bergström needle technique. Since post-COVID-19 syndrome is associated with mitochondrial fragmentation and reduced levels of metabolites consistent with a hypometabolic syndrome, we will perform these measurements along with histochemical analyzes of fiber size, fiber type, myonuclear content, and gene/protein expression of markers involved in energy processes, muscle atrophy, endothelial dys(function), angiogenesis and mitochondrial biogenesis.

Muscle biopsy: Tissue samples of the vastus lateralis muscle will be obtained under local anaesthesia using the percutaneous Bergström technique from the right leg, after an overnight fast and with no prior exercise during the past 48h. All muscle samples (~200 mg) will be immediately dissected free of any visible fat, excess blood and connective tissue, and then quickly frozen in liquid nitrogen. Samples were stored at -80°C until the analyses.

13. PARTICIPANT TIMELINE

The study will start in September 2022.

Data analysis of the primary outcome and first manuscript is expected to be submitted by December 2023.

Further blood and tissue analysis from the physiological characterization and exercise sessions (i.e., muscle biopsies, blood sample cytokine profile, PBMCs) is estimated to be completed by September 2024.

The 1-year follow-ups are expected to finish during Summer 2024 and the study completion is estimated to be completed in December 2024, including data analysis and manuscripts.

Participant timeline, including details of the visits/interventions is shown in Figure 1. Briefly, we estimated that participants will be enrolled in the study for approximately 1 year from the first baseline visit to the last follow-up visit.

14. SAMPLE SIZE

Determining an appropriate sample size for this study was challenging as the post-COVID-19 syndrome was new and there was no established data on the expected effect size of the interventions and the consistency of patient responses. Nevertheless, based on clinical observations from previous studies, we expected a markedly different response to exercise in patients compared to controls. Indeed, symptom variability after acute exercise in myalgic encephalomyelitis/chronic fatigue syndrome (ME/CFS) shows moderate to large effect sizes for most symptoms assessed with VAS scales.[26] Our a priori estimate (using G*Power) was that we would need at least 28 patients and 28 controls to detect a moderate effect size (Critical F 0.25) between groups in a within-between interaction design with a power of 80% and an alpha of 5%.

15. RECRUITMENT

Patient inclusion is ensured through collaboration with the post-COVID-19 clinic and coordinator for research on this patient group established at Karolinska University Hospital. Patients will be informed about the current study and the opportunity to participate. Other social channels, including patients' post-COVID-19 association in Sweden will be sought if needed.

16. ALLOCATION

The research team will randomly allocate the order of exercise interventions. Same allocation will be applied in both groups. Research Electronic Data Capture (Redcap) software will be used for the random allocation. In addition, exercise order will be recorded and incorporated into the statistical model.

17. BLINDING

Researchers will be blinded as far as possible during data collection, in terms of group (post-COVID-19 vs. control). For example, the study personnel who conduct the clinical tests and/or exercise trials (physicians, biomedical analyst, research assistants etc.) will not be aware of the group allocation (Patient or Control). However, in some cases given the symptoms and/or physical conditions of subjects, this might be directly or indirectly revealed by the patient. Importantly though, researchers will be fully blinded during data analysis. Following informed consent, all subjects will receive a study ID and all the measurements will be stored according to this study ID. As such, data analysis will be blinded between and within groups.

18. DATA COLLECTION

At baseline and at 1-year follow-up, all physiological variables will be assessed under strictly standardized procedures in a laboratory setting. Physiological functions of the patients with post-COVID-19 condition will be compared with a well-matched healthy control group in an exploratory manner, as this patient population has yet to be characterized. Measurements before and after 1 year will be compared to determine if improvements are more related to certain physiological factors than others. Figure 1 and section 12 provide details in regards to the data collection for each particular outcome.

19. DATA MANAGEMENT

Logbook of all visits and test results will be kept. These are marked only with the research subjects' unique study number. Clinical examinations are entered into the patient record (TakeCare) where it is clearly stated that the tests were performed for research purposes.

All data is saved electronically via RedCap, which is an authentication protected and approved system for research documentation at Karolinska Institutet. All data that is collected is saved with the study ID of the research subjects as a label (sequential number). Names and personal details are not disclosed. Only involved researchers have access to data material. The code list to identify the research subjects is saved as a password-protected document with a paper copy saved in a study folder locked with the responsible researcher. The responsible researcher is responsible for all documents being archived in accordance with KI's rules for research documentation and archiving of research data (saved for at least 10 years).

20. STATISTICAL METHODS

Analyses will be performed using IBM SPSS Statistics for Windows, version 29.0 (Armonk, NY: IBM Corp). To assess the primary outcome (post-exertional symptom exacerbation from baseline to 48h after each exercise), a mix-linear model was initially planned to analyse the differences in symptom changes following three exercise sessions (HIIT, MICT, ST), between the two groups (post-COVID-19 patients vs. control). Subject ID and the order of exercise sessions will be used as random effect in the mix-linear model. Pairwise comparisons will be performed when significant main or interaction effects are detected, using Bonferroni correction.

However, during data analysis, the aforementioned statistical plan was amended given the nature of the data (not normally distributed data). Particularly, a significant number of the control subjects responded “zero” change in the visual analog scale assessing the symptom exacerbation. As such, non-parametric tests (Mann-Whitney test, Kruskal-Wallis test) were used to compare differences in symptoms following exercise trials between the groups.

A mixed-linear model was used to analyze differences in continuous data (CPET variables, interleukin (IL)-6, Creatine Kinase) following three exercise sessions (HIIT, MICT, ST) between the two groups (post-COVID-19 patients vs. control). Subject ID and order of exercise sessions were used as random effect in the mix-linear model. Pairwise comparisons will be performed when significant main or interaction effects are detected, using Bonferroni correction.

For the physiological characterization assessments, two-tailed Chi-square test or Student t-test will be used to examine differences in categorical or continuous variables, respectively, between the post-COVID-19 patients and the control group.

Results will be presented as mean (SD), and significance will be set at $P<0.05$.

Missing data will be treated as missing values in the statistical models, unless otherwise specified. In retrospect, there was no need for missing data imputation. Therefore, missing data were treated as missing values.

21. DATA MONITORING

Data will be saved protected on the Department of Laboratory Medicine's server for storing research data (Redcap and Electronic lab notebook; ELN). Only responsible researchers have access to data and code key. The code key is kept with the responsible researcher. All essential data processing procedures are documented completely, clearly, and verifiably at all times. Only the study team has access to the electronic data.

22. HARM (BENEFIT RISK CONCIDERATION)

The degree of exertion when measuring VO_{2peak} can give a certain feeling of discomfort in the chest and/or in working muscles. Dizziness may possibly occur with this test, but it is uncommon. Careful screening ensures that we do not include

people who are at increased risk of serious exercise related adverse events (for example, current or previous cardiovascular disease). All tests will be performed in a hospital setting, with immediate access to an emergency department. Symptoms and side effects are reported immediately to the responsible researcher and responsible physician. Specifically, in case of adverse events during the study visits, the research assistant and/or clinical staff will inform immediately the PI and responsible physician. The responsible physician will decide the appropriate sequel to solve the incidence. All details of the adverse event will be added to the medical records of the patient. PI is responsible for keeping records of the adverse event(s), including the steps taken by the research team. The strength tests performed may cause some discomfort in the leg muscles (muscle fatigue). However, these tests are associated with minimal medical risks. Exercise soreness can occur up to 4–5 days after the tests are performed. The risk of muscle, bone, joint and/or other soft tissue damage during controlled exercise is considered minimal. Needle EMG is a minimally invasive procedure that is carried out according to the clinical routines. All physiological tests are performed in a hospital environment and according to strict procedures. Muscle biopsies can in rare cases cause temporary loss of sensation. Specific care instructions are given to the research subjects. The machines are cleaned and disinfected after each use. Aggravation of symptoms and a feeling of malaise can be expected in some patients after exercise, and therefore it is important that they have contact details for the responsible physician and responsible researchers.

In regards to the direct benefits, the research subjects learn more about their current physiological function, their work capacity and their strength. The researchers also gain knowledge about what type of training is best tolerated for the individual. The potential clinical significance of this project is high, and we believe that the benefits substantially outweigh the risks of the project. Patients will be informed that their participation is voluntary, and that withdrawal will have no negative consequences for their usual treatment. There is great potential for benefit to the individual who learns more about their condition, exercise tolerance and current physiological function. Financial compensation of 1000 SEK will be given to participants for the discomfort that may possibly occur during muscle and blood sampling. It is paid after the research subject's last visit in the study.

All processions will be taken to minimize the risks as far as possible. For the tests that will be carried out, there are established test protocols that have been used for several years in our laboratory. Most examinations are routine clinical examinations performed in a hospital environment by trained personnel. Only people considered healthy enough to participate are included (see inclusion/exclusion criteria). Participants receive a direct number to the medical responsible physician (Thomas Gustafsson) and the responsible researcher for any questions or symptoms that arise during the tests or at home after exertion. After each training session, the patients remain in our laboratory for monitoring and blood samples. They also return to the lab for follow-up 48 hours after exercise. We therefore have continuous contact with the subjects, and they also have a direct number where they can reach us at any time from home. Muscle biopsies and needle biopsy for EMG are performed according to standard procedures. We have over 10 years of experience with these samplings without complications. The research project takes place in a

hospital environment (Karolinska University Hospital in Huddinge). In the event of unexpected side findings or complications, this is noted in the patient record and the responsible physician can prescribe appropriate referrals for further examination or call emergency personnel.

23. AUDITING

Study does not require external auditing. However, auditing within the research team (internally) will be conducted. Principal investigator and project manager is responsible for performing frequent auding (monthly basis) in all data storages (see data management), ensuring that all raw material is in place for each subject.

24. RESEARCH ETHICS APPROVAL

This study has been approved by the Swedish Ethical Authority REF: 2021 05758-01 and 2022-02999-02.

25. PROTOCOL AMENDMENTS

Amendments have been approved by the Swedish Ethical Authority REF: 2022-02999-02. This study is registered in ClinicalTrials.gov NCT05445830.

The study has registered and begun following the ethical approval of the version 1.1 amendments.

The statistical plan was revised during the data analysis (Aug 2023), given that the primary outcome (post-exercise symptom exacerbation via visual analog scale) required a different statistical approach (see above).

26. CONSENT

Patients attending the outpatient post-COVID-19 clinic at Karolinska University Hospital Huddinge will be informed of the current study and the opportunity to participate. Control subjects as well as post-COVID-19 patients if needed will receive information about this study through social channels and/or word of mouth. Interested patients will be in contact with the research team for further information. They will get written information via e-mail or letter. After that, the patient is given the opportunity to think and ask questions. If the person is still interested in participating, a first meeting is booked. The person then receives oral information from the responsible researcher and a chance to ask more questions if needed. The person is informed that they are free to cancel the study at any time without

having to give reasons. If the person still wants to participate, this consent is documented in writing. The screening procedure then follows.

27. CONFIDENTIALITY

The requirements of the Data Protection Law are fulfilled. It is ensured that all research materials and data are adequately pseudonymized in accordance with data protection regulations prior to scientific utilization. All data is saved electronically via RedCap. All data that is collected is saved with the study ID of the research subjects as a label (sequential number). Names and personal details are not disclosed. Only involved researchers have access to data material. The code list to identify the research subjects is saved as a password-protected document with a paper copy saved in a study folder locked with the responsible researcher. The responsible researcher is responsible for all documents being archived in accordance with KI's rules for research documentation and archiving of research data (saved for at least 10 years).

28. DECLARATION OF INTERESTS

No financial or other competing interests related to this study are declared by principal investigators and/or any member of the research team.

29. ACCESS TO THE DATA

Access to the data will be limited to the research team or anyone involved in the data analysis. Importantly, data will be pseudonymized, and none except the research team will have access to the personal data on the subjects. Therefore, Individual research subjects cannot be identified. Data will be stored in accordance with the Karolinska Institutet guidelines for clinical research data.

30. ANCILLARY AND POST-TRIAL CARE

All assessments will be conducted in the hospital setting and medical personnel will be available at any time needed. All visits/assessments will be logged in the TakeCare (medical record system). In the event of unexpected side findings or complications, this is noted in the patient record and the responsible physician can prescribe appropriate referrals for further examination or call emergency personnel. Patient insurance is in place.

31. DISSIMINATION POLICY

The results will be compiled in scientific articles that will be sent to a peer-reviewed scientific journals (Open access) for assessment and possible publication as soon as the data collection and analysis work has been completed. The results can also

be communicated before publication via channels in the Stockholm region if there is a large patient benefit in the short term. Results are presented only at the group level or with fully de-identified data points.

32. APPENDICES

Approved participant information sheet (consent form) is presented in Appendix 1. Original document is in Swedish, followed by English translation for the purposes of this supplementary material.

33. BIOLOGICAL SPECIMENTS

A maximum of 30 mL of blood is collected at each time point. In total, a maximum of 420 mL of blood is collected from each individual over the course of the study. (30 mL x 3 training sessions x 3 samples + baseline and 1-year follow-up). Muscle samples are taken from the outer thigh muscle, approx. 100–200 mg per sample on two occasions: baseline and at 1-year follow-up. Upon collection, samples will be stored in -80°C until further analysis. Samples will be stored in the Biobank Region Stockholm in accordance with the guidelines for a maximum period of 10 years. As such secondary questions not yet identified may be of interest in the future because post-COVID-19 is a new phenomenon.

APPENDIX 1 – CONSENT FORM ORIGINAL LANGUAGE (Swedish)

**Personal information such as researchers' phone numbers/emails are hidden.*

Information till forskningspersonerna

Du har fått muntlig information om studien *Fysiologisk karakterisering av funktionella begränsningar och träningsintolerans hos post-COVID patienter.* I det här dokumentet får du information om forskningsprojektet och vad det innebär att delta.

Vad är det för projekt och varför vill ni att jag ska delta?

Många patienter som drabbats av post-COVID syndromet (långtids-covid) upplever att deras symptom förvärras vid träning. De övergripande målen med detta projekt är att förbättra den fysiologiska förståelsen av symtom vid "långtids-Covid" samt undersöka förklaringsmekanismer för den träningsintolerans/symptomförvärring som ofta rapporteras efter fysisk ansträngning. För att undersöka detta kommer vi från ett omfattande fysiologiskt perspektiv att karakterisera de funktionella begränsningarna som patienter upplever, samt undersöka det fysiologiska svaret på olika träningspass och relatera dessa till ansträngningssymtom. Du har fått information om denna studie vid den mottagning du sökt dig till för dina besvär eller sett anslag på allmän plats och har därefter kontaktat oss för att få veta mer om studien. Vi söker dig som är i ålder 18–64 år och som är fullt frisk och kan medverka i en kontrollgrupp eller som har haft ihållande symptom längre än 3 månader efter verifierad covid-19 infektion.

Forskningshuvudman för projektet är Karolinska Institutet. Med forskningshuvudman menas den organisation som är ansvarig för studien. Huvudman för organisationen där de kliniska undersökningarna görs är Region Stockholm (Karolinska Universitetssjukhuset i Huddinge). Ansökan är godkänd av Etikprövningsmyndigheten, diarienummer för prövningen hos Etikprövningsmyndigheten är 2022-02999-02.

Hur går projektet till?

Deltagande innebär att du kommer undersökas med olika fysiologiska mätningar samt att utföra tre olika typer av träningspass. Den första gången kommer du att informeras muntligt om studien samt ges möjlighet att ställa kompletterande frågor innan vi tillfrågar dig om deltagande, som i så fall dokumenteras skriftligen (informerade samtycke). Därefter får du fylla i en hälsoenkät och samtala med en läkare som slutligen bedömer om du kan inkluderas i studien. Testerna som genomförs i studien är neurofysiologisk funktion, cirkulatorisk funktion, bestämning av blodvolym, syreupptag och andning vid arbete, styrka, blodstatus, ultraljud av hjärta och spirometri. Du kommer även att få fylla i frågeformulär för att bedöma dina symtom under och efter ansträngning (träning). Alla tester är etablerade kliniska tester alternativt utförs regelbundet i vårt laboratorium. Vid några av testerna kommer du att anstränga dig fysiskt och ta i så mycket du förmår. Träningspassen är 3 stycken (kontinuerlig konditionsträning på cykel (måttlig intensitet), intervallträning med hög intensitet på cykel, samt styrketräning med vikter/maskiner), med minst en vecka emellan. Ett till 2 dygn efter

varje träningspass kommer du tillbaka till vårt laboratorium för uppföljande undersökningar hur du svarat på träningspasset. Totalt innebär deltagandet att du kommer på besök till oss vid 8 olika tillfällen under en 1-årsperiod. Vid varje besök får du avsätta ungefär en halvdag. Alla deltagare genomgår alltså en ny undersökning efter 1 års uppföljning.

Möjliga följder och risker med att delta i studien

Ansträngningsgraden vid mätning av maximal syreupptagningsförmåga kan ge en viss obehagskänsla i bröstet och i arbetande muskler. Yrsel kan uppstå även om det är ovanligt. Styrketesterna som utförs kan ge visst obehag i benmusklerna på grund av trötthet. Dessa tester är förenade med mycket små medicinska risker. Lättare träningsvärk kan förekomma efter att testerna utförts. Alla fysiologiska tester genomförs enligt etablerade protokoll och övervakas av erfaren personal och utförs i sjukhusmiljö. Vid den neurologiska undersökningen sticks en liten nål genom huden, vilket kan upplevas som obehagligt eller ge upphov till smärre smärta. Muskelbiopsiprovet tas från yttre delen av lårmuskeln. Efter lokalbedövning görs ett litet snitt i huden och i den hinna som omger muskeln. En biopsinål (5 mm) förs in i muskeln och ett litet vävnadsprov (ca. 100-200 mg eller mindre än ett russin) tas ut. När en muskelbiopsi tas finns det en risk för mindre lokal blödning, infektion och ärrbildning av huden. Tillfälligt känselbortfall kring det område där biopsin tas sker i mycket enstaka fall. Direkt efter biopsitagning appliceras tryckförband, vilket syftar till att reducera risken för mindre lokal blödning. Vi kommer att minimera besvären så långt som möjligt genom optimalt omhändertagande av tränad personal. I samband med biopsiprovet kan en förnimmelse av ökat tryck kännas i muskeln. Efter provet kan du känna en viss stelhet i muskeln som kan kvarstå upp till två-fyra dagar. Totalt tas två muskelbiopsier, en vid baslinjetest samt en vid 1-årsuppföljningen. Blodprov tas vid varje besök (maximalt 30 ml per gång). Provet tas från armen precis som brukligt när man lämnar blodprov på en vårdinrättning.

Du kommer att få kontaktuppgifter till studiens medicinskt ansvarige läkare som du kan nå för att diskutera eventuella medicinska frågor eller problem som uppstår under studien.

Vad händer med mina uppgifter?

Projektet kommer att samla in och registrera information om dig. Den information vi kommer samla in är kön, ålder, vikt, kontaktuppgifter och hälsostatus i form av en hälsodeklaration, samt de data vi samlar in vid testerna. Information och data i studien kommer att pseudoanonymiseras vilket innebär att de endast går att härleda till dig genom en kodnyckel som sparas utan möjlighet till åtkomst för obehöriga. Uppgifterna skyddas så att endast de ansvariga forskarna kan komma åt dem. Personuppgifterna samlas in i enlighet med EU:s dataskyddsförordning GDPR. Den rättsliga grunden för att samla in uppgifter om dig består i att vi ska kunna utföra en uppgift av allmänt intresse (forskning).
Dina svar och dina resultat kommer att behandlas så att obehöriga inte kan ta del av dem. Ansvarig för dina personuppgifter är Karolinska Institutet. Enligt EU:s dataskyddsförordning har du rätt att kostnadsfritt få ta del av de uppgifter om dig som hanteras i studien, och vid behov få eventuella fel rättade. Du kan också begära att uppgifter om dig raderas samt att

behandlingen av dina personuppgifter begränsas. Om du vill ta del av uppgifterna ska du kontakta [redacted]. Dataskyddsombud på Karolinska Institutet Mats Gustavsson nås på [redacted]
Dataskyddsombud på Karolinska Universitetssjukhuset nås via dataskyddsombud.karolinska@regionstockholm.se Om du är missnöjd med hur dina personuppgifter behandlas har du rätt att ge in klagomål till Integritetsskyddsmyndigheten, som är tillsynsmyndighet.

Vad händer med mina prover?

De prover som tas i projektet förvaras kodade i en så kallad biobank (Biobankslag 2023:38). Biobankens namn är Stockholms medicinska biobank, nummer 914 och vår provsamling är placerad vid Karolinska Universitetssjukhuset i Huddinge. Huvudman (ansvarig) för biobanken är Region Stockholm.

Samtliga ovan nämnda prov kommer att vara kodade (pseudonymiserade) vilket innebär att de inte kan kopplas direkt till dig som person. Kodnyckel förvaras hos ansvarig forskare på Karolinska Institutet (Tommy Lundberg). Kodnyckeln behandlas så att inte obehöriga kan ta del av dem.

Du har rätt att utan förklaring säga nej till att proverna sparas. Om du samtycker till att proverna sparas har du rätt att senare och utan förklaring ta tillbaka (ångra) det samtycket. Dina prover kommer i så fall att kastas. Om du vill ångra ett samtycke ska du kontakta huvudansvarig forskare [redacted].

Proverna får bara användas på det sätt som du har gett samtycke till. Om du godkänner att vi får bevara och använda dina prover för framtida ändamål måste du samtycka specifikt till detta. Tillkommer forskning som ännu inte är planerad, kommer Etikprövningsmyndigheten att besluta om du ska tillfrågas på nytt.

Hur får jag information om resultatet av projektet?

Efter studiens slut kan du, om du vill, få muntlig och skriftlig återkoppling kring dina resultatet i testerna. Du tar då kontakt med ansvarig forskare Tommy Lundberg. Detta är helt frivilligt. Om vi upptäcker något oförutsett fynd som gäller dig kommer vi att kontakta dig.

Försäkring och ersättning

Det utgår en ersättning på 1000 kronor för deltagande i studien. Denna ersättning kompenserar för de obehag som möjligen kan uppstå vid provtagning eller ansträngning under studien. Patientförsäkring gäller under hela ditt deltagande i studien.

Deltagandet är frivilligt

Ditt deltagande är frivilligt och du kan när som helst välja att avbryta deltagandet. Om du väljer att inte delta eller vill avbryta ditt deltagande behöver du inte uppge varför, och det kommer inte heller att påverka din framtida vård eller behandling.

Om du vill avbryta ditt deltagande ska du kontakta den ansvariga för studien (se nedan).

Ansvariga för studien

Ansvarig forskare för studien är:

Tommy Lundberg, PhD, docent
Institutionen för Laboratoriemedicin / ANA Futura
Avdelningen för klinisk fysiologi
Alfred Nobels allé 8
141 52 Huddinge
[redacted]

Biträdande forskare och medicinskt ansvarig läkare:

Thomas Gustafsson, professor och överläkare
Fysiologkliniken Huddinge
[redacted]

Samtycke till att delta i projektet

Jag har fått muntlig och skriftlig informationen om studien och har haft möjlighet att ställa frågor. Jag får behålla den skriftliga informationen.

- Jag samtycker till att delta i studien *” Fysiologisk karakterisering av funktionella begränsningar och träningsintolerans hos post-COVID patienter”*
- Jag samtycker till att uppgifter om mig behandlas på det sätt som beskrivs i forskningspersonsinformationen.
- Jag samtycker till att mina prover sparas i en biobank på det sätt som beskrivs i forskningspersonsinformationen.
- Jag godkänner att mina prover sparas för eventuell framtida forskning.

Ja ☐ Nej ☐

Plats och datum	Underskrift
	Namnförtydligande

Participant information sheet (consent form) translated in English for the purpose of this Supplementary material study protocol.

Information for the research subjects

You have received oral information about the study *Physiological characterization of functional limitations and exercise intolerance in post-COVID patients.* In this document you will receive information about the research project and what it means to participate.

What kind of project is it and why do you want me to participate?

Many patients affected by the post-COVID syndrome (long-term covid) experience that their symptoms worsen with exercise. The overall goals of this project are to improve the physiological understanding of symptoms in "long-term Covid" and to investigate explanatory mechanisms for the exercise intolerance/symptom aggravation that is often reported after physical exertion. To investigate this, we will from a comprehensive physiological perspective characterize the functional limitations that patients experience, as well as investigate the physiological response to different training sessions and relate these to exertional symptoms. You have received information about this study at the reception you applied to for your problems or seen grants in a public place and have subsequently contacted us to find out more about the study. We are looking for you who are aged 18–64 and who are completely healthy and can participate in a control group or who have had persistent symptoms longer than 3 months after verified covid-19 infection.

The research lead for the project is the Karolinska Institutet. Research principal means the organization responsible for the study. The head of the organization where the clinical examinations are carried out is Region Stockholm (Karolinska University Hospital in Huddinge). The application has been approved by the Ethics Review Authority, the diary number for the review at the Ethics Review Authority is 2022-02999-02.

How is the project going?

Participation means that you will be examined with various physiological measurements and perform three different types of training sessions. The first time you will be informed verbally about the study and given the opportunity to ask supplementary questions before we ask you about participation, which in that case will be documented in writing (informed consent). You will then fill in a health questionnaire and talk to a doctor who will finally assess whether you can be included in the study. The tests carried out in the study are neurophysiological function, circulatory function, determination of blood volume, oxygen uptake and breathing during work, strength, blood status, ultrasound of the heart and spirometry. You will also be asked to complete questionnaires to assess your symptoms during and after exertion (exercise). All tests are established clinical tests or are performed regularly in our laboratory. In some of the tests, you will exert yourself physically and take in as much as you can. There are 3 training sessions (continuous cardio training on a bicycle (moderate intensity), interval training with high intensity on a bicycle, and strength training with weights/machines), with at

least one week in between. One to 2 days after each training session, you will return to our laboratory for follow-up examinations on how you responded to the training session. In total, participation means that you will visit us on 8 different occasions during a 1-year period. For each visit, you can set aside about half a day. All participants thus undergo a new examination after 1 year of follow-up.

Possible consequences and risks of participating in the study

The level of exertion when measuring the maximum oxygen uptake capacity can give a certain feeling of discomfort in the chest and in working muscles. Dizziness may occur although it is uncommon. The strength tests performed may cause some discomfort in the leg muscles due to fatigue. These tests carry very little medical risk. Slight soreness may occur after the tests are performed. All physiological tests are performed according to established protocols and supervised by experienced staff and performed in a hospital environment. During the neurological examination, a small needle is inserted through the skin, which can be experienced as unpleasant or give rise to minor pain. The muscle biopsy sample is taken from the outer part of the thigh muscle. After local anesthesia, a small incision is made in the skin and in the membrane that surrounds the muscle. A biopsy needle (5 mm) is inserted into the muscle and a small tissue sample (about 100-200 mg or smaller than a raisin) is removed. When a muscle biopsy is taken, there is a risk of minor local bleeding, infection and scarring of the skin. Temporary loss of sensation around the area where the biopsy is taken occurs in very rare cases. Directly after biopsy, a pressure bandage is applied, which aims to reduce the risk of minor local bleeding. We will minimize the inconvenience as far as possible through optimal care by trained personnel. In connection with the biopsy sample, a sensation of increased pressure may be felt in the muscle. After the test, you may feel some stiffness in the muscle, which may persist for up to two to four days. In total, two muscle biopsies are taken, one at the baseline test and one at the 1-year follow-up. Blood samples are taken at each visit (maximum 30 ml per time). The sample is taken from the arm just as usual when you leave a blood sample at a healthcare facility.

You will receive contact information for the study's medically responsible physician who you can reach to discuss any medical questions or problems that arise during the study.

What happens to my data?

The project will collect and register information about you. The information we will collect is gender, age, weight, contact details and health status in the form of a health declaration, as well as the data we collect during the tests. Information and data in the study will be pseudo-anonymized , which means that they can only be traced back to you through a code key that is saved without the possibility of unauthorized access. The data is protected so that only the responsible researchers can access it. The personal data is collected in accordance with the EU data protection regulation GDPR. The legal basis for collecting information about you is that we should be able to carry out a task of public interest (research).
Your answers and results will be processed so that unauthorized persons cannot access them.

Responsible for your personal data is Karolinska Institutet. According to the EU's data protection regulation, you have the right to access the information about you that is handled in the study free of charge, and if necessary to have any errors corrected. You can also request that information about you be deleted and that the processing of your personal data be restricted. If you want to share the information, please contact [redacted]. Data Protection Officer at Karolinska Institutet Mats Gustavsson can be reached at [redacted] The Data Protection Officer at Karolinska University Hospital can be reached via dataskyddsombud.karolinska@regionstockholm.se If you are dissatisfied with how your personal data is processed, you have the right to file a complaint with the Swedish Privacy Protection Authority, which is the supervisory authority.

What happens to my samples?

The samples taken in the project are stored coded in a so-called biobank (Biobank Act 2023:38). The biobank's name is Stockholms medicinska biobank, nummer 914and our sample collection is located at Karolinska University Hospital in Huddinge. The head (responsible) for the biobank is Region Stockholm.

All of the above-mentioned tests will be coded (pseudonymized), which means that they cannot be linked directly to you as a person. Code key is kept with responsible researcher at Karolinska Institutet (Tommy Lundberg). The code key is processed so that unauthorized persons cannot access it.

You have the right to say no to the samples being saved without explanation. If you consent to the samples being saved, you have the right to withdraw (revoke) that consent later and without explanation. Your samples will then be thrown away. If you want to withdraw consent, you must contact the researcher in charge [redacted].

The samples may only be used in the manner for which you have given consent. If you agree that we may retain and use your samples for future purposes, you must specifically consent to this. If research is added that is not yet planned, the Ethics Review Authority will decide whether you should be asked again.

How do I receive information about the results of the project?

After the end of the study, you can, if you wish, receive oral and written feedback about your results in the tests. You then contact responsible researcher Tommy Lundberg. This is completely voluntary. If we discover any unexpected findings that apply to you, we will contact you.

Insurance and compensation

A compensation of SEK 1,000 is paid for participation in the study. This compensation compensates for the discomfort that may possibly occur during sampling or exertion during the study. Patient insurance applies throughout your participation in the study.

Participation is voluntary

Your participation is voluntary and you can choose to cancel your participation at any time. If you choose not to participate or wish to cancel your participation, you do not need to state why, and it will not affect your future care or treatment.

If you wish to cancel your participation, please contact the person responsible for the study (see below).

Responsible for the study

Responsible researcher for the study is:

Tommy Lundberg , PhD, docent
Department of Laboratory Medicine / ANA Futura
Department of Clinical Physiology
Alfred Nobels allé 8
141 52 Huddinge
[redacted]

Assistant researcher and medically responsible physician:

Thomas Gustafsson, professor and chief physician
Physiologist Clinic Huddinge
[redacted]

Consent to participate in the project

I have received oral and written information about the study and have had the opportunity to ask questions. I get to keep the written information.

- I agree to participate in the study *"Physiological characterization of functional limitations and exercise intolerance in post-COVID patients"*
- I agree to data about me being processed in the manner described in the research subject information.
- I consent to my samples being stored in a biobank in the manner described in the research subject information.
- I consent to my samples being saved for possible future research.

☐ Yes ☐ No

Place and date	Signature
	Name clarification

REFERENCES

1. Huang C, Huang L, Wang Y, et al. 6-month consequences of COVID-19 in patients discharged from hospital: a cohort study. *Lancet*. Jan 16 2021;397(10270):220-232. doi:10.1016/s0140-6736(20)32656-8

2. Sudre CH, Murray B, Varsavsky T, et al. Attributes and predictors of long COVID. *Nat Med*. Apr 2021;27(4):626-631. doi:10.1038/s41591-021-01292-y

3. Arnold DT, Hamilton FW, Milne A, et al. Patient outcomes after hospitalisation with COVID-19 and implications for follow-up: results from a prospective UK cohort. *Thorax*. Apr 2021;76(4):399-401. doi:10.1136/thoraxjnl-2020-216086

4. Goërtz YMJ, Van Herck M, Delbressine JM, et al. Persistent symptoms 3 months after a SARS-CoV-2 infection: the post-COVID-19 syndrome? *ERJ Open Res*. Oct 2020;6(4)doi:10.1183/23120541.00542-2020

5. Physiotherapy W. World Physiotherapy Response to COVID-19 Briefing Paper 9. *Safe rehabilitation approaches for people living with Long COVID: physical activity and exercise London, UK*. 2021;

6. Motiejunaite J, Balagny P, Arnoult F, et al. Hyperventilation: A Possible Explanation for Long-Lasting Exercise Intolerance in Mild COVID-19 Survivors? *Front Physiol*. 2020;11:614590. doi:10.3389/fphys.2020.614590

7. Goldstein DS. The possible association between COVID-19 and postural tachycardia syndrome. *Heart Rhythm*. Apr 2021;18(4):508-509. doi:10.1016/j.hrthm.2020.12.007

8. Olshansky B, Cannom D, Fedorowski A, et al. Postural Orthostatic Tachycardia Syndrome (POTS): A critical assessment. *Prog Cardiovasc Dis*. May-Jun 2020;63(3):263-270. doi:10.1016/j.pcad.2020.03.010

9. Twomey R, DeMars J, Franklin K, Culos-Reed SN, Weatherald J, Wrightson JG. Chronic Fatigue and Postexertional Malaise in People Living With Long COVID: An Observational Study. *Phys Ther*. Apr 1 2022;102(4)doi:10.1093/ptj/pzac005

10. Mandarano AH, Maya J, Giloteaux L, et al. Myalgic encephalomyelitis/chronic fatigue syndrome patients exhibit altered T cell metabolism and cytokine associations. *J Clin Invest*. Mar 2 2020;130(3):1491-1505. doi:10.1172/jci132185

11. Smets EM, Garssen B, Bonke B, De Haes JC. The Multidimensional Fatigue Inventory (MFI) psychometric qualities of an instrument to assess fatigue. *J Psychosom Res*. Apr 1995;39(3):315-25. doi:10.1016/0022-3999(94)00125-o

12. McNair DM, Lorr M, Droppleman LF. *EdITS Manual for the Profile of Mood States (POMS)*. Educational and industrial testing service; 1992.

13. Hickie I, Hadzi-Pavlovic D, Scott E, Davenport T, Koschera A, Naismith S. SPHERE: a national depression project. *Australasian Psychiatry*. 1998;6(5):248-250.

14. ATS statement: guidelines for the six-minute walk test. *Am J Respir Crit Care Med*. Jul 1 2002;166(1):111-7. doi:10.1164/ajrccm.166.1.at1102

15. Choi L, Liu Z, Matthews CE, Buchowski MS. Validation of accelerometer wear and nonwear time classification algorithm. *Med Sci Sports Exerc*. Feb 2011;43(2):357-64. doi:10.1249/MSS.0b013e3181ed61a3

16. Sheldon RS, Grubb BP, 2nd, Olshansky B, et al. 2015 heart rhythm society expert consensus statement on the diagnosis and treatment of postural tachycardia syndrome, inappropriate sinus tachycardia, and vasovagal syncope. *Heart Rhythm*. Jun 2015;12(6):e41-63. doi:10.1016/j.hrthm.2015.03.029

17. Vernino S, Bourne KM, Stiles LE, et al. Postural orthostatic tachycardia syndrome (POTS): State of the science and clinical care from a 2019 National Institutes of Health Expert Consensus Meeting - Part 1. *Autonomic Neuroscience*. 2021/11/01/ 2021;235:102828. doi:https://doi.org/10.1016/j.autneu.2021.102828

18. Finucane C, van Wijnen VK, Fan CW, et al. A practical guide to active stand testing and analysis using continuous beat-to-beat non-invasive blood pressure monitoring. *Clin Auton Res*. Aug 2019;29(4):427-441. doi:10.1007/s10286-019-00606-y

19. Graham BL, Steenbruggen I, Miller MR, et al. Standardization of Spirometry 2019 Update. An Official American Thoracic Society and European Respiratory Society Technical Statement. *Am J Respir Crit Care Med*. Oct 15 2019;200(8):e70-e88. doi:10.1164/rccm.201908-1590ST

20. Balady GJ, Arena R, Sietsema K, et al. Clinician's Guide to cardiopulmonary exercise testing in adults: a scientific statement from the American Heart Association. *Circulation*. Jul 13 2010;122(2):191-225. doi:10.1161/CIR.0b013e3181e52e69

21. Edvardsen E, Hem E, Anderssen SA. End criteria for reaching maximal oxygen uptake must be strict and adjusted to sex and age: a cross-sectional study. *PLoS One*. 2014;9(1):e85276. doi:10.1371/journal.pone.0085276

22. Böcskei RM, Benczúr B, Müller V, et al. Oscillometrically Measured Aortic Pulse Wave Velocity Reveals Asymptomatic Carotid Atherosclerosis in a Middle-Aged, Apparently Healthy Population. *Biomed Res Int*. 2020;2020:8571062. doi:10.1155/2020/8571062

23. Keiser S, Meinild-Lundby AK, Steiner T, et al. Detection of blood volumes and haemoglobin mass by means of CO re-breathing and indocyanine green and sodium fluorescein injections. *Scand J Clin Lab Invest*. May 2017;77(3):164-174. doi:10.1080/00365513.2016.1271908

24. Prommer N, Schmidt W. Loss of CO from the intravascular bed and its impact on the optimised CO-rebreathing method. *Eur J Appl Physiol*. Jul 2007;100(4):383-91. doi:10.1007/s00421-007-0439-2

25. Mandić M, Eriksson LMJ, Melin M, et al. Increased maximal oxygen uptake after sprint-interval training is mediated by central haemodynamic factors as determined by right heart catheterization. *J Physiol*. Jun 2023;601(12):2359-2370. doi:10.1113/jp283807

26. Lindheimer JB, Meyer JD, Stegner AJ, et al. Symptom variability following acute exercise in myalgic encephalomyelitis/chronic fatigue syndrome: a perspective on measuring post-exertion malaise. *Fatigue: Biomedicine, Health & Behavior*. 2017;5(2):69-88.

Print Form

CONSORT CHECKLIST

Table. CONSORT 2010 Checklist of Information to Include When Reporting a Randomized Trial[a]

Section and Topic	Item No.	Checklist Item	Reported on Page No.
**Title and abstract**			
	1a	Identification as a randomized trial in the title	1
	1b	Structured summary of trial design, methods, results, and conclusions (for specific guidance see CONSORT for abstracts)	2
**Introduction**			
Background and objectives	2a	Scientific background and explanation of rationale	4
	2b	Specific objectives or hypotheses	4, S12
**Methods**			
Trial design	3a	Description of trial design (such as parallel, factorial) including allocation ratio	5
	3b	Important changes to methods after trial commencement (such as eligibility criteria), with reasons	NA
Participants	4a	Eligibility criteria for participants	4-5
	4b	Settings and locations where the data were collected	4-5
Interventions	5	The interventions for each group with sufficient details to allow replication, including how and when they were actually administered	S16-22
Outcomes	6a	Completely defined prespecified primary and secondary outcome measures, including how and when they were assessed	5, S12
	6b	Any changes to trial outcomes after the trial commenced, with reasons	NA
Sample size	7a	How sample size was determined	S23
	7b	When applicable, explanation of any interim analyses and stopping guidelines	NA
Randomization			
Sequence generation	8a	Method used to generate the random allocation sequence	S23
	8b	Type of randomization; details of any restriction (such as blocking and block size)	S23
Allocation concealment mechanism	9	Mechanism used to implement the random allocation sequence (such as sequentially numbered containers), describing any steps taken to conceal the sequence until interventions were assigned	S23
Implementation	10	Who generated the random allocation sequence, who enrolled participants, and who assigned participants to interventions	S23
Blinding	11a	If done, who was blinded after assignment to interventions (for example, participants, care providers, those assessing outcomes) and how	S23
	11b	If relevant, description of the similarity of interventions	S15
Statistical methods	12a	Statistical methods used to compare groups for primary and secondary outcomes	6, S24
	12b	Methods for additional analyses, such as subgroup analyses and adjusted analyses	6-7
**Results**			
Participant flow (a diagram is strongly recommended)	13a	For each group, the numbers of participants who were randomly assigned, received intended treatment, and were analyzed for the primary outcome	Figure1
	13b	For each group, losses and exclusions after randomization, together with reasons	Figure1
Recruitment	14a	Dates defining the periods of recruitment and follow-up	4-5
	14b	Why the trial ended or was stopped	NA
Baseline data	15	A table showing baseline demographic and clinical characteristics for each group	Table1
Numbers analyzed	16	For each group, number of participants (denominator) included in each analysis and whether the analysis was by original assigned groups	Figure1
Outcomes and estimation	17a	For each primary and secondary outcome, results for each group, and the estimated effect size and its precision (such as 95% confidence interval)	18-22
	17b	For binary outcomes, presentation of both absolute and relative effect sizes is recommended	18-20
Ancillary analyses	18	Results of any other analyses performed, including subgroup analyses and adjusted analyses, distinguishing prespecified from exploratory	7
Harms	19	All important harms or unintended effects in each group (for specific guidance see CONSORT for harms)	NA
**Comment**			
Limitations	20	Trial limitations, addressing sources of potential bias, imprecision, and, if relevant, multiplicity of analyses	9-11
Generalizability	21	Generalizability (external validity, applicability) of the trial findings	9-11
Interpretation	22	Interpretation consistent with results, balancing benefits and harms, and considering other relevant evidence	9-11
**Other information**			
Registration	23	Registration number and name of trial registry	5
Protocol	24	Where the full trial protocol can be accessed, if available	5
Funding	25	Sources of funding and other support (such as supply of drugs), role of funders	12

[a] We strongly recommend reading this statement in conjunction with the CONSORT 2010 Explanation and Elaboration for important clarifications on all the items. If relevant, we also recommend reading CONSORT extensions for cluster randomized trials, noninferiority and equivalence trials, nonpharmacological treatments, herbal interventions, and pragmatic trials. Additional extensions are forthcoming: for those and for up-to-date references relevant to this checklist, see http://www.consort-statement.org.

©2010 American Medical Association. All rights reserved.
