# Supplementary material for: Functional Limitations and Exercise Intolerance in Patients With Post-COVID Condition: A Randomized Crossover Clinical Trial
Source: JAMA Netw Open. 2024 Apr 4;7(4):e244386. doi: 10.1001/jamanetworkopen.2024.4386 (PMC11192186; doi:10.1001/jamanetworkopen.2024.4386)
Supplement: Supplement 2. — eTable 1. Participant Demographics eTable 2. Responses During Exercise Sessions eTable 3. Median Group Differences in 10 Symptoms Assessed With Visual Analog Scale Symptoms eTable 4. Median Exercise Differences in 10 Symptoms Assessed With Visual Analog Scale Symptoms eTable 5. Results From Postexertional Symptoms as Assessed With MFI, POMS, and SPHERE Questionnaires eTable 6. Mean Group Differences in Postexertional Symptoms Assessed With MFI, POMS, and SPHERE Questionnaires eTable 7. Variables From Cardiopulmonary Exercise Testing at 48 Hours After Exercise Sessions eTable 8. Mean Group Differences in Variables From the Cardiopulmonary Exercise Testing at 48 Hours After Exercise Sessions eTable 9. Mean Exercise Differences in Variables From the Cardiopulmonary Exercise Testing at 48 Hours After Exercise Sessions eTable 10. CK and IL-6 in Response to 3 Exercise Sessions eTable 11. Mean Group Differences Baseline Characteristics eTable 12. Supplementary Results From the Examinations Included in the Physiological Characterization eFigure 1. Individual Changes for Fatigue as Assessed via Visual Analog Scale in Response to 3 Exercise Sessions eFigure 2. Individual Changes for Muscle Pain as Assessed via Visual Analog Scale in Response to 3 Exercise Sessions [file jamanetwopen-e244386-s002.pdf]

Supplementary Online Content

Tryfonos A, Pourhamidi K, Jörnåker G, et al. Functional limitations and exercise intolerance in patients with post-COVID condition: a randomized crossover clinical trial. *JAMA Netw Open*. 2024;7(4):e244386. doi:10.1001/jamanetworkopen.2024.4386

eTable 1. Participant Demographics

eTable 2. Responses During Exercise Sessions

eTable 3. Median Group Differences in 10 Symptoms Assessed With Visual Analog Scale Symptoms

eTable 4. Median Exercise Differences in 10 Symptoms Assessed With Visual Analog Scale Symptoms

eTable 5. Results From Postexertional Symptoms as Assessed With MFI, POMS, and SPHERE Questionnaires

eTable 6. Mean Group Differences in Postexertional Symptoms Assessed With MFI, POMS, and SPHERE Questionnaires

eTable 7. Variables From Cardiopulmonary Exercise Testing at 48 Hours After Exercise Sessions

eTable 8. Mean Group Differences in Variables From the Cardiopulmonary Exercise Testing at 48 Hours After Exercise Sessions

eTable 9. Mean Exercise Differences in Variables From the Cardiopulmonary Exercise Testing at 48 Hours After Exercise Sessions

eTable 10. CK and IL-6 in Response to 3 Exercise Sessions

eTable 11. Mean Group Differences Baseline Characteristics

eTable 12. Supplementary Results From the Examinations Included in the Physiological Characterization

eFigure 1. Individual Changes for Fatigue as Assessed via Visual Analog Scale in Response to 3 Exercise Sessions

eFigure 2. Individual Changes for Muscle Pain as Assessed via Visual Analog Scale in Response to 3 Exercise Sessions

This supplementary material has been provided by the authors to give readers additional information about their work.

eTable 1. Participants' demographics retrospectively requested from the patients with post-covid condition (PCC) and age- and sex-matched healthy controls. Out of 31 PCC patients, 23 responded (74.2%), and 18 controls (58.1%).

	PCC n=23	Controls n=18
**Work status (study enrollment)**		
Full-time, N (%)	12 (52)	14 (78)
75% of full-time, N (%)	4 (17)	2 (11)
50% of full-time, N (%)	2 (9)	0 (0)
25% of full-time, N (%)	1 (4)	0 (0)
Unemployed, N (%)	0 (0)	0 (0)
Student, N (%)	3 (13)	1 (6)
Pensioner, N (%)	1 (4)	1 (6)
**ONLY FOR PCC - Has your work status changed due to symptoms related to PCC?**		
Yes, N (%)	22 (96)	
No, N (%)	1 (4)	
**Work status before Covid-19**		
Full-time, N (%)	18 (78)	
75% of full-time, N (%)	2 (9)	
50% of full-time, N (%)	0 (0)	
25% of full-time, N (%)	0 (0)	
Unemployed, N (%)	0 (0)	
Student, N (%)	2 (9)	
Pensioner, N (%)	1 (4)	
**ONLY FOR PCC - Have you been on sick leave due to symptoms related to PCC?**		
Yes, N (%)	17 (74)	
No, N (%)	6 (26)	
**Duration of sick leave**		
>2 weeks, N (%)	0 (0)	
2-4 weeks, N (%)	1 (4)	
>1 month, N (%)	1 (4)	
>6 months, N (%)	3 (13)	
>12 months, N (%)	17 (74)	
**ONLY FOR PCC - Have you sought medical care due to symptoms related to PCC?**		
Yes, N (%)	21 (91)	
No, N (%)	2 (9)	
**Category of medical care**		
Family doctor, N (%)	20 (87)	
Post-covid clinic, N (%)	11 (48)	
Physiotherapy, N (%)	16 (70)	
Psychology, N (%)	3 (13)	
Other, N (%)	7 (30)	
**Have your views on exercising changed during your participation in this study?**		
Yes, N (%)	13 (57)	1 (6)
No, N (%)	10 (43)	17 (94)

© 2024 Tryfonos A et al. *JAMA Network Open*.

	PCC n=23	Controls n=18
**Which of the following statements describes you best?**		
I try to exercise more now. N (%)	13 (57)	1 (6)
I do the same exercise as before. N (%)	6 (26)	17 (94)
I am afraid to exercise because I had symptoms while participating in the study. N (%)	4 (17)	0 (0)

© 2024 Tryfonos A et al. *JAMA Network Open*.

eTable 2. Responses during 3 exercise sessions. Data are presented as means (SD).

	HIIT		MICT		ST	
	**PCC n=27**	**Control n=31**	**PCC n=28**	**Control n=28**	**PCC n=27**	**Control n=31**
**HR, mean (SD), bpm**						
Rest	81 (11)	75 (9)	82 (11)	72 (9)	76 (10)	71 (12)
Peak	161 (12)	161 (16)	146 (15)	146 (16)	138 (18)	137 (21)
% estimated max	92 (12)	94 (9)	85 (9)	82 (19)	75 (12)	74 (11)
**Workload, mean (SD), W**						
Peak	161 (61)	216 (61)	85 (36)	119 (38)	NA	NA
$\%_{max}$	95 (15)	98 (8)	49 (8)	54 (2)	NA	NA
**RPE (Borg scale 6–20, mean (SD), score**						
Rest	7 (2)	6 (1)	7 (2)	7 (1)	8 (3)	6 (1)
Peak	18 (2)	18 (2)	16 (1)	15 (2)	18 (2)	17 (2)
**SaO_2, mean (SD), %**						
Rest	98 (1)	99 (1)	99 (1)	99 (1)	98 (2)	99 (1)
Peak	97 (3)	97 (2)	98 (1)	97 (2)	98 (2)	99 (1)
**SBP, mean (SD), mmHg**						
Rest	114 (9)	116 (14)	110 (9)	113 (10)	116 (15)	113 (12)
Peak	143 (16)	161 (20)	145 (14)	160 (15)	132 (24)	133 (19)
**Lactate concentration, mean (SD), mmol/L**						
Rest	0.9 (0.3)	1.0 (0.3)	1.0 (0.3)	1.2 (0.5)	1.1 (0.4)	1.1 (0.4)
Peak	8.6 (3.8)	8.8 (3.3)	2.5 (1.1)	3.6 (3.3)	7.1 (3.2)	6.8 (2.9)

HIIT: high-intensity interval training; MICT: moderate-intensity continuous training; ST: strength training; HR: heart rate; RPE: rating of perceived exertion; SaO_2: peripheral oxygen saturation; SBP: systolic blood pressure

© 2024 Tryfonos A et al. *JAMA Network Open*.

eTable 3: Mean group differences in delta (Δ) changes in symptoms from baseline to 48h post-exercise in patients with post-covid condition (PCC) and age- and sex-matched healthy controls. Symptoms were assessed using Visual Analog Scale (VAS) 0-10. Median (IQR) for each group and exercise are presented in Table 2 (manuscript). Results are presented as median differences (IQR).

	PCC vs. Control		
	**HIIT**	**MICT**	**ST**
	Median difference (IQR)	Median difference (IQR)	Median difference (IQR)
**Fatigue VAS 0-10**	0.0 (-1.0, 2.0)	0.0 (0.25, 2.0)	1.0 (-1.0, 2.0)
**Muslce pain VAS 0-10**	0.0 (0.25, 1.5)	0.0 (-0.5, 0.25)	2.0 (0.5, 0.0)
**Joint pain VAS 0-10**	0.0 (0.0, 0.0)	0.0 (-1.0, 1.0)	0.0 (-0.5, 1.5)
**Fever VAS 0-10**	0.0 (0.0, 0.0)	0.0 (-0.25, 0.0)	0.0 (0.0, 0.0)
**Chills VAS 0-10**	0.0 (0.0, 0.0)	0.0 (0.0, 0.25)	0.0 (0.0, 0.0)
**Lymph nodes VAS 0-10**	0.0 (0.0, 0.0)	0.0 (-0.25, 0.0)	0.0 (0.0, 0.5)
**Sore throat VAS 0-10**	0.0 (0.0, 0.0)	0.0 (0.0, 0.0)	0.0 (0.0, 0.0)
**Headache VAS 0-10**	0.0 (-1.0, 1.5)	0.5 (0.0, 2.0)	0.0 (0.0, 1.0)
**Memory VAS 0-10**	0.0 (-1.0, 1.0)	0.5 (-1.0, 2.0)	0.0 (-1.0, 1.0)
**Concentration VAS 0-10**	0.0 (-1.0, 1.0)	1.0 (0.0, 2.0)	-1.0 (-2.0, 1.0)

HIIT: high-intensity interval training; MICT: moderate-intensity continuous training; ST: strength training

© 2024 Tryfonos A et al. *JAMA Network Open*.

eTable 4: Mean exercise differences in delta (Δ) changes in symptoms from baseline to 48h after three exercise training (HIIT: high-intensity interval training, MICT: moderate-intensity continuous training, ST: strength training) in patients with post-covid condition (PCC) (n=30). Symptoms were assessed using Visual Analog Scale (VAS) 0-10. Median (IQR) for each exercise are presented in Table 2 (manuscript). Results are presented as median differences (IQR).

	Exercise effect		
	**HIIT vs. MICT** Median difference (IQR)	**HIIT vs. ST** Median difference (IQR)	**MICT vs. ST** Median difference (IQR)
**Fatigue VAS 0-10**	0.0 (-1.0, 1.0)	-1.0 (-2.0, 1.0)	-1.0 (-2.0, 2.0)
**Muslce pain VAS 0-10**	0.0 (0.0, 1.75)	-2.0 (-2.0, 0.0)	-2.0 (-3.0, -1.0)
**Joint pain VAS 0-10**	0.0 (-1.0, 1.0)	0.0 (-1.0, 1.0)	0.0 (-2.0, 1.0)
**Fever VAS 0-10**	0.0 (0.0, 0.0)	0.0 (-0.75, 0.75)	0.0 (-1.0, 0.0)
**Chills VAS 0-10**	0.0 (-0.75, 0.0)	0.0 (0.0, 0.0)	0.0 (0.0, 0.0)
**Lymph nodes VAS 0-10**	0.0 (0.0, 0.0)	0.0 (0.0, 0.0)	0.0 (-2.0, 0.0)
**Sore throat VAS 0-10**	0.0 (0.0, 0.0)	0.0 (-1.75, 0.0)	0.0 (-1.0, 0.0)
**Headache VAS 0-10**	-0.5 (-2.0, 1.0)	0.0 (-1.75, 0.0)	0.5 (-1.0, 2.0)
**Memory VAS 0-10**	-0.5 (-1.75, 1.0)	0.0 (-1.75, 1.0)	0.5 (-2.0, 2.0)
**Concentration VAS 0-10**	-1.0 (-2.75, 1.0)	1.0 (-1.0, 2.75)	2.0 (0.0, 3.0)

HIIT: high-intensity interval training; MICT: moderate-intensity continuous training; ST: strength training

© 2024 Tryfonos A et al. *JAMA Network Open*.

eTable 5. Delta changes (Δ) in symptoms from baseline (Pre) to immediate post- and 48h post-exercise in patients with post-covid condition (PCC) and age- and sex-matched healthy controls as assessed by a) Multifactional Fatigue Inventory (MFI), b) Profile of Mood States (POMS), and c) the Somatic (SOMA) and Psychological (PHYCH) Health REport (SPHERE). Data are presented as median (IQR).

	Exercise						P values				
	HIIT		MICT		ST		Δ change PCC vs. Control			Δ change all exercises	
	PCC	Control	PCC	Control	PCC	Control	HIIT	MICT	ST	PCC	Control
**MFI General Fatigue, median (IQR), score**											
**Pre**	16.0 (13.5, 17.0)	6.0 (5.0, 8.0)	15.5 (11.8, 17.2)	5.0 (3.0, 8.0)	15.0 (11.0, 18.0)	6.0 (3.5, 7.0)					
**Post**	16.5 (14.2, 18.0)	6.0 (4.5, 9.5)	14.0 (13.0, 16.0)	5.0 (3.0, 8.0)	16.0 (12.5, 18.0)	6.0 (3.0, 8.0)	0.34	0.58	0.57	0.62	0.88
**48h**	15.0 (13.0, 18.0)	6.0 (4.0, 7.8)	16.0 (14.0, 17.0)	5.0 (4.0, 7.0)	15.0 (13.0, 17.0)	6.0 (3.0, 8.5)	0.30	0.16	0.23	0.62	0.21
**MFI Physical Fatigue, median (IQR), score**											
**Pre**	14.0 (13.0, 17.0)	5.0 (3.0, 6.0)	14.0 (12.0, 17.2)	4.0 (3.0, 6.2)	15.0 (12.5, 17.5)	4.0 (3.0, 6.0)					
**Post**	15.5 (13.0, 17.0)	6.0 (3.0, 7.0)	14.0 (12.0, 16.0)	4.0 (3.0, 7.5)	16.0 (12.5, 18.0)	5.0 (3.0, 6.5)	0.42	0.40	0.53	0.80	0.38
**48h**	15.0 (13.0, 17.5)	4.0 (3.0, 6.0)	15.0 (13.0, 17.2)	4.0 (3.0, 6.0)	14.0 (12.5, 17.0)	5.0 (3.5, 7.5)	0.39	0.04	0.07	0.24	0.07
**MFI Reduced Activity, median (IQR), score**											
**Pre**	14.0 (10.5, 15.5)	5.0 (4.0, 8.0)	12.5 (9.8, 15.0)	4.0 (3.0, 7.0)	12.0 (10.0, 15.0)	5.0 (3.0, 6.0)					
**Post**	13.0 (10.0, 16.0)	6.0 (3.0, 8.5)	12.5 (9.8, 14.0)	3.0 (2.0, 6.0)	13.0 (11.0, 15.5)	4.0 (2.0, 7.0)	0.44	0.13	0.41	0.86	0.47
**48h**	14.0 (11.0, 15.0)	4.0 (3.0, 6.0)	14.0 (10.0, 15.0)	4.0 (3.0, 5.5)	12.0 (10.5, 14.0)	4.0 (2.5, 6.0)	0.08	0.01	0.71	0.35	0.86
**MFI Reduced Motivation, median (IQR), score**											
**Pre**	10.0 (7.5, 11.5)	4.0 (2.5, 6.5)	8.0 (5.8, 11.0)	3.5 (2.8, 7.0)	8.0 (5.0, 11.0)	4.0 (2.0, 6.0)					
**Post**	10.5 (9.0, 13.0)	4.0 (2.0, 6.0)	9.5 (6.0, 11.0)	3.0 (2.0, 5.5)	10.0 (7.0, 11.0)	4.0 (2.0, 5.0)	0.02	0.24	0.21	0.67	0.99
**48h**	10.0 (6.0, 13.0)	3.5 (2.0, 5.0)	10.0 (6.8, 12.0)	3.0 (2.0, 5.0)	9.0 (6.5, 11.0)	3.0 (2.0, 6.0)	0.26	0.002	0.23	0.85	0.21
**MFI Mental Fatigue, median (IQR), score**											
**Pre**	13.0 (11.0, 14.5)	5.0 (4.0, 7.0)	10.5 (8.8, 15.0)	5.0 (3.0, 7.0)	13.0 (11.5, 14.5)	4.0 (2.0, 6.5)					
**Post**	12.5 (9.2, 15.0)	6.0 (4.0, 8.0)	12.0 (10.0, 14.2)	4.0 (2.0, 7.0)	13.0 (11.5, 15.0)	4.0 (2.0, 7.0)	0.57	0.07	0.97	0.56	0.16
**48h**	13.0 (11.0, 15.0)	4.5 (2.0, 6.8)	13.0 (10.8, 15.0)	3.0 (2.0, 6.0)	13.0 (10.0, 14.0)	3.0 (2.0, 6.5)	0.22	<0.001	0.25	0.01	0.06
**MFI Total, median (IQR), score**											
**Pre**	65.0 (59.0, 74.5)	24.0 (19.0, 33.5)	62.0 (48.8, 71.5)	22.0 (17.0, 30.0)	64.0 (54.5, 72.5)	23.0 (17.5, 31.5)					
**Post**	68.5 (59.8, 76.5)	30.0 (18.0, 36.5)	61.5 (52.8, 70.0)	19.0 (15.0, 30.0)	67.0 (56.0, 73.0)	23.0 (16.0, 31.5)	0.56	0.008	0.40	0.81	0.24
**48h**	66.0 (56.5, 76.0)	24.5 (15.8, 29.0)	66.5 (57.2, 73.8)	22.0 (15.0, 26.0)	64.0 (54.5, 70.0)	21.0 (15.0, 34.0)	0.17	<0.001	0.49	0.06	0.09

© 2024 Tryfonos A et al. *JAMA Network Open*.

	Exercise						P values				
	HIIT		MICT		ST		Δ change PCC vs. Control			Δ change all exercises	
	PCC	Control	PCC	Control	PCC	Control	HIIT	MICT	ST	PCC	Control
**POMS tension, median (IQR), score**											
**Pre**	6.0 (3.5, 11.0)	2.0 (1.0, 4.0)	5.0 (3.0, 10.2)	4.0 (2.0, 5.2)	5.0 (3.0, 10.0)	2.0 (1.0, 3.0)					
**Post**	6.5 (4.0, 9.0)	2.0 (1.0, 3.0)	5.0 (3.0, 7.2)	2.0 (1.0, 4.0)	5.0 (3.0, 9.0)	2.0 (1.0, 3.0)	0.22	0.51	0.27	0.55	0.02
**48h**	4.0 (1.5, 10.5)	1.5 (1.0, 3.0)	5.5 (2.0, 10.5)	2.0 (1.0, 4.0)	5.0 (2.5, 8.0)	1.0 (1.0, 4.0)	0.83	0.17	0.05	0.43	0.04
**POMS depression, median (IQR), score**											
**Pre**	7.0 (1.5, 12.0)	0.0 (0.0, 3.0)	4.5 (2.0, 11.5)	0.5 (0.0, 3.0)	6.0 (1.5, 9.0)	0.0 (0.0, 1.5)					
**Post**	5.0 (1.2, 12.2)	0.0 (0.0, 1.5)	3.5 (0.0, 7.5)	0.0 (0.0, 0.5)	5.0 (1.0, 9.0)	0.0 (0.0, 0.5)	0.31	0.49	0.93	0.17	0.71
**48h**	5.0 (2.0, 9.0)	0.0 (0.0, 1.0)	6.0 (1.8, 11.5)	0.0 (0.0, 0.5)	3.0 (0.0, 9.0)	0.0 (0.0, 1.0)	0.92	0.35	0.38	0.91	0.17
**POMS anger, median (IQR), score**											
**Pre**	2.0 (0.0, 5.5)	0.0 (0.0, 1.0)	0.5 (0.0, 5.2)	0.0 (0.0, 2.0)	1.0 (0.0, 4.5)	0.0 (0.0, 1.0)					
**Post**	0.0 (0.0, 3.8)	0.0 (0.0, 1.0)	0.5 (0.0, 4.2)	0.0 (0.0, 0.0)	0.0 (0.0, 2.0)	0.0 (0.0, 0.0)	0.07	0.65	0.16	0.54	0.23
**48h**	1.0 (0.0, 4.5)	0.0 (0.0, 1.0)	1.0 (0.0, 4.0)	0.0 (0.0, 0.0)	0.0 (0.0, 2.0)	0.0 (0.0, 0.0)	0.88	0.34	0.03	0.43	0.12
**POMS Vigor, median (IQR), score**											
**Pre**	9.0 (4.0, 13.5)	22.0 (18.5, 24.0)	6.5 (4.0, 14.0)	21.0 (16.8, 26.2)	9.0 (5.0, 14.0)	23.0 (18.0, 27.0)					
**Post**	6.5 (2.0, 12.2)	23.0 (18.5, 26.5)	6.5 (4.8, 10.2)	23.0 (18.0, 27.0)	6.0 (4.0, 12.5)	22.0 (19.0, 27.0)	0.14	0.26	0.17	0.99	0.93
**48h**	7.0 (3.0, 12.5)	22.5 (17.2, 26.8)	6.0 (3.8, 13.0)	23.0 (17.0, 26.0)	9.0 (3.5, 11.0)	23.0 (17.5, 25.5)	0.12	0.48	0.63	0.88	0.62
**POMS Fatigue, median (IQR), score**											
**Pre**	14.0 (10.0, 17.0)	2.0 (0.0, 3.0)	16.5 (10.0, 19.2)	2.0 (0.0, 4.0)	14.0 (10.0, 17.0)	1.0 (0.0, 4.0)					
**Post**	18.5 (13.0, 22.8)	3.0 (1.0, 8.0)	16.0 (11.5, 19.5)	1.0 (0.0, 5.0)	17.0 (12.5, 21.0)	3.0 (0.0, 6.0)	0.21	0.55	0.13	0.23	0.13
**48h**	15.0 (11.5, 18.0)	1.0 (0.0, 3.0)	14.0 (8.0, 18.0)	1.0 (0.0, 2.0)	15.0 (13.0, 17.0)	1.0 (0.0, 3.5)	0.13	0.95	0.68	0.22	0.42
**POMS Confusion, median (IQR), score**											
**Pre**	8.0 (5.0, 12.0)	3.0 (1.0, 4.0)	9.0 (6.0, 12.2)	2.5 (1.0, 5.0)	9.0 (7.0, 13.0)	2.0 (1.0, 3.0)					
**Post**	10.0 (6.2, 13.0)	1.0 (1.0, 4.5)	9.0 (7.8, 11.0)	1.0 (0.5, 3.0)	10.0 (7.0, 13.0)	1.0 (0.0, 3.5)	0.006	0.10	0.69	0.14	0.38
**48h**	10.0 (6.5, 12.5)	1.5 (1.0, 4.0)	10.0 (7.0, 13.0)	2.0 (0.0, 4.0)	9.0 (6.0, 12.0)	2.0 (1.0, 4.0)	0.03	0.009	0.13	0.04	0.08
**POMS Total Mood Disturbance, median (IQR), score**											
**Pre**	34.0 (15.0, 48.0)	-11.0 (-20.0, -4.5)	31.5 (8.0, 52.2)	-12.0 (-20.0, -2.0)	30.0 (21.0, 44.0)	-12.0 (-24.5, -4.5)					
**Post**	39.5 (19.0, 52.2)	-13.0 (-21.5, -2.0)	29.5 (13.0, 43.2)	-15.0 (-25.5, -3.5)	35.0 (15.0, 47.5)	-15.0 (-24.0, -4.5)	0.03	0.32	0.30	0.22	0.14
**48h**	32.0 (13.5, 49.0)	-16.5 (-23.8, -6.2)	33.5 (18.5, 52.8)	-15.0 (-22.5, -6.0)	28.0 (16.0, 45.5)	-15.0 (-22.0, -5.0)	0.28	0.21	0.38	0.81	0.13

© 2024 Tryfonos A et al. *JAMA Network Open*.

	Exercise						P values				
	HIIT		MICT		ST		Δ change PCC vs. Control			Δ change all exercises	
	PCC	Control	PCC	Control	PCC	Control	HIIT	MICT	ST	PCC	Control
**SPHERE SOMA, median (IQR), score**											
**Pre**	5.0 (4.0, 8.8)	0.0 (0.0, 1.5)	5.5 (4.0, 7.0)	0.0 (0.0, 1.0)	5.0 (4.0, 8.0)	0.0 (0.0, 1.0)					
**48h**	6.5 (4.2, 10.0)	0.0 (0.0, 0.5)	6.5 (4.2, 9.0)	0.0 (0.0, 0.0)	6.0 (4.0, 9.8)	0.0 (0.0, 1.0)	0.003	0.03	0.91	0.52	0.07
**SPHERE PHYSH, median (IQR), score**											
**Pre**	1.0 (0.0, 3.0)	0.0 (0.0, 0.0)	1.0 (0.2, 3.0)	0.0 (0.0, 0.0)	1.5 (0.0, 3.0)	0.0 (0.0, 0.0)					
**48h**	1.0 (0.0, 2.8)	0.0 (0.0, 0.0)	1.0 (0.0, 3.0)	0.0 (0.0, 0.0)	0.0 (0.0, 2.0)	0.0 (0.0, 0.0)	0.97	0.89	0.06	0.53	0.06

HIIT: high-intensity interval training; MICT: moderate-intensity continuous training; ST: strength training; MFI: Multidimensional Fatigue Inventory; POMS: Profile of Mood States (POMS); SPHERE: Somatic (SOMA) and Psychological (PHYCH) Health Report

© 2024 Tryfonos A et al. *JAMA Network Open*.

eTable 6: Mean difference in delta changes (Δ) in symptoms from baseline (Pre) to immediate post- and 48h post-exercise in patients with post-covid condition (PCC) and age- and sex-matched healthy controls as assessed by a) Multifactional Fatigue Inventory (MFI), b) Profile of Mood States (POMS), and c) the Somatic (SOMA) and Psychological (PHYCH) Health REport (SPHERE). Medians (IQR) for each symptom are presented in eTable 5. Data are presented as mean difference (95% CI).

	PCC vs. Control		
	**HIIT**	**MICT**	**ST**
	Mean difference (95% CI)	Mean difference (95% CI)	Mean difference (95% CI)
**MFI General Fatigue, score**			
Post-Pre	0.5 (-0.8, 1.8)	-0.3 (-2.2, 1.5)	0.1 (-1.4, 1.7)
48h-Pre	0.4 (-0.9, 1.8)	1.2 (-0.4, 2.7)	-0.5 (-1.9, 0.8)
**MFI Physical Fatigue, score**			
Post-Pre	-0.4 (-1.8, 1.0)	-0.6 (-2.1, 0.9)	-0.1 (-1.4, 1.1)
48h-Pre	0.4 (-0.9, 1.8)	1.5 (0.2, 2.8)	-1.4 (-2.7, -0.1)
**MFI Reduced Activity, score**			
Post-Pre	0.3 (-1.0, 1.6)	0.2 (-1.6, 1.9)	0.4 (-1.1, 1.8)
48h-Pre	1.3 (0.2, 2.3)	1.5 (0.1, 2.9)	-0.1 (-1.5, 1.4)
**MFI Reduced Motivation, score**			
Post-Pre	1.4 (-0.3, 3.1)	1.4 (-0.2, 2.9)	1.0 (-0.5, 2.5)
48h-Pre	0.6 (-1.2, 2.4)	2.5 (0.9, 4.1)	0.3 (-1.0, 1.6)
**MFI Mental Fatigue, score**			
Post-Pre	-0.5 (-1.6, 0.6)	0.7 (-1.0, 2.4)	-0.2 (-1.3, 0.8)
48h-Pre	0.9 (-0.6, 2.3)	2.6 (1.2, 4.0)	-0.6 (-1.4, 0.3)
**MFI Total, score**			
Post-Pre	1.2 (-3.0, 5.5)	1.3 (-4.8, 7.4)	1.2 (-3.9, 6.3)
48h-Pre	3.6 (-1.2, 8.5)	9.3 (3.8, 14.9)	-2.2 (-6.6, 2.2)
**POMS tension, score**			
Post-Pre	1.2 (-1.1, 3.6)	-0.2 (-2.3, 1.9)	-1.4 (-3.3, 0.4)
48h-Pre	-0.7 (-2.6, 1.2)	1.4 (-0.5, 3.3)	-1.8 (-3.9, 0.2)
**POMS depression, score**			
Post-Pre	3.5 (-0.0, 7.0)	-1.2 (-4.0, 1.5)	-0.3 (-3.0, 2.4)
48h-Pre	0.5 (-1.8, 2.9)	3.0 (-1.7, 7.8)	-1.9 (-4.9, 1.1)
**POMS anger, score**			
Post-Pre	-0.8 (-1.9, 0.3)	0.2 (-1.9, 2.4)	-1.4 (-3.7, 0.8)
48h-Pre	0.1 (-1.5, 1.7)	2.3 (-1.0, 5.6)	-2.6 (-5.1, -0.2)
**POMS Vigor, score**			
Post-Pre	-2.0 (-4.7, 0.6)	-1.5 (-3.6, 0.6)	-1.1 (-3.3, 1.1)
48h-Pre	-1.8 (-4.2, 0.6)	-0.3 (-3.1, 2.4)	-0.6 (-3.1, 1.9)
**POMS Fatigue, score**			
Post-Pre	2.2 (-0.6, 4.9)	0.1 (-2.9, 3.1)	1.2 (-2.0, 4.4)
48h-Pre	1.3 (-0.9, 3.5)	0.1 (-2.1, 2.2)	0.3 (-2.5, 3.2)
**POMS Confusion, score**			
Post-Pre	2.4 (0.4, 4.5)	0.9 (-0.6, 2.4)	0.2 (-1.2, 1.7)
48h-Pre	1.8 (0.2, 3.3)	1.9 (0.5, 3.2)	-1.5 (-3.3, 0.2)

© 2024 Tryfonos A et al. *JAMA Network Open*.

	PCC vs. Control		
	**HIIT**	**MICT**	**ST**
	Mean difference (95% CI)	Mean difference (95% CI)	Mean difference (95% CI)
**POMS Total Mood Disturbance, score**			
Post-Pre	11.8 (-1.4, 24.9)	1.1 (-9.2, 11.3)	-1.8 (-14.0, 10.4)
48h-Pre	4.6 (-2.9, 12.2)	9.0 (-0.4, 18.3)	-8.0 (-19.8, 3.8)
**SPHERE SOMA, score**			
48h-Pre	1.2 (0.4, 2.0)	0.8 (0.1, 1.6)	0.0 (-1.0, 1.1)
**SPHERE PHYCH, score**			
48h-Pre	0.2 (-0.4, 0.7)	0.1 (-0.6, 0.8)	-0.7 (-1.3, 0.0)

HIIT: high-intensity interval training; MICT: moderate-intensity continuous training; ST: strength training; MFI: Multidimensional Fatigue Inventory; POMS: Profile of Mood States (POMS); SPHERE: Somatic (SOMA) and Psychological (PHYCH) Health Report

© 2024 Tryfonos A et al. *JAMA Network Open*.

eTable 7. Data from 48h follow-up cardiopulmonary exercise testing (CPET) in patients with post-covid condition (PCC; N=31) and age-, sex-matched healthy controls (N=31). Data are presented as means and (SD). P values are presented for main effect of Group (G), Exercise (E), and Group x Exercise interaction (G*E).

	PCC			Controls			P values		
	**HIIT**	**MICT**	**ST**	**HIIT**	**MICT**	**ST**	**G**	**E**	**G*E**
HR rest, mean (SD), bpm	75 (11)	75 (10)	77 (9)	70 (9)	70 (9)	70 (8)	0.008	0.503	0.474
HR peak, mean (SD), bpm	170 (16)	170 (18)	171 (16)	170 (14)	168 (15)	171 (13)	0.792	0.336	0.656
RPE scale rest, mean (SD), score	7.1 (2.2)	7.5 (2.4)	7.3 (2.5)	6.1 (0.3)	6.1 (0.2)	6.2 (0.7)	0.005	0.487	0.168
RPE scale peak, mean (SD), score	19.1 (0.9)	18.7 (1.5)	18.7 (1.1)	18.6 (1.4)	18.7 (1.3)	18.6 (1.5)	0.473	0.574	0.454
SaO_2 rest, mean (SD), %	99 (1)	99 (1)	99 (1)	99 (1)	99 (1)	99 (1)	0.877	0.333	0.552
SaO_2 peak, mean (SD), %	95 (5)	96 (3)	95 (3)	94 (4)	94 (4)	94 (4)	0.031	0.990	0.967
Lactate rest, mean (SD), mmol/L	1.0 (0.4)	0.9 (0.3)	0.9 (0.3)	1.0 (0.3)	0.9 (0.2)	0.8 (0.3)	0.498	0.018	0.284
Lactate peak, mean (SD), mmol/L	9.4 (3.5)	9.0 (3.6)	9.0 (3.2)	9.0 (2.8)	8.8 (2.8)	9.9 (3.1)	0.883	0.325	0.096
VO_{2peak}, mean (SD), ml/kg/min	28.1 (8.0)	28.8 (8.5)	28.1 (7.5)	34.9 (8.4)	34.0 (7.1)	34.7 (8.2)	0.004	0.918	0.098
Workload peak, mean (SD), W	171 (67)	173 (68)	173 (68)	224 (67)	222 (72)	226 (69)	0.005	0.293	0.429
RER, mean (SD), ratio	1.21 (0.11)	1.21 (0.10)	1.19 (0.08)	1.21 (0.08)	1.20 (0.07)	1.20 (0.08)	0.994	0.146	0.397
VO_2 @ VT, mean (SD), ml/kg/min	16.8 (5.7)	17.7 (5.6)	16.8 (4.5)	19.9 (5.8)	19.7 (5.3)	20.3 (5.7)	0.045	0.815	0.401
VO_2 @VT, mean (SD), %	58.5 (10.4)	60.3 (7.7)	59.7 (8.0)	57.6 (11.5)	58.2 (10.5)	58.9 (9.8)	0.572	0.587	0.862
Workload @ VT, mean (SD), W	80 (44)	84 (35)	81 (32)	103 (32)	106 (38)	106 (40)	0.018	0.544	0.851
Workload @ VT, mean (SD), %	44.7 (11.1)	47.2 (9.3)	46.4 (7.2)	47.0 (9.7)	47.4 (8.8)	47.3 (10.1)	0.599	0.557	0.738
HR @ VT, mean (SD), bpm	117 (17)	118 (13)	117 (10)	116 (13)	112 (14)	116 (16)	0.607	0.964	0.971
HR @ VT, mean (SD), %	67.8 (9.1)	68.5 (7.5)	65.3 (15.2)	68.1 (9.1)	69.1 (8.0)	66.4 (15.1)	0.793	0.254	0.972
VO_2 @ OBLA, mean (SD), ml/kg/min	24.8 (6.5)	25.5 (6.2)	25.0 (6.2)	31.6 (6.3)	29.9 (5.9)	30.9 (6.4)	0.002	0.578	0.074
VO_2 @ OBLA, mean (SD), %	84.5 (10.0)	84.4 (9.7)	87.3 (8.5)	90.0 (6.6)	87.1 (8.9)	88.3 (7.8)	0.117	0.340	0.225
Workload @ OBLA, mean (SD), W	138 (51)	141 (50)	143 (53)	192 (37)	186 (57)	190 (52)	0.001	0.589	0.213
Workload @ OBLA, mean (SD), %	76.5 (9.2)	78.0 (8.7)	79.7 (8.1)	83.7 (7.6)	83.1 (9.4)	82.4 (7.4)	0.011	0.766	0.188
HR @ OBLA, mean (SD), bpm	151 (14)	153 (13)	154 (10)	156 (14)	155 (12)	156 (12)	0.364	0.605	0.607
HR @ OBLA, mean (SD), %	87.3 (5.7)	87.2 (6.4)	88.5 (5.2)	91.0 (5.7)	91.3 (6.1)	91.0 (5.8)	0.013	0.731	0.638

HIIT: High-intensity interval training; MICT: Moderate-intensity continuous training; ST: Strength training; HR: Heart rate; RPE: Rating of perceived exertion; RER: Respiratory exchange ratio; VO_2peak; peak volume of oxygen consumption; VT: Ventilatory threshold; OBLA: Onset of blood lactate accumulation at 4mmol/L

© 2024 Tryfonos A et al. *JAMA Network Open*.

eTable 8: Mean differences in variables from 48h follow-up cardiopulmonary exercise testing (CPET) between the patients with post-covid condition (PCC; N=31) and age-, sex- matched healthy controls (N=31). Means (SD) for each group are presented in eTable 7. Data are presented as mean difference (95% CI).

	**Group effect**		
	**HIIT** Mean difference (95% CI)	**MICT** Mean difference (95% CI)	**ST** Mean difference (95% CI)
HR rest, bpm	4.6 (1.9, 7.2)	5.2 (2.5, 7.8)	6.9 (4.5, 9.3)
HR peak, bpm	-1.3 (-5.3, 2.7)	0.7 (-4.0, 5.3)	1.4 (-2.3, 5.0)
RPE scale rest, score	1.2 (0.7, 1.6)	1.5 (1.0, 2.0)	1.3 (0.8, 1.8)
RPE scale peak, score	0.4 (0.1, 0.7)	0.1 (-0.3, 0.4)	0.2 (-0.1, 0.6)
SaO2 rest, %	-0.1 (-0.4, 0.2)	-0.2 (-0.5, 0.1)	0.6 (-1.8, 2.9)
SaO2 peak, %	1.5 (0.4, 2.6)	1.7 (0.7, 2.7)	-1.7 (-5.7, 2.4)
Lactate rest, mmol/L	-0.0 (-0.1, 0.1)	0.0 (-0.1, 0.1)	0.1 (0.0, 0.2)
Lactate peak, mmol/L	0.4 (-0.4, 1.2)	0.0 (-0.8, 0.9)	-0.7 (-1.6, 0.1)
VO2peak, ml/kg/min	-7.6 (-9.9, -5.3)	-5.5 (-7.8, -3.2)	-7.1 (-9.3, -4.8)
Workload peak, W	-57.7 (-76.4, -38.9)	-51.3 (-71.7, -30.9)	-53.1 (-72.1, -34.1)
RER, ratio	0.0 (-0.0, 0.0)	0.0 (-0.0, 0.0)	-0.0 (-0.0, 0.0)
VO2 @ VT, ml/kg/min	-3.3 (-4.9, -1.6)	-1.7 (-3.3, -0.2)	-25.9 (-36.3, -15.6)
VO2 @VT, %	0.8 (-2.1, 3.7)	2.6 (0.0, 5.2)	-2.5 (-4.1, -0.9)
Workload @ VT, W	1.4 (-2.8, 5.6)	-22.6 (-33.5, -11.8)	-38.5 (-53.8, -23.2)
Workload @ VT, %	-0.3 (-2.9, 2.2)	1.1 (-1.3, 3.5)	-2.3 (-4.4, -0.1)
HR @ VT, bpm	-2.4 (-5.1, 0.4)	0.1 (-2.5, 2.6)	-0.6 (-3.0, 1.7)
HR @ VT, %	-24.4 (-34.8, -14.0)	-41.4 (-58.2, -24.6)	-0.7 (-3.8, 2.4)
VO2 @ OBLA, ml/kg/min	-5.8 (-7.8, -3.8)	3.1 (-1.0, 7.2)	1.3 (0.3, 2.2)
VO2 @ OBLA, %	-6.3 (-8.8, -3.7)	-2.2 (-5.1, 0.6)	0.6 (-3.1, 4.2)
Workload @ OBLA, W	-38.2 (-52.0, -24.5)	-2.5 (-6.5, 1.5)	-5.3 (-7.2, -3.4)
Workload @ OBLA, %	-7.5 (-10.2, -4.9)	-4.9 (-7.7, -2.1)	-1.2 (-3.5, 1.0)
HR @ OBLA, bpm	-4.1 (-8.4, 0.1)	-4.1 (-6.0, -2.2)	0.2 (-0.1, 0.4)
HR @ OBLA, %	-3.8 (-5.6, -1.9)	-3.9 (-6.0, -1.8)	-3.6 (-5.1, -2.1)

HIIT: High-intensity interval training; MICT: Moderate-intensity continuous training; ST: Strength training; HR: Heart rate; RPE: Rating of perceived exertion; RER: Respiratory exchange ratio; VO_2peak; peak volume of oxygen consumption; VT: Ventilatory threshold; OBLA: Onset of blood lactate accumulation at 4mmol/L

© 2024 Tryfonos A et al. *JAMA Network Open*.

eTable 9: Mean differences in variables from the 48h follow-up cardiopulmonary exercise testing (CPET) between the three exercise sessions (HIIT: high-intensity interval training, MICT: moderate-intensity continuous training, ST: strength training). Data are combined patients with post-covid condition (PCC; N=31) and age-, sex- matched healthy controls (N=31). Means (SD) for each group and exercise are presented in eTable 7. Data are presented as mean difference (95% CI).

	**Exercise effect**		
	**HIIT vs. MICT** Mean difference (95% CI)	**HIIT vs. ST** Mean difference (95% CI)	**MICT vs. ST** Mean difference (95% CI)
HR rest, bpm	-0.7 (-4.5, 3.0)	-1.2 (-4.8, 2.4)	-0.5 (-4.1, 3.1)
HR peak, bpm	1.6 (-4.5, 7.8)	0.1 (-5.3, 5.5)	-1.5 (-7.5, 4.4)
RPE scale rest, score	-0.1 (-0.8, 0.5)	-0.1 (-0.8, 0.5)	0.0 (-0.7, 0.7)
RPE scale peak, score	0.1 (-0.4, 0.6)	0.2 (-0.3, 0.6)	0.0 (-0.5, 0.5)
SaO2 rest, %	-0.1 (-0.6, 0.3)	-0.2 (-0.6, 0.2)	-0.1 (-0.5, 0.3)
SaO2 peak, %	-0.2 (-1.6, 1.3)	-0.1 (-1.5, 1.4)	0.1 (-1.2, 1.5)
Lactate rest, mmol/L	0.1 (-0.0, 0.2)	0.1 (0.0, 0.2)	0.0 (-0.1, 0.1)
Lactate peak, mmol/L	0.2 (-1.0, 1.4)	-0.4 (-1.6, 0.7)	-0.6 (-1.8, 0.6)
VO2peak, ml/kg/min	0.8 (-2.4, 4.1)	0.3 (-3.0, 3.5)	-0.6 (-3.8, 2.6)
Workload peak, W	4.0 (-23.7, 31.7)	-5.3 (-32.0, 21.4)	-9.2 (-37.1, 18.7)
RER, ratio	0.0 (-0.0, 0.0)	0.0 (-0.0, 0.0)	0.0 (-0.0, 0.0)
VO2 @ VT, ml/kg/min	0.2 (-2.1, 2.4)	-0.0 (-2.2, 2.2)	-0.2 (-2.3, 1.9)
VO2 @VT, %	-1.1 (-5.0, 2.8)	-1.2 (-4.9, 2.6)	-0.0 (-3.5, 3.5)
Workload @ VT, W	1.0 (-14.0, 16.1)	-2.6 (-17.3, 12.0)	-3.7 (-18.7, 11.3)
Workload @ VT, %	-0.9 (-4.7, 2.9)	-0.9 (-4.5, 2.8)	0.0 (-3.5, 3.5)
HR @ VT, bpm	-1.2 (-7.1, 4.7)	-0.6 (-6.2, 4.9)	0.6 (-4.9, 6.1)
HR @ VT, %	-1.6 (-5.0, 1.9)	1.9 (-2.9, 6.6)	3.4 (-1.2, 8.1)
VO2 @ OBLA, ml/kg/min	0.1 (-2.7, 2.9)	0.6 (-2.2, 3.3)	0.4 (-2.2, 3.1)
VO2 @ OBLA, %	1.0 (-2.8, 4.8)	-0.3 (-3.7, 3.1)	-1.3 (-4.9, 2.3)
Workload @ OBLA, W	-8.8 (-30.6, 12.9)	-7.2 (-27.8, 13.3)	1.6 (-21.2, 24.4)
Workload @ OBLA, %	-0.8 (-4.6, 3.0)	-0.8 (-4.2, 2.6)	-0.1 (-3.6, 3.4)
HR @ OBLA, bpm	-1.5 (-7.3, 4.3)	-2.0 (-7.2, 3.3)	-0.4 (-5.5, 4.6)
HR @ OBLA, %	-0.5 (-3.3, 2.3)	-1.0 (-3.5, 1.4)	-0.5 (-3.1, 2.1)

HIIT: High-intensity interval training; MICT: Moderate-intensity continuous training; ST: Strength training; HR: Heart rate; RPE: Rating of perceived exertion; RER: Respiratory exchange ratio; VO_2peak; peak volume of oxygen consumption; VT: Ventilatory threshold; OBLA: Onset of blood lactate accumulation at 4mmol/L

© 2024 Tryfonos A et al. *JAMA Network Open*.

eTable 10: Interleukin (IL)-6 and Creatine kinase (CK) status pre-, immediately post- and 48h post-exercise in PCC and well-matched healthy controls. CK was only measured pre- and 48h post-exercise. Data are presented as mean and (SD). P values are presented for main effect of Group (G), Exercise (E), Time (T), and Group x Exercise interaction (G*E), Group x Time interaction (G*T), Exercise x Time interaction (E*T), Group x Exercise x Time interaction (G*E*T)

	**HIIT**		**MICT**		**ST**		**P values**						
	PCC	Control	PCC	Control	PCC	Control	G	E	T	G*E	G*T	E*T	G*E*T
**IL-6, mean (SD), pg/mL**													
Pre	1.1 (1.1)	1.1 (0.9)	1.1 (1.0)	1.2 (1.2)	0.9 (0.8)	0.7 (0.8)	0.372	0.180	0.017	0.039	0.491	0.306	0.916
Post	1.6 (1.6)	1.0 (1.2)	1.2 (1.0)	1.4 (1.2)	1.1 (1.1)	0.8 (0.9)							
48h	1.5 (2.0)	1.2 (1.0)	1.1 (1.2)	1.5 (1.0)	1.6 (1.3)	1.2 (1.1)							
**CK, mean (SD), microkat/L**													
Pre	1.4 (0.8)	1.9 (1.3)	1.5 (0.9)	2.1 (1.2)	1.9 (1.5)	2.0 (1.3)	0.057	<0.001	0.267	0.522	0.255	0.004	0.054
48h	1.3 (0.7)	1.8 (0.9)	1.4 (0.7)	1.7 (0.9)	2.0 (1.4)	3.3 (3.5)							

HIIT: High-intensity interval training; MICT: Moderate-intensity continuous training; ST: Strength training

© 2024 Tryfonos A et al. *JAMA Network Open*.

eTable 11: Mean group differences in participants' characteristics and results from the physiological assessment presented in Table 1 (manuscript). Data included 31 patients with post-COVID-19 condition (PCC) and 31 age- and sex-matched healthy controls. Results are presented as mean group difference (95% CI).

	**Mean group difference (95% CI)**
Age, years	-0.68 (-5.58, 4.23)
BMI, m^2/Kg	0.19 (-1.49, 1.88)
BSA, m^2	-0.02 (-0.12, 0.09)
**GSLTPHQ**	
Current self-reported PA, score	-28.08 (-39.70, -16.46)
**SF-36 Questionnaire**	
Physical Function, score	-42.14 (-49.41, -34.87)
Physical Role, score	-83.06 (-94.16, -71.97)
Emotional Role, score	-23.66 (-38.61, -8.70)
Mental health, score	-53.23 (-62.09, -44.36)
Social Function, score	-17.29 (-25.04, -9.54)
Bodily Pain, score	-54.44 (-64.81, -44.06)
Vitality, score	-43.06 (-54.04, -32.09)
General health, score	-44.52 (-52.31, -36.72)
**Physical function**	
6-min walk, m	-32.67 (-71.29, 5.95)
Isokinetic 60 deg/s, N*m	-19.00 (-46.02, 8.02)
Isokinetic 210 deg/s, N*m	-8.08 (-27.59, 11.42)
Isometric 120 deg, N*m	-36.60 (-67.33, -5.86)
Handgrip MVC, Kg	-4.26 (-11.41, 2.90)
**Accelerometers**	
MVPA, min/day	-26.54 (-42.32, -10.77)
Total PA, min/day	9.75 (-95.51, 115.01)
Sedentary time, min/day	13.29 (-29.68, 56.25)
**Blood/Plasma volume**	
Plasma volume, ml/kg	-3.40 (-7.41, 0.61)
Blood volume, ml/kg	-5.23 (-11.92, 1.47)
**Blood biomarkers**	
Leukocytes, x10(9)/L	0.06 (-0.74, 0.87)
Erythrocytes, x10(12)/L	-0.00 (-0.23, 0.22)
Thrombocytes, x10(9)/L	-9.79 (-45.62, 26.03)
Hemoglobin, g/L	0.74 (-4.91, 6.38)
EVF, ratio	-0.00 (-0.02, 0.01)
Erc(B)-MCH, fL	0.16 (-0.58, 0.90)
Erc(B)-MCV, pg	-1.33 (-3.47, 0.82)
CRP, mg/L	-0.47 (-1.31, 0.37)
Glucose, mmol/L	0.18 (-0.14, 0.50)
Triglycerides, mmol/L	0.44 (-0.04, 0.93)
Total cholesterol, mmol/L	0.25 (-0.29, 0.78)
Iron, micromole/L	0.10 (-3.42, 3.62)
Transferrin, g/L	-0.02 (-0.22, 0.18)

© 2024 Tryfonos A et al. *JAMA Network Open*.

	Mean group difference (95% CI)
Transferrin sat, ratio	-0.01 (-0.07, 0.06)
**Spirometry**	
FVC, L	-0.29 (-0.87, 0.28)
FEV_1, L	-0.09 (-0.55, 0.36)
VC, L	-0.40 (-0.96, 0.16)
FEV_1/VC, ratio	0.01 (-0.05, 0.07)
RV, L	-0.20 (-0.45, 0.05)
TLC, L	-0.59 (-1.32, 0.13)
RV/TLC, ratio	0.13 (-2.56, 2.82)
DLCOc SB, mmol/(min*kPa)	-0.91 (-2.10, 0.28)
**Echocardiography**	
Stroke volume, ml	-9.73 (-18.21, -1.25)
Stroke index, ml/Kg	-4.93 (-8.53, -1.34)
LVEF, %	-0.35 (-2.00, 1.29)
Cardiac output, L	-0.28 (-0.82, 0.25)
Cardiac index, L/Kg	-0.12 (-0.35, 0.11)
LV diameter, mm	-2.59 (-5.04, -0.14)
TAPSE, mm	-1.95 (-4.00, 0.09)
E/A, ratio	-0.20 (-0.46, 0.06)
**Arteriography**	
PWV_{ao}, m/s	0.73 (0.02, 1.43)
**CPET**	
$VO_{2\ peak}$, L/min	-0.54 (-0.90, -0.17)
$VO_{2\ peak}$, ml/kg/min	-6.79 (-10.77, -2.82)
Workload peak, W	-47.32 (-80.71, -13.93)
HR peak, bpm	0.81 (-6.09, 7.70)
RER, ratio	0.00 (-0.04, 0.04)
SaO_2 rest, %	0.07 (-0.45, 0.59)
SaO_2 peak, %	0.52 (-0.49, 1.52)
RPE rest, score	0.62 (-0.08, 1.32)
RPE peak, score	0.42 (-0.06, 0.90)
Lactate rest, mmol/L	0.01 (-0.19, 0.20)
Lactate peak, mmol/L	-0.62 (-2.20, 0.96)
VO_2 @ VT, ml/kg/min	-2.62 (-5.42, 0.19)
VO_2 @ VT, %	2.58 (-2.65, 7.81)
VO_2 @ OBLA, ml/kg/min	-4.04 (-7.63, -0.45)
VO_2 @ OBLA, %	0.91 (-4.04, 5.87)
**Head-up TILT test (HUTT)**	
HR supine, bpm	6.99 (2.10, 11.89)
HR HUTT, bpm	11.16 (4.57, 17.75)
SBP supine, mmHg	2.96 (-3.53, 9.46)
SBP HUTT, mmHg	4.88 (-3.34, 13.11)
DBP supine, mmHg	2.27 (-2.63, 7.17)
DBP HUTT, mmHg	6.37 (-0.78, 13.53)
**Heart rate variability**	
Normal breathing, %	-1.19 (-5.63, 3.24)
Deep breathing, %	-5.19 (-10.95, 0.57)

© 2024 Tryfonos A et al. *JAMA Network Open*.

	Mean group difference (95% CI)
**Sympathetic Skin Response**	
Hand latency, s	0.01 (-0.11, 0.14)
Foot latency, s	0.12 (-0.07, 031)
**Nerve conduction studies**	
**Sural n.**	
SNAP, uV	1.76 (-3.76, 7.29)
CV, m/s	1.18 (-2.69, 5.05)
**Fibular n.**	
CMAP, mV	-0.76 (-1.95, 0.42)
CV, m/s	0.29 (-1.37, 1.95)
**Tibial n.**	
CMAP, mV	2.43 (0.33, 4.53)
CV, m/S	-0.47 (-2.42, 1.48)

Abbreviations: BMI: body mass index; BSA: body surface area; GSLTPA: Godin-Sheperd Leisure Time Physical Activity Questionnaire; MVC: maximal voluntary contraction; PA: physical activity; MVPA: moderate and vigorous PA; EVF: erythrocyte volume fraction; Erc(B)-MCH: erythrocyte-mean corpuscular hemoglobin concentration; Erc(B)-MCV: erythrocyte-mean corpuscular volume; FVC: forced vital capacity; FEV1: forced expiratory volume in the first second; VC: vital capacity; RV: residual volume; TLC: total lung capacity; DLCOc SB; diffusion capacity of the lung for CO single breath; LV: left ventricular; LVEF; LV ejection fraction; TAPSE; tricuspid annular plane systolic excursion, E/A: early (E) to late (A) ventricular filling velocities ratio; PWV_{ao}: pulse wave velocity aortic; HR; heart rate; VO_2: volume of oxygen consumption; RPE: rating of perceived exertion using Borg 6–20 scale, RER; respiratory exchange ratio; VT: ventilatory threshold; OBLA: onset of blood lactate accumulation; SBP: systolic blood pressure; DBP: diastolic blood pressure; SNAP: sensory nerve action potential; CV: conduction velocity; CMAP: compound muscle action potential

© 2024 Tryfonos A et al. *JAMA Network Open*.

eTable 12. Additional data from the clinical assessment in post-covid condition (PCC; N=31) and age-, sex- matched healthy controls (N=31). Data are presented as mean (SD).

	PCC	Controls	P value
**Accelerometers**			
Light PA, mean (SD), min/day	273 (253)	245 (62)	0.572
Moderate PA, mean (SD), min/day	33 (24)	53 (21)	0.001
Vigorous PA, mean (SD), min/day	3 (11)	8 (18)	0.210
**Blood volume**			
Hemoglobin mass, mean (SD), g/Kg	8.4 (1.8)	8.9 (1.6)	0.246
Erythrocyte volume, mean (SD), ml/kg	25.7 (5.4)	27.5 (4.9)	0.229
**Nerve conduction studies**			
**Ulnar n.**			
CMAP, mean (SD), mV	9.5 (1.6)	9.9 (1.7)	0.449
Motor CV, mean (SD), m/s	61.7 (4.6)	60.9 (5.2)	0.559
F-wave, mean (SD), ms	23.2 (2.1)	23.7 (2.2)	0.337
SNAP, mean (SD), uV	20.8 (7.8)	22.6 (13.1)	0.534
Sensory CV, mean (SD), m/s	59.2 (5.2)	59.2 (6.2)	0.812
**Median n.**			
CMAP, mean (SD), mV	8.3 (2.8)	7.4 (1.8)	0.178
Motor CV, mean (SD), m/s	55.7 (3.0)	55.5 (2.8)	0.854
F-wave, mean (SD), ms	22.7 (1.4)	23.1 (2.0)	0.362
SNAP, mean (SD), uV	30.2 (8.7)	34.6 (13.8)	0.146
Sensory CV, mean (SD), m/s	59.0 (6.5)	57.8 (5.7)	0.540
**Fibular Sup. n.**			
SNAP, mean (SD), uV	10.1 (4.4)	9.3 (5.6)	0.555
CV, mean (SD), m/s	57.8 (6.8)	56.6 (6.6)	0.464
**Needle EMG**			
**Deltoideus m.**			
Duration, mean (SD), ms	6.6 (0.9)	6.6 (0.8)	0.866
Amplitude, mean (SD), uV	948 (283)	977 (196)	0.650
Polyphasia, mean (SD), %	16.1 (13.5)	9.8 (9.8)	0.048
**Biceps br. m.**			
Duration, mean (SD), ms	6.3 (1.0)	6.5 (1.0)	0.324
Amplitude, mean (SD), uV	916 (350)	854 (180)	0.404
Polyphasia, mean (SD), %	14.6 (15.1)	10.6 (15.3)	0.223
**Trapezius m.**			
Duration, mean (SD), ms	6.5 (1.2)	6.9 (1.1)	0.289
Amplitude, mean (SD), uV	1095 (294)	1027 (244)	0.345
Polyphasia, mean (SD), %	14.7 (14.6)	10.8 (9.2)	0.225
**Tibialis ant. m.**			
Duration, mean (SD), ms	6.8 (1.1)	7.1 (1.2)	0.329

© 2024 Tryfonos A et al. *JAMA Network Open*.

	PCC	Controls	P value
Amplitude, mean (SD), uV	1147 (298)	1388 (559)	0.047
Polyphasia, mean (SD), %	18.3 (14.4)	9.8 (6.6)	0.007
**Vastus med. m.**			
Duration, mean (SD), ms	6.7 (0.9)	7.1 (1.3)	0.143
Amplitude, mean (SD), uV	1337 (556)	1328 (425)	0.945
Polyphasia, mean (SD), %	10.5 (11.5)	7.2 (9.5)	0.245
**Adductor magn. m.**			
Duration, mean (SD), ms	5.4 (1.0)	5.4 (1.5)	0.910
Amplitude, mean (SD), uV	853 (233)	871 (341)	0.850
Polyphasia, mean (SD), %	21.5 (15.5)	11.1 (11.2)	0.007

PA: Physical activity; SNAP: sensory nerve action potential; CV: conduction velocity; CMAP: compound muscle action potential

© 2024 Tryfonos A et al. *JAMA Network Open*.

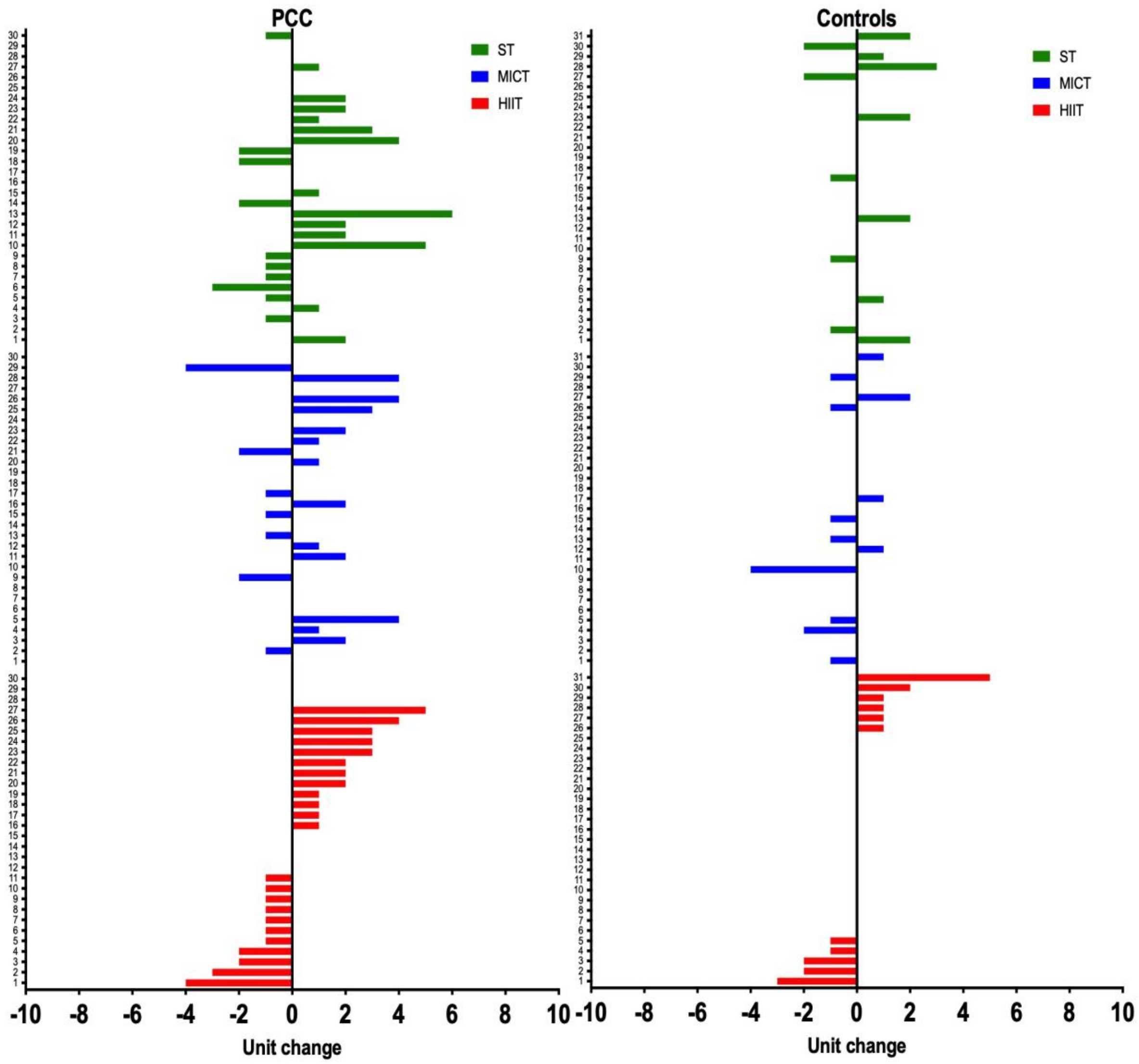

eFigure 1: Individual delta changes in fatigue from baseline to immediate post- and 48h after each exercise (HIIT: high-intensity interval training; MICT: moderate-intensity continuous training; ST: strength training) in patients with post-covid condition (PCC) and age- and sex-matched healthy controls, as assessed via visual analog scale (VAS). Ascending numbers were used to sort the subjects based on their responses to HIIT (smaller unit changes to larger unit changes).

© 2024 Tryfonos A et al. *JAMA Network Open*.

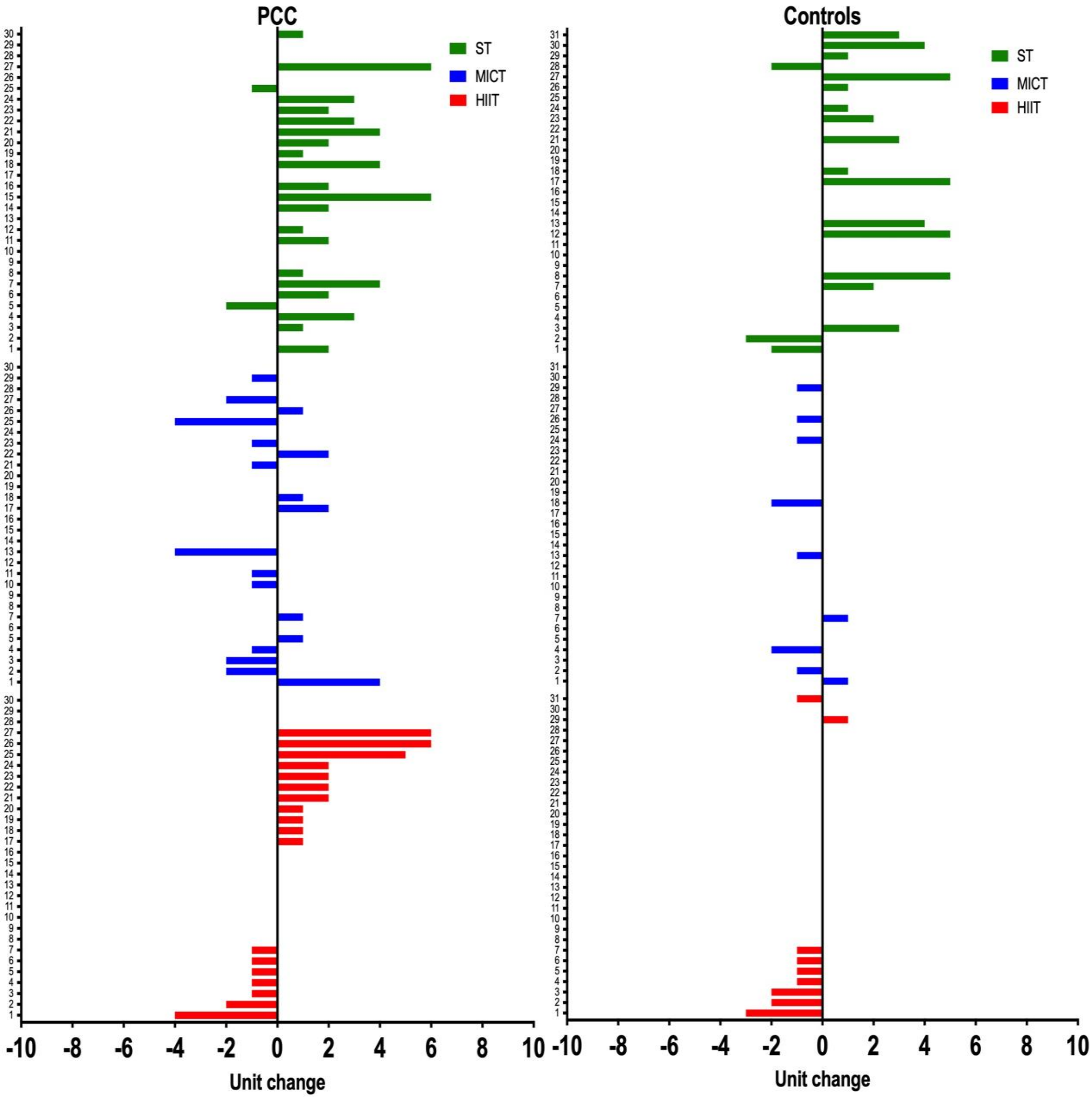

eFigure 2: Individual delta changes in muscle pain from baseline to immediate post- and 48h after each exercise (HIIT: high-intensity interval training; MICT: moderate-intensity continuous training; ST: strength training) in patients with post-covid condition (PCC) and age- and sex-matched healthy controls, as assessed via visual analog scale (VAS). Ascending numbers were used to sort the subjects based on their responses to HIIT (smaller unit changes to larger unit changes

© 2024 Tryfonos A et al. *JAMA Network Open*.
