# Supplementary material for: Functional Limitations and Exercise Intolerance in Patients With Post-COVID Condition: A Randomized Crossover Clinical Trial
Source: JAMA Netw Open. 2024 Apr 4;7(4):e244386. doi: 10.1001/jamanetworkopen.2024.4386 (PMC11192186; doi:10.1001/jamanetworkopen.2024.4386)
Supplement: Supplement 3. — Data Sharing Statement [file jamanetwopen-e244386-s003.pdf]

Data Sharing Statement

Tryfonos. Functional Limitations and Exercise Intolerance in Patients With Post-COVID Condition. *JAMA Netw Open*. Published April 04, 2024.
doi:10.1001/jamanetworkopen.2024.4386

Data
Data available: Yes
Data types: Deidentified participant data
How to access data: Data will be available upon resonable request to corresponding author (andrea.tryfonos@ki.se).
When available: With publication

Supporting Documents
Document types: Statistical/analytic code, Informed consent form, Other (please specify)
Additional Information: Supplement 1 - Clinical protocol, inclduing statistical plan and informed consent form Supplement 2 - Supplementary Tables and Figures availble online
How to access documents: The above supporting documents are attached with the current manuscript and they will be publically available with publication.
When available: With publication

Additional Information
Who can access the data: Researchers whose proposed use of the data has been approved.
Types of analyses: For any purpose, upon resonable request.
Mechanisms of data availability: With a signed data access agreement.
